# Genetic data and cognitively defined late-onset Alzheimer’s disease subgroups

**DOI:** 10.1038/s41380-018-0298-8

**Published:** 2018-12-04

**Authors:** Shubhabrata Mukherjee, Jesse Mez, Emily H. Trittschuh, Andrew J. Saykin, Laura E. Gibbons, David W. Fardo, Madeline Wessels, Julianna Bauman, Mackenzie Moore, Seo-Eun Choi, Alden L. Gross, Joanne Rich, Diana K. N. Louden, R. Elizabeth Sanders, Thomas J. Grabowski, Thomas D. Bird, Susan M. McCurry, Beth E. Snitz, M. Ilyas Kamboh, Oscar L. Lopez, Philip L. De Jager, David A. Bennett, C. Dirk Keene, Eric B. Larson, EPAD Study Group, Investigators from ACT, Investigators from ROS, Investigators from MAP, Investigators from ADNI, Investigators from the University of Pittsburgh ADRC, Paul K. Crane

**Affiliations:** 1 Department of Medicine, School of Medicine, University of Washington, Seattle, WA USA; 2 Department of Neurology, Boston University School of Medicine, Boston, MA USA; 3 Department of Psychiatry and Behavioral Sciences, School of Medicine, University of Washington, Seattle, WA USA; 4 VA Puget Sound Health Care System, Seattle, WA USA; 5 Department of Radiology, Indiana University, Indianapolis, IN USA; 6 Department of Biostatistics, University of Kentucky, Lexington, KY USA; 7 College of Arts and Sciences, School of Nursing, University of Washington, Seattle, WA USA; 8 Department of Epidemiology, Johns Hopkins Bloomberg School of Public Health, Baltimore, MD USA; 9 Health Sciences Library, University Libraries, University of Washington, Seattle, WA USA; 10 Department of Neurology, School of Medicine, University of Washington, Seattle, WA USA; 11 Department of Radiology, School of Medicine, University of Washington, Seattle, WA USA; 12 Department of Psychosocial and Community Health, School of Nursing, University of Washington, Seattle, WA USA; 13 Department of Neurology, University of Pittsburgh, Pittsburgh, PA USA; 14 Departments of Human Genetics, University of Pittsburgh, Pittsburgh, PA USA; 15 Departments of Psychiatry, University of Pittsburgh, Pittsburgh, PA USA; 16 Center for Translational & Computational Neuroimmunology, Department of Neurology, Columbia University Medical Center, New York, NY USA; 17 Rush Alzheimer’s Disease Center, Rush University Medical Center, Chicago, IL USA; 18 Department of Pathology, School of Medicine, The University of Washington, Seattle, WA USA; 19 Kaiser Permanente Washington Health Research Institute, Seattle, WA USA

**Keywords:** Psychology, Genetics

## Abstract

Categorizing people with late-onset Alzheimer’s disease into biologically coherent subgroups is important for personalized medicine. We evaluated data from five studies (total *n* = 4050, of whom 2431 had genome-wide single-nucleotide polymorphism (SNP) data). We assigned people to cognitively defined subgroups on the basis of relative performance in memory, executive functioning, visuospatial functioning, and language at the time of Alzheimer’s disease diagnosis. We compared genotype frequencies for each subgroup to those from cognitively normal elderly controls. We focused on *APOE* and on SNPs with *p* < 10^−5^ and odds ratios more extreme than those previously reported for Alzheimer’s disease (<0.77 or >1.30). There was substantial variation across studies in the proportions of people in each subgroup. In each study, higher proportions of people with isolated substantial relative memory impairment had ≥1 *APOE* ε4 allele than any other subgroup (overall *p* = 1.5 × 10^−27^). Across subgroups, there were 33 novel suggestive loci across the genome with *p* < 10^−5^ and an extreme OR compared to controls, of which none had statistical evidence of heterogeneity and 30 had ORs in the same direction across all datasets. These data support the biological coherence of cognitively defined subgroups and nominate novel genetic loci.

## Introduction

Clinical heterogeneity is common among people with late-onset Alzheimer’s disease (see [1] for a review). Categorizing people with a condition into biologically coherent subgroups is an important personalized medicine strategy [2]. This strategy is particularly recommended for neurodegenerative conditions [3]. Once biologically coherent subgroups are identified, further investigations may elucidate subgroup-specific treatments.

Genetic data may be useful for determining whether a proposed categorization strategy results in biologically coherent subgroups (see the Box 1).

We have developed an approach for categorizing people with late-onset Alzheimer’s disease based on relative performance across cognitive domains. We determine each person’s average performance at diagnosis across memory, executive functioning, language, and visuospatial ability, and consider relative impairments in each domain from that average. We previously evaluated one study’s data and showed that our strategy identified a subgroup with higher degrees of amyloid angiopathy and higher proportions with≥1 *APOE* ε4 allele [4].

Here, we evaluate data from five studies with people with late-onset Alzheimer’s disease [5] and cognitively normal elderly controls [6]. We used modern psychometric approaches to co-calibrate cognitive scores. We used scores to identify subgroups. We used genetic data to determine whether our categorization identifies biologically coherent subgroups.

Box 1 Schematic representation of incoherent vs. coherent subgrouping The large group at the top represents a heterogeneous group of individuals. A strategy is applied to categorize individuals into subgroups. For a precision medicine approach to work, the categorization should reduce heterogeneity. In the lower left figure, the method did not reduce heterogeneity and thus we refer to this as an *incoherent subgrouping strategy*. In contrast, the lower right figure was produced by a different method which resulted in relatively homogenous subgroups; this method would represent a *coherent subgrouping strategy*.

For incoherent subgroup comparisons with controls, top genetic hits, and effect sizes would not be expected to be different than those observed in the entire group. Further, for a given incoherent subgroup, spurious genetic associations at a locus would not be expected to replicate in that subgroup in other datasets. In contrast, for coherent subgroup comparisons with controls, there is improved potential for identification of novel loci and effect sizes could be stronger than those seen for the original ungrouped data. Further replication of subgroup associations in other datasets would occur more often than expected by chance.

Genetic data may serve a useful role in determining whether a categorization strategy produces biologically coherent subgroups.

## Materials and methods

### Study design and participants

We used data from the Adult Changes in Thought (ACT) study, the Alzheimer’s Disease Neuroimaging Initiative (ADNI), the Rush Memory and Aging Project (MAP) and Religious Orders Study (ROS), and the University of Pittsburgh Alzheimer Disease Research Center (PITT). Each study has published widely, and their genetic data are included in large analyses of late-onset Alzheimer’s disease [6, 7]. All five studies use the same research criteria to define clinical Alzheimer’s disease [5]. Three studies (ACT, MAP, and ROS) are prospective cohort studies that enroll cognitively normal elderly individuals and follow them to identify incident dementia cases. For these, we analyzed cognitive data from the visit with the incident clinical Alzheimer’s disease diagnosis. Two studies (ADNI and PITT) are clinic-based research cohort studies. For those studies we analyzed cognitive data from the first study visit for people with prevalent Alzheimer’s disease; we limited inclusion to those with Clinical Dementia Rating Scale [8] of 0.5 or 1, indicating mild Alzheimer’s disease. For people from ADNI or PITT who did not initially have Alzheimer’s disease, we analyzed cognitive data from the incident Alzheimer’s disease visit. The University of Washington IRB approved these secondary analyses of coded data.

In each study, we included people diagnosed with late-onset Alzheimer’s disease as defined by research criteria [5]. We used data from everyone for all analyses other than genetic analyses; we limited those to self-reported whites. For genetic analyses we also used data from self-reported white cognitively normal elderly controls from each study. Details on those cohorts are included in reports from the parent studies and in Supplementary Text 6 (derived from Lambert et al. [6]).

### Cognitive data procedures

Staff from each study administered a comprehensive neuropsychological test battery that included assessment of memory, executive functioning, language, and visuospatial functioning. We obtained granular (“item-level”) data from each parent study. Each stimulus administered to a participant was deemed an “item”. As outlined in our previous paper [2], every item administered in each study was considered by our panel of experts (JM, ET, AJS). Our panel designated each item as primarily a measure of memory, executive functioning, language, visuospatial functioning, or none of these. They also assigned items to theory-driven subdomains.

We carefully considered items where the same stimulus was administered to participants across different studies to identify “anchor items” that could anchor metrics across studies. We reviewed response categories recorded by each study for these overlapping items to ensure that consistent scoring was used across studies. We identified anchor items as those overlapping items with identical scoring across studies. We used bifactor models in Mplus [9] to co-calibrate separate scores for memory, executive functioning, language, and visuospatial functioning.

Details of item assignments, psychometric analyses in each study, and co-calibration methodology across studies are provided in Supplementary Texts 1, 2, and 5. Code is available from the authors upon request.

We used the ACT sample of people with incident Alzheimer’s disease as our reference population for the purpose of scaling domain scores; ACT was our largest sample from a prospective cohort study of people with late-onset Alzheimer’s disease. We *z*-transformed scores from other studies to ACT-defined metrics for each domain.

### Assignment to subgroups

We assigned people to subgroups as we have done previously [4]. Briefly, for each person we determined their average score across memory, executive functioning, language, and visuospatial functioning. We determined differences between each domain score and this average score. We identified domains with substantial relative impairments as those with relative impairments at least as large as 0.80 standard deviation units as explained in Supplementary Text 3. We categorized people by the number of domains with substantial relative impairments (0, 1, or ≥2) and further categorized those with substantial relative impairments in a single domain by the domain with a substantial relative impairment. This approach results in six potential subgroups: those with no domain with a substantial relative impairment; those with an isolated substantial relative impairment in one of four domains (e.g., isolated substantial relative memory impairment, isolated substantial relative language impairment, etc.), and those with multiple domains with substantial relative impairments.

### Statistical analyses

As in our previous study [4], we compared the proportion of people with late-onset Alzheimer’s disease in each subgroup with at least one *APOE* ε4 allele. For other genetic analyses we combined data from ROS and MAP, as has been done many times previously, and evaluated data separately in four genetic datasets. Each dataset was imputed using IMPUTE2 and samples of European ancestry from the 2012 build of the 1000 Genomes project. We excluded SNPs with *R*^2^ or information scores < 0.5, and SNPs with a minor allele frequency <3%. Further details are provided in Supplementary Text 6 and in Lambert et al. [6]

We used KING-Robust [10] to obtain study-specific principal components to account for population stratification. We used logistic regression in PLINK v 1.9 [11] to evaluate associations at each SNP for each cognitively defined subgroup. Cognitively normal elderly controls from each study were the comparison group for all of these analyses. We included covariates for age, sex, and principal components. We used METAL [12] for meta-analysis.

IGAP’s most extreme odds ratio (OR) outside of chromosome 19 was for rs11218343 associated with *SORL1*, which had an OR of 0.77 reported in the Stages 1 and 2 meta-analysis [6]. We focused attention on SNPs where meta-analysis ORs for any cognitively defined subgroup were < 0.77 (or, equivalently, ORs > [1/0.77], which is >1.30). As presented in the Box 1, more extreme ORs in a single subgroup, with strong replication across genetic datasets, would represent strong support of biologically coherent categorization.

For genetic loci previously identified as associated with risk of late-onset Alzheimer’s disease, we used the methods described in ref. [13] applied to IGAP’s previously reported ORs and confidence intervals to determine significance of subgroup associations compared to IGAP.

We used genetic data from all cognitively normal elderly controls and all people with Alzheimer’s disease to generate Alzheimer’s disease and subgroup genetic risk scores. We used (1) IGAP SNPs and effect sizes to generate Alzheimer’s disease risk scores and (2) our results to generate risk scores for each of the five subgroups.

To evaluate these risk scores, we used logistic regression with Alzheimer’s disease case vs. control status as the outcome, and included terms for age and sex. We compared a model with just the addition of the IGAP genetic risk score to a model that incorporated that score plus the five subgroup risk scores. Finally, we compared area under the receiver operator characteristic (ROC) curves for the model with the IGAP risk score to a model that did not include that term but included terms for the five subgroup risk scores. Further details are shown in Supplementary Text 12.

### Data sharing

Co-calibrated scores for each domain are available from the parent studies. GWAS meta-analysis summary statistics for each subgroup will be available on the National Institute on Aging Genetics of Alzheimer’s Disease Storage Site (NIAGADS; https://www.niagads.org/datasets/ng00073). Other study materials are available here: https://digital.lib.washington.edu/researchworks/handle/1773/40364.

### Role of the funding source

The funders of the study had no role in study design, data collection, data analysis, data interpretation, or writing of the report. All authors had full access to all the data in the study. The corresponding author had final responsibility for the decision to submit the publication.

## Results

In all, there were 4050 people with sufficient cognitive data to be classified into a subgroup. Demographic characteristics and average cognitive domain scores by study are shown in Table 1. Participants in the prospective cohort studies (ACT, MAP, and ROS) were older on average than those from ADNI and PITT. Most participants in each study self-reported white race (90% in ACT to 96% in MAP). There was some variation in cognitive performance across studies. The most notable differences from ACT (our reference for scaling) were for executive functioning in ADNI and ROS (average 0.8 units higher), and for language in MAP (average 0.8 units lower).

**Table 1:** Demographic and cognitive characteristics of people with late-onset Alzheimer’s disease by study and overall

Characteristic	ACT (*n* = 825)	ADNI (*n* = 650)	MAP (*n* = 404)	ROS (*n* = 419)	PITT (*n* = 1752)	Overall (*n* = 4050)
Female sex, *n* (%)	522 (63%)	267 (41%)	285 (71%)	302 (72%)	1106 (63%)	2482 (61%)
Age at diagnosis, mean (SD)	86 (6)	77 (7)	87 (6)	85 (6)	76 (7)	80 (8)
Years of education, mean (SD)	14 (3)	15 (3)	14 (3)	18 (3)	14 (3)	14 (3)
Self-reported white race, *n* (%)	743 (90%)	613 (94%)	386 (96%)	382 (91%)	1 595 (91%)	3 719 (92%)
*Composite scores for each cognitive domain, mean (SD)*						
Memory	0.0 (1.0)	0.0 (1.0)	−0.5 (1.1)	0.0 (1.0)	−0.6 (1.1)	-0.3 (1.1)
Visuospatial	0.0 (1.0)	0.3 (1.0)	0.1 (1.1)	0.1 (1.0)	0.4 (1.5)	0.3 (1.3)
Executive function	0.0 (1.0)	0.8 (1.2)	0.5 (1.0)	0.8 (1.0)	0.3 (0.8)	0.4 (1.0)
Language	0.0 (1.0)	0.5 (1.0)	−0.8 (1.2)	-0.4 (1.1)	−0.1 (1.2)	-0.1 (1.2)

**Fig. 1:**
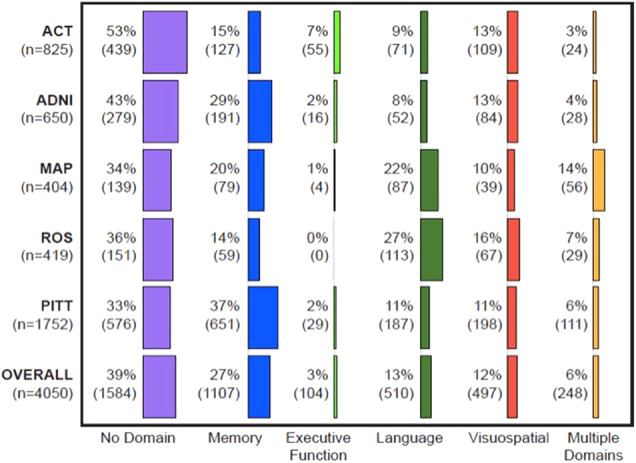
Proportions of people in each study and overall in each cognitively defined subgroup

Proportions of people in each subgroup are shown in Fig. 1. There was considerable heterogeneity in proportions across studies (*χ*^2^_df=20_ = 468.7, *p* = 1.0 × 10^−86^).

Demographic characteristics of people in each subgroup were similar to those for people with Alzheimer’s disease overall (Supplementary Table 29). The proportion who were female ranged from 51% for isolated substantial relative executive functioning impairment to 63% of those with isolated substantial relative visuospatial impairment. Mean age at diagnosis ranged from 79 for those with isolated substantial relative visuospatial or memory impairment to 82 for those with isolated substantial relative language impairment. Mean years of education did not vary substantially across subgroups.

There were 3701 people with *APOE* genotype data (Table 2). We published *APOE* results from ACT [4]; the proportion of those with isolated substantial memory impairment with ≥ 1 *APOE* ε4 allele was 12% higher than overall in that study. This finding was consistent across all five studies. Overall, the proportion of people with ≥ 1 *APOE* ε4 allele was 15% higher in those with isolated substantial memory impairment (65%) compared with the entire sample (50%). The differences in proportions with ≥ 1 *APOE* ε4 allele were highly significant (*p* = 1.5 × 10^−27^). The *APOE* result was not sensitive to choosing other thresholds to indicate a substantial relative impairment (Supplementary Text 4 and Supplementary Fig. 2).

**Table 2:** Proportion of those in each cognitively defined subgroup with ≥ 1 *APOE* ϵ4 allele, by study and overall

Study	No domain	Memory	Executive	Language	Visuospatial	Multiple domains	Overall	*p*-value^a^
ACT (*n* = 711)	134 (35%)	51 (46%)	11 (24%)	14 (24%)	27 (30%)	6 (32%)	243 (34%)	0.022
ADNI (*n* = 650)	185 (66%)	139 (73%)	10 (63%)	30 (58%)	48 (57%)	15 (54%)	427 (66%)	0.07
MAP (*n* = 386)	45 (34%)	33 (44%)	1 (33%)	23 (27%)	9 (26%)	21 (39%)	132 (34%)	0.24
ROS (*n* = 393)	49 (35%)	26 (48%)	–	31 (28%)	26 (41%)	11 (46%)	143 (36%)	0.08
PITT (*n* = 1561)	283 (54%)	411 (70%)	15 (65%)	72 (44%)	97 (57%)	39 (44%)	917 (59%)	5.1 × 10^−11^
Overall (*n* = 3701)	696 (48%)	660 (65%)	37 (42%)	170 (36%)	207 (47%)	92 (43%)	1861 (50%)	1.5 × 10^−27^

^a^*p* values based on *χ*^2^_(4)_ for ROS, otherwise *χ*^2^_(5)_

**Fig. 2:**
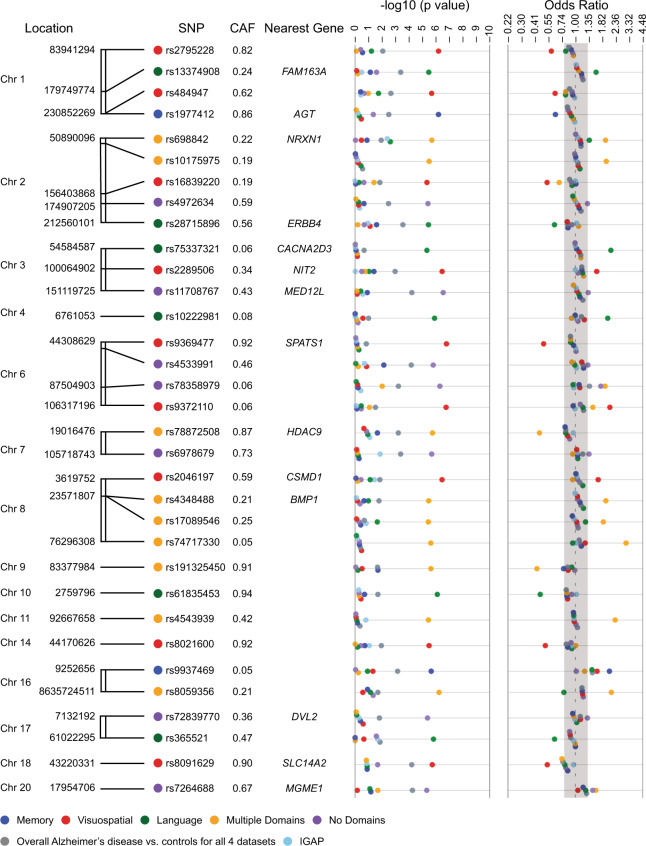
Novel SNPs associated with cognitively defined subdomains with *p* < 10^–5^ and OR < 0.77 or > 1.33. CAF=coded allele frequency. SNPs further than 50 kB from a gene do not have a gene name reported here. Gray shading in the odds ratios column of the figure delineates ORs > 0.77 and < 1.30, which is the range of ORs outside *APOE* from the International Genomics of Alzheimer’s Project (IGAP) [6]

There were 2431 people with late-onset Alzheimer’s disease and 3447 cognitively normal elderly controls with genome-wide SNP data. Top results are shown in Fig. 2. There were 33 loci outside Chromosome 19 where the *p*-value for one subgroup was < 5 × 10^−5^. All of these had ORs < 0.77 or > 1.30 compared to cognitively normal elderly controls. These included nine loci for those with isolated substantial visuospatial impairment (red dots, including rs2289506 near *NIT2* on chromosome 3, rs9369477 near *SPATS1* on chromosome 4, rs2046197 near *CSMD1* on chromosome 8, and rs8091629 near *SLC14A2* on chromosome 18), nine for those with multiple domains with substantial relative impairments (yellow dots, including rs698842 near *NRXN1* on chromosome 2, rs78872508 near *HDAC9* on chromosome 7, and rs4348488 near *BMP1* on chromosome 8), seven for those with no domain with a substantial relative impairment (purple dots, including rs11708767 near *MED12L* on chromosome 3, rs72839770 near *DVL2* on chromosome 17, and rs7264688 near *MGME1* on chromosome 20), six for those with isolated substantial language impairment (green dots, including rs13374908 near *FAM163A* on chromosome 1, rs28715896 near *ERBB4* on chromosome 2, and rs75337321 near *CACNA2D3* on chromosome 3), and two for those with isolated substantial memory impairment (blue dots, including rs1977412 near *AGT* on chromosome 1), and one for those with no domains with substantial impairments (purple dot, rs7264688 near *MGME1* on chromosome 20). Other loci shown in Fig. 2 not named in the preceding sentence were > 50 kb from genes. Replication results are in Supplementary Tables 2a and 2b. All ORs were in the same direction for all these SNPs except rs28715896 on chromosome 2 near *ERBB4*, rs61835453 on chromosome 10, and rs365521 on chromosome 17. Heterogeneity *p* values did not suggest heterogeneity for any of these SNPs. No SNP outside the *APOE* region reached *p* < 5 × 10^−8^, the traditional level of genome-wide significance (Fig. 2).

Results for selected IGAP loci are shown in Supplementary Table 30. We selected subgroups with meta-analytic ORs < 0.77 or > 1.30 and for which results from all four data sets were in the same direction. Results for other IGAP SNPs for all studies and all subgroups are in Supplementary Table 31.

Across all datasets, there were 5878 people with SNP data who were either normal controls or Alzheimer’s disease cases. The logistic regression model for case vs. control status with age, sex, and IGAP gene scores had a likelihood of 176.13 and a pseudo-*R*^2^ of 0.022. A model that included all of those terms and also included five subgroup gene scores had a likelihood of 311.94 and a pseudo-*R*^2^ of 0.039. These were nested models; we compared them with a likelihood ratio test with five degrees of freedom. The difference in likelihoods was highly significant (*p* = 1.39 × 10^−27^).

The area under the ROC curve for the model with age, sex, and the IGAP genetic risk score was 0.60 (95% CI 0.58, 0.61), while for the model with age, sex, and five subgroup genetic risk scores, the area under the ROC curve was 0.62 (95% CI 0.61, 0.64). This difference was statistically significant (*χ*^2^_df=1_ = 11.15, *p* = 0.0008). Further analyses are described in Supplementary Text 12.

## Discussion

We used modern psychometric methods to co-calibrate cognitive data to generate domain scores across five different studies of older adults with research-quality clinical Alzheimer’s disease diagnoses. We obtained scores on the same metric, so scores from different studies were directly comparable to each other. The proportion of people with Alzheimer’s disease in each study categorized in each subgroup varied across studies (Fig. 1). We used genetic data to determine whether our categorization scheme resulted in biologically coherent subgroups. Top genetic associations from each subgroup were consistent across studies, suggesting our findings were not due to idiosyncrasies of any particular study. Gene scores for subgroups performed better in predicting case vs. control status than gene scores derived from Alzheimer’s disease case-control analyses.

*APOE* genotype was significantly different across subgroups. We previously showed in ACT that more people with isolated substantial relative memory impairment had at least one *APOE* ε4 allele than people in other subgroups [4]. We robustly replicated that finding here (Table 2). Associations between *APOE* ε4 alleles and memory impairment among people with Alzheimer’s disease have been previously noted [14].

Outside of chromosome 19 which was dominated by *APOE*-related signals for all subgroups, we identified 33 loci with *p* < 10^−5^ for at least one subgroup. All of these had ORs < 0.77 or > 1.30 (Fig. 2). There were consistent findings across datasets for nearly all these loci (Supplementary Text 7–11).

Gene scores from IGAP SNPs explained a modest amount of risk for case-control status. Adding gene scores from our cognitively defined subgroups improved prediction of Alzheimer’s disease. Furthermore, in a head-to-head comparison, gene scores for cognitively defined subgroups did a better job predicting Alzheimer’s disease status than did gene scores from IGAP SNPs.

These data provide strong support for the biological coherence of subgroups produced by our categorization scheme. Each subgroup we analyzed has extreme ORs at novel SNPs that were consistent across multiple independent samples. Even with the relatively small sample sizes from these studies, the large effect sizes at common SNPs produced *p* values that are close to genome-wide significance.

Others have used different data sources to categorize people with Alzheimer’s disease. Sweet and colleagues compared people with Alzheimer’s disease who developed psychosis to those who did not [15]. They identified a few interesting loci, but effect sizes were much smaller than those reported here (Supplementary Text 13).

We were part of a consortium evaluating rates of decline among people with Alzheimer’s disease [16]. The evidence in support of rates of decline, as an organizing characteristic among people with Alzheimer’s disease, is not nearly as strong as that shown here for cognitively defined subgroups.

Others have used cluster analysis approaches applied to neuropsychological [17, 18] or imaging [19, 20] data to categorize people with Alzheimer’s disease. There are very important distinctions between those approaches and the approach adopted here. In cluster analysis, the computer maximizes some distance across groups in a way that may not make clinical or biological sense. Disease severity is an important consideration (see [21] for a better discussion).

Our approach began with theory and focused exclusively on cognitive data. An early paper considered differences between memory and executive functioning among people with Alzheimer’s disease [22]. Differences between these scores enables memory to serve as something of a proxy for disease severity. This framework is useful for considering dysexecutive Alzheimer’s disease [23–26].

We have extended that framework to incorporate additional cognitive domains. The field has increasingly emphasized the importance of Alzheimer’s disease variants including primary progressive aphasia (PPA) and posterior cortical atrophy (PCA) [27]; these rare subtypes are described as typically having early onset. Clinical descriptions of the cognitive patterns of these variants emphasize relative deficits between language (PPA) or visuospatial functioning (PCA) and other domains. We thus incorporate average performance across domains, and differences from that average, to more fully capture the range of clinical heterogeneity described in late onset Alzheimer’s disease [1].

Our investigation of cognitively defined late-onset Alzheimer’s disease was motivated by considerations of previously described Alzheimer’s disease variants, including logopenic PPA and PCA. Overlap between logopenic PPA and a history of dyslexia has led to the question of whether altered patterns of language network connectivity associated with dyslexia may predispose to selective regional vulnerability to Alzheimer’s pathological processes [28]. The work described here focuses exclusively on people with typical late-onset Alzheimer’s disease and demonstrates substantial numbers of people with patterns of relative cognitive impairment that are similar to those described in rare Alzheimer’s subtypes, such as logopenic PPA and PCA. These findings suggest the need to further evaluate relationships between cognitively defined late onset Alzheimer’s disease subgroups and these rare Alzheimer’s subtypes. For example, it is unknown whether rates of dyslexia are higher among people with isolated substantial relative language impairment than people with other subtypes of late-onset Alzheimer’s disease. Furthermore, it will be fascinating to see whether people with logopenic variant PPA may have genetic patterns similar to those of people with isolated substantial relative language impairment, and whether people with PCA may have genetic patterns similar to those of people with isolated substantial visuospatial impairment. It will be of great interest in the coming years to elucidate relationships across these conditions. Such investigations may reveal important insights into shared and distinct mechanisms underlying susceptibility to these conditions.

Our results should be considered mindful of limitations of our study. Data evaluated here are from studies with well-educated people of European ancestry. It will be important to replicate this approach among people with diverse genetic backgrounds. While we combined data from five large studies, the resulting subgroups were underpowered to reach genome-wide significance, and one subgroup (isolated substantial relative executive functioning impairment) was too small to analyze at all. It will be important to incorporate additional data sets to see whether novel suggestive loci reach genome-wide significance, and to identify additional loci. We used a large threshold of 0.80 SD to characterize “substantial” relative impairments, which may be too conservative. Our categorization approach relies exclusively on cognitive data. We could imagine a more optimal approach that also incorporates imaging and/or fluid biomarkers.

In conclusion, genome-wide genetic data enabled us to determine that a cognitively defined categorization scheme produced biologically coherent subgroups of people with Alzheimer’s disease. This is an important result on the road towards personalized medicine.

## Electronic supplementary material

Supplemental materials
