# Supplementary material for: Genetic data and cognitively defined late-onset Alzheimer’s disease subgroups
Source: Mol Psychiatry. 2018 Dec 4;25(11):2942–51. doi: 10.1038/s41380-018-0298-8 (PMC6548676; doi:10.1038/s41380-018-0298-8)
Supplement: Supplementary file 1 — Supplemental materials [file 41380_2018_298_MOESM1_ESM.docx]

**Genetic data and cognitively-defined late-onset Alzheimer’s disease subgroups**

Shubhabrata Mukherjee, PhD (1), Jesse Mez, MD MS (2), Emily Trittschuh, PhD (3, 4), Andrew J. Saykin, PsyD (5), Laura E. Gibbons, PhD (1), David W. Fardo, PhD (6), Madeline Wessels (7), Julianna Bauman (7), Mackenzie Moore (7), Seo-Eun Choi, PhD (1), Alden L. Gross, PhD MHS (8), Joanne Rich, MLIS (9), Diana K.N. Louden, MLib (9), R. Elizabeth Sanders, BA (1), Thomas J. Grabowski, MD (10, 11), Thomas Bird, MD (10), Susan M. McCurry, PhD (12), Beth E. Snitz, PhD (13), M. Ilyas Kamboh, PhD (14), Oscar L. Lopez, MD (13, 15), Philip L. De Jager, MD PhD (16), David A. Bennett, MD (17), C. Dirk Keene, MD PhD (18), Eric B. Larson, MD MPH (1, 19), for the EPAD Study Group and Investigators from ACT, ROS, MAP, ADNI**, and the University of Pittsburgh ADRC, and Paul K. Crane, MD MPH (1)

**Supplemental Materials**

**Table of Contents**

#### **Supplemental Text 1.** Psychometric analyses **6**

**1A.** Confirmatory factor analyses in each study **6**

**1B.** Co-calibration of the domains across ACT, ADNI, and ROS/MAP **7**

**1C.** Confirmatory factor analysis model considerations in co-calibration models **8**

#### **Supplemental Text 2.** Neuropsychological items by domain for each study and fit statistics from CFA models **9**

**Memory** **8**

Supplemental Table 1. Items and secondary structure for memory for the ACT study **9**

Supplemental Table 2. Items and secondary structure for memory for the ADNI study **10**

Supplemental Table 3. Items and secondary structure for memory for the ROS and MAP studies **11**

Supplemental Table 4. Co-calibration of memory across ACT, ADNI, ROS/MAP **12**

**Executive functioning** **14**

Supplemental Table 5. Items and secondary structure for executive functioning for the ACT study **14**

Supplemental Table 6. Items and secondary structure for executive functioning for the ADNI study **14**

Supplemental Table 7. Items and secondary structure for executive functioning for the ROS and MAP studies **15**

Supplemental Table 8. Co-calibration of executive functioning across ACT, ADNI, ROS/MAP **15**

**Language** **17**

Supplemental Table 9. Items and secondary structure for language for the ACT study **17**

Supplemental Table 10. Items and secondary structure for language for the ADNI study **17**

Supplemental Table 11. Items and secondary structure for language for the ROS and MAP studies **18**

Supplemental Table 12. Co-calibration of language across ACT, ADNI, ROS/MAP **19**

**Visuospatial functioning** **20**

Supplemental Table 13. Items and secondary structure for visuospatial functioning for the ACT study **20**

Supplemental Table 14. Items and secondary structure for visuospatial functioning for the ADNI study **20**

Supplemental Table 15. Items and secondary structure for visuospatial functioning for the ROS and MAP studies **20**

Supplemental Table 16. Co-calibration of visuospatial functioning across ACT, ADNI, ROS/MAP **21**

#### **Supplemental Text 3.** Choice of the threshold of 0.80 points **22**

**Supplemental Figure 1.** Cognitively defined subgroup membership at each threshold level **23**

#### **Supplemental Text 4.** Sensitivity of APOE findings to choice of threshold **24**

**Supplemental Figure 2.** Sensitivity analysis of *APOE* ε4 proportions across subgroups at thresholds ranging from -0.40 to -1.25 **24**

#### **Supplemental Text 5.** Addition of University of Pittsburgh study to the pipeline **25**

Supplemental Table 17. Memory specification for the PITT dataset **25**

Supplemental Table 18. Executive function specification for the PITT dataset **25**

Supplemental Table 19. Language specification for the PITT dataset **25**

Supplemental Table 20. Visuospatial functioning specification for the PITT dataset **26**

#### **Supplemental Text 6.** Genetic analyses **27**

**6A.** Cohorts **27**

**6B.** Imputation and SNP selection for GWAS analyses **28**

**6C.** Meta analysis **28**

**6D.** Q-Q plots from meta-analyses of GWAS results **29**

Supplemental Figure 3. Quantile-Quantile plots **29**

**6E.** Manhattan plots from meta-analyses of GWAS results **31**

Supplemental Figure 4. Manhattan plots from meta-analysis of GWAS results **31**

#### **6F.** Regional association plots of top hits from meta-analyses for each Alzheimer’s disease subtype **37**

Supplemental Figure 5. Regional association plots from meta-analysis for each Alzheimer’s disease subtype **37**

#### **Supplement Text 7. Genetic results: Memory SNPs** **54**

Supplemental Table 21a. Meta-analysis results for memory for memory SNPs **54**

Supplemental Table 21b. Study-specific results for memory SNPs **54**

Supplemental Table 21c.Meta-analysis for other subgroups for memory SNPs **54**

#### **Supplement Text 8. Genetic results: Visuospatial SNPs** **55**

Supplemental Table 22a. Meta-analysis results for visuospatial for visuospatial SNPs **55**

Supplemental Table 22b. Study-specific results for visuospatial SNPs **55**

Supplemental Table 22c.Meta-analysis for other subgroups for visuospatial SNPs **56**

#### **Supplement Text 9. Genetic results: Language SNPs** **57**

Supplemental Table 23a. Meta-analysis results for language for language SNPs **57**

Supplemental Table 23b. Study-specific results for language SNPs **57**

Supplemental Table 23c.Meta-analysis for other subgroups for language SNPs **57**

#### **Supplement Text 10. Genetic results: Multiple domains SNPs** **58**

Supplemental Table 24a. Meta-analysis results for multiple domains group for multiple domains SNPs **58**

Supplemental Table 24b. Study-specific results for multiple domains SNPs **58**

Supplemental Table 24c.Meta-analysis for other subgroups for multiple domains SNPs **59**

#### **Supplement Text 11. Genetic results: No domain with a substantial relative impairment SNPs** **60**

Supplemental Table 25a. Meta-analysis results for no domain with substantial relative impairment SNPs **60**

Supplemental Table 25b. Study-specific results for no domain with a substantial relative impairment SNPs **60**

Supplemental Table 25c.Meta-analysis for other subgroups for no domain with a substantial relative impairment SNPs **61**

#### **Supplemental Text 12.** Generation of genetic risk scores and their ability to predict Alzheimer’s disease case/control status **62**

Supplemental Table 26.Area under the receiver operator characteristic curve for predicting late-onset Alzheimer’s disease case-control status with different groups of SNPs **63**

Supplemental Table 27. Tests of equality of receiver operator characteristic curves compared with that produced from IGAP SNPs (compared with Model A) **63**

#### Supplemental Table 28. SNPs used to calculate gene scores **64**

#### **Supplemental Text 13.** Information regarding the Sweet et al. analyses **67**

#### **Supplemental Table 29.** Demographic and cognitive characteristics by subgroup **68**

#### **Supplemental Table 30.** IGAP SNPs with OR>1.30 or <0.77 in one subgroup and for which results from all four datasets were in the same direction **69**

#### **Supplemental Table 31.** Data for IGAP SNPs for all the studies and all the subgroups **70**

#### **References** **74**

# Supplemental Text 1. Psychometric analyses

**Supplemental Text 1A. Confirmatory factor analyses in each study**

**Step 1: Domain assignment:** In each of the studies (Adult Changes in Thought [ACT], Alzheimer’s Disease Neuroimaging Initiative [ADNI], the Religious Orders Study–Memory and Aging Project (ROS/MAP], and the University of Pittsburgh data set [PITT]), the expert panel (Dr. Trittschuh, Dr. Mez, and Dr. Saykin) assigned items from the neuropsychological battery to one of the four domains (memory, language, executive functioning, and visuospatial ability); other items did not map to any of these domains. The expert panel also assigned each of these items to sub-domains based on the cognitive processes involved in each task. We also noted methods effects where the same stimulus was used in multiple assessments. We also used a data-driven approach looking at patterns of responses among participants to identify alternate possible secondary domain structures.

**Step 2: Data quality control:** Each of the studies sent their neuropsychological data sets to our team and Ms. Sanders, our data manager, ran an initial quality control on these. Ms. Sanders prepared a data set which included item-level data for individuals at their first Alzheimer’s disease diagnosis. Before running psychometric models, we performed additional recoding of the data. Some items such as Trails A and B were reverse coded. We checked each item to make sure lower values represent lower cognitive performance. We considered the distribution of each item among those with non-missing data and combined categories as needed. Our goals were a.) to avoid sparse categories (operationally defined as <5 responses for each study administering each item) and b.) to have a maximum of 10 categories, which is the maximum number of categories handled by Mplus v7.4^1^. We treated each item as an ordinal indicator of the domain—the numerical value assigned to each category is irrelevant beyond its rank, e.g. calling the lowest category 3 points vs. 18 points makes no difference in how the item is treated or what the final score would be.

We also looked at informative missingness in each study and recoded relevant items accordingly. For example, some of the studies include multiple missing codes, where it was possible to identify refusal to respond to an item as opposed to the interviewer ran out of time and the item was never administered. The first of these—refusal—we took as informative missing and assigned that code to the lowest response category, while the second of these—missing due to scheduling etc.—we took as non-informative missing and omitted that item from consideration.

**Step 3: Confirmatory factor analyses:** We then turned to confirmatory factor analysis modeling with Mplus^2^ using a Robust Weighted Least Squares including terms for the mean and the variance (WLSMV) estimator. We ran four models: a.) a single factor model, with no residual structure; b.) A theory-driven cognitive process bifactor model, using the a priori sub-domain assignments; c.) a theory-driven methods effects bifactor model, using the “methods effects” assignments; and d.) a data-driven bifactor model, using hierarchical clustering-assigned sub-domains. We consulted the expert panel on the sub-domain assignment of items in our data-driven approach to make sure these models made sense to our experts. Our overall strategy was that we would choose the single factor model if adding secondary factors did not markedly improve model fit and if adding secondary factors did not markedly impact any individual’s score (see below).

Our goal with the three bifactor models (models b, c, and d) was to identify a single candidate bifactor model to compare with the single-factor model (model a). Our criteria for selecting the candidate bifactor model included fit statistics (see below) and concordance of model results with theory, such as all loadings on secondary factors being positive. The fit statistics we considered were the confirmatory fit index (CFI) where higher values indicate better fit; thresholds of 0.90 and 0.95 have been used in other settings as criteria for adequate or good fit^3, 4^; the Tucker-Lewis Index (TLI), which has similar criteria as the CFI; and the root mean squared error of approximation (RMSEA), where lower values indicate better fit, and thresholds of 0.08 and 0.05 have been used in other settings as criteria for adequate or good fit^3, 4^.

When comparing the single factor model with the best bifactor model, we a) looked at whether loadings on the primary factor were within 10% of each other across the two models and b) compared the scores for the single factor model vs. scores for the final candidate bifactor model. We used as our threshold a difference of 0.30 units. We chose this value based on the default stopping rule for computerized adaptive testing; this has been used for years as the default level of tolerable measurement differences in the setting of computerized adaptive tests. While arbitrary, this is a level of ambiguity that has been thought to be tolerable in a variety of situations. If there were a substantial number of people for whom the differences in scores were larger than 0.3 from each other, and if the bifactor model conformed to our theory better and had better fit statistics, we selected the final candidate bifactor model as our choice for modeling a domain.

# Supplemental Text 1B. Co-calibration of the domains across ACT, ADNI, and ROS/MAP

**Step 1: Identification of anchor items:** Co-calibration requires either the same people taking different tests or different tests sharing common items. Here we had common items. We identified candidate anchor items with identical content across tests administered in different studies and ensured that their relationship with the underlying ability tested was the same across studies by performing preliminary confirmatory factor analysis models within each study. These items were then used to anchor the scales in each domain to a common metric**.** We consulted the expert panel (Dr. Trittschuh) to make sure we chose the anchor items correctly.

**Step 2: Quality control for anchor items:** Anchor items were cleaned and recoded after merging in the items from all the studies making sure that the range of the anchor items were similar in each study. We carefully reviewed documentation from each study to ensure that the stimulus was precisely the same, that the response options were precisely the same or could be re-coded to be the same, and that we were mapping data from each study in a way that the same response would result in the same score regardless of which study the person was enrolled in.

A note regarding response options—in many cases the stimulus is fairly open-ended, such as “can you please draw from memory the figure you copied a while ago”, where the participant is handed a blank sheet of paper and a writing implement. The resulting drawing then gets scored based on how similar it was to the initial stimulus figure. The specific scoring applied to such a stimulus could vary across studies. One study could score such an item as correct vs. incorrect, while another could apply points for various aspects of the drawing. We reviewed the scoring documentation from both studies to determine what “correct” meant in the first study, and how many aspects of the drawing would need to be present for a “correct” score in that study. Then we would map all scores from the second study that would have resulted in a “correct” score in the first study to a “correct” score, and all other scores from the second study to an “incorrect” score. In this way, the resulting score is invariant to which study the person is participating in, as each response would be consistently scored regardless of study.

**Step 3: Confirmatory factor analyses:** We co-calibrated each of the four domains (memory, executive functioning, language, and visuospatial ability) by incorporating the components of the best model in each study (i.e., the final single-factor or bifactor model selected as described above) into one mega-calibration model.

One particularly tricky aspect of co-calibrating scores using bifactor models is how to handle secondary domains. Some anchor items had loadings on the primary domain (e.g. memory) and also on a secondary domain. That structure by itself does not lead to conceptual problems. However, item representation of the secondary domain may vary across studies, with variable numbers of items, and potential missing data and identifiability issues. To address this we used robust maximum likelihood (MLR) estimation that is robust to missing data, and assigned all subdomain indicators across studies to the same subdomain. Unlike running a CFA model with the WLSMV estimator, a CFA model with MLR estimator does not output fit statistics like CFI/TLI/RMSEA. For our purposes, these secondary domains were nuisances. We performed a number of sensitivity analyses to reassure ourselves that scores on the primary domain were minimally impacted by various ways of specifying the mean and variance on secondary domains. In the final models we selected, we specified a mean of 0 and a variance of 1 for each secondary domain factor, regardless of the number of studies that included items that loaded on that factor.

Once we had fit the final mega-calibration model for each domain, we extracted factor scores for the primary factor (e.g. memory). The resulting scores are on the same metric with a mean of 0 and variance of 1. We used all participants with relevant data to fit data for each domain, so different the scale for each domain was based on models that included different specific people, since some people were missing for some domains. We therefore picked a reference population for standardizing scores for each domain. We used ACT for this, as it was a community-based prospective cohort study, and had a very large sample (n=825) of people with sufficient cognitive data to generate all of our scores. We applied the same standardization to all participants for each study.

Thus, a score of 0, regardless of study, reflects the mean for people with Alzheimer’s disease in the ACT study; and a score of -1, regardless of study, reflects 1 SD below the mean for people with Alzheimer’s disease in the ACT study.

For future data sets in our pipeline such as University of Pittsburgh (PITT), we used these estimated thresholds and loadings of items from the co-calibration mega-calibration models to obtain scores for individuals. New items (not part of ACT, ADNI, and ROS/MAP) were freely estimated while already seen items will have their parameters fixed based on these mega-calibration models.

# Supplemental Text 1C. Confirmatory factor analysis model considerations in co-calibration models

**1.** For all CFA models, we categorized items to ≤ 10 categories. For co-calibration purpose, we had to re-categorize some of the items even though they already had ≤ 10 categories. This was because some studies had more granular data (more categories) for anchor items compared to other studies. In these cases, after we estimated item parameters from the co-calibration model, we re-estimated parameters of the anchor item(s) in the most granular form in the given study. For example, the item “q20mme” was an anchor item for visuospatial ability administered in ACT, ROS/MAP, and ADNI. The item asks individuals to copy intersecting pentagons. In ROS/MAP and ADNI, this item is coded as 0/1 (incorrect/correct) while in ACT it is coded 0–10 (four points for aspects of the left pentagon, four points for aspects of the right pentagon, two points for aspects of the intersection). For co-calibration purpose, we dichotomized this item to 0/1. We consulted scoring algorithms for each of the studies to determine that only scores of 10/10 from the ACT study would have received scores of 1 from ROS/MAP or ADNI; any drawing receiving a score of 9 or fewer from ACT would have scored a 0 in the other studies.

After using re-coded items for co-calibration, we fixed all of the other items to their values from the co-calibration run and freely estimated parameters for re-coded anchors in their most granular form. This approach enabled us to obtain more precise scores in studies that incorporated more granular scoring rules, while still using all items administered across studies to co-calibrate metrics across studies.

**2.** The base co-calibration exercise for each of the four domains was performed across ACT, ROS/MAP, and ADNI. PITT data were subsequently added with the following steps. For each domain, we identified anchor items and fixed their item parameters to those estimated previously in the base co-calibration models; unique items administered in the new study that were not administered to people in ACT, ROS/MAP, or ADNI were freely estimated.

In these models,

a) The mean and variance for the primary factor were freely estimated.

b) If every item in a sub-domain in the new data had parameters available from the co-calibration model, we fixed those item parameters to their previously identified values, and allowed the mean and variance to be freely estimated in the new data.

If no item from a sub-domain had parameters available, then we freely estimated each of the sub-domain loadings, fixing the mean and variance of the subdomain factor to 0 and 1.

If there was a mix of previously specified and new items in a subdomain, we fixed the parameters for the previously specified items, and allowed the mean and variance of the factor and the loadings for new items to be freely estimated in the new data.

**NOTE:** Detailed overview and all code snippets can be obtained from authors on request.

# Supplemental Text 2. Neuropsychological items by domain for each study and fit statistics from CFA models

## MEMORY

### ACT: Final model was a theory driven methods-effects bifactor model with CFI = 0.923, TLI = 0.914, and RMSEA = 0.052. The following items were included in the CFA analysis (Supplemental Table 1).

**Supplemental Table 1. Items and secondary structure for memory for the ACT study**

| **Study** | **Variable** | **Description** | **Secondary Structure** |
| --- | --- | --- | --- |
| ACT | mat_mem | Mattis Dementia Rating Scale Memory score |  |
| ACT | w_in_c1 | Word list learning trial 1 total score | F1 |
| ACT | w_in_c2 | Word list learning trial 2 total score | F1 |
| ACT | w_in_c3 | Word list learning trial 3 total score | F1 |
| ACT | w_rcl_c | Word List Recall—correct | F1 |
| ACT | w_rcg_t | Word Recognition—total correct | F1 |
| ACT | cp_re_ci | Constructional Praxis Delay—circle | F2 |
| ACT | cp_re_di | Constructional Praxis Delay—diamond | F2 |
| ACT | cp_re_re | Constructional Praxis Delay—rectangles | F2 |
| ACT | cp_re_cu | Constructional Praxis Delay—cube | F2 |
| ACT | w_lm_ima | Logical Mem I—immediate recall total story A | F3 |
| ACT | w_lm_imb | Logical Mem I—immediate recall total story B | F4 |
| ACT | w_lm_dea | Logical Mem II—delayed recall total story A | F3 |
| ACT | w_lm_deb | Logical Mem II—delayed recall total story B | F4 |
| ACT | w_vp_ine | Verbal Paired Associates I easy | F5 |
| ACT | w_vp_inh | Verbal Paired Associates I hard | F6 |
| ACT | w_vp_ree | Verbal Paired Associates II easy | F5 |
| ACT | w_vp_reh | Verbal Paired Associates II hard | F6 |
| ACT-CASI | rgs1 | repeat words | F7 |
| ACT-CASI | rc1a | Word recall—something to wear—1 | F7 |
| ACT-CASI | rc1b | Word recall—a color—1 | F7 |
| ACT-CASI | rc1c | Word recall—personal quality—1 |  |
| ACT-CASI | yr | What is today’s date?—year | F8 |
| ACT-CASI | mo | What is today’s date?—month | F8 |
| ACT-CASI | casi_dat | What is today’s date?—day | F8 |
| ACT-CASI | day | What day of week? | F8 |
| ACT-CASI | casi_ssn | What season is it? | F8 |
| ACT-CASI | spa | What state and city? | F8 |
| ACT-CASI | spb | What is this place? | F8 |
| ACT-CASI | rc2a | Word recall—something to wear—2 | F7 |
| ACT-CASI | rc2b | Word recall—a color—2 | F7 |
| ACT-CASI | rc2c | Word recall—personal quality—2 | F7 |
| ACT-CASI | rcobj | Recall of 5 objects |  |

### ADNI: Final model was a data driven bifactor model with CFI = 0.951, TLI = 0.946, and RMSEA = 0.036. The following items were included in the CFA analysis (Supplemental Table 2).

**Supplemental Table 2. Items and secondary structure for memory for the ADNI study**

| **Study** | **Variable** | **Description** | **Secondary Structure** |
| --- | --- | --- | --- |
| ADNI | limmtotal | Logical Memory—Immediate Recall | F1 |
| ADNI | ldeltotal | Logical Memory—Delayed Recall | F1 |
| ADNI | avtot1* | AVLT Trial 1 Total | F2 |
| ADNI | avtot2* | Trial 2 Total | F2 |
| ADNI | avtot3* | Trial 3 Total | F2 |
| ADNI | avtot4* | Trial 4 Total | F2 |
| ADNI | avtot5* | Trial 5 Total | F2 |
| ADNI | avtot6* | Trial 6 Total | F3 |
| ADNI | avtotb* | List B Total | F2 |
| ADNI | avdel30min* | 30 Minute Delay Total | F3 |
| ADNI | avdeltot* | Recognition Score | F4 |
| ADNI | q1score | ADAS Word Recall—score | F2 |
| ADNI | q4score | ADAS Delayed Word Recall | F4 |
| ADNI | q7score | ADAS Orientation—score | F5 |
| ADNI | q8score | ADAS Word Recognition—score |  |
| ADNI | mmdate | What is today's date? | F5 |
| ADNI | mmyear | What is the year? | F5 |
| ADNI | mmmonth | What is the month? | F5 |
| ADNI | mmday | What day of the week is today? | F5 |
| ADNI | mmseason | What season is it? |  |
| ADNI | mmhospit | What is the name of this hospital (clinic, place)? |  |
| ADNI | mmfloor | What floor are we on? |  |
| ADNI | mmcity | What town or city are we in? |  |
| ADNI | mmarea | What county (district, borough, area) are we in? |  |
| ADNI | mmstate | What state are we in? |  |
| ADNI | mmball | Ball | F6 |
| ADNI | mmflag | Flag | F6 |
| ADNI | mmtree | Tree | F6 |
| ADNI | mmballdl | Ball delayed | F7 |
| ADNI | mmflagdl | Flag delayed | F7 |
| ADNI | mmtreedl | Tree delayed | F7 |
| ADNI | imm1sum | Immediate recall of the MoCA list (#1) | F2 |
| ADNI | imm2sum | Immediate recall of the MoCA list(#2) | F2 |
| ADNI | delsum | Delayed recall of the MoCA list |  |

MoCA (blue) items were only administered in ADNI GO/2 while orange items were in all ADNI waves (1/GO/2).

ADNI administered two versions (different word lists) of RAVLT (avtot1–avdeltot) and three different versions of ADAS-Cog items (q*) across waves. We ran the model separately for the two versions. The ADAS-Cog versions were found to be equivalent while the RAVLT versions were not. For determining secondary factor structures and extracting model fit statistics, we considered all RAVLT versions to be equivalent. The different versions of RAVLT were taken into account in the final co-calibration phase.

There were additional MoCA items, which were the same (theoretically) as corresponding items from the Mini-Mental State Examination (MMSE). We excluded MoCA items if those items were already asked as part of the neuropsychological battery.

### ROS/MAP: Final model was a data driven bifactor model with CFI = 0.941, TLI = 0.929, and RMSEA = 0.063. The following items were included in the CFA analysis (Supplemental Table 3):

**Supplemental Table 3. Items and secondary structure for memory for the ROS and MAP studies**

| **Study** | **Variable** | **Description** | **Comments** | **Secondary Structure** |
| --- | --- | --- | --- | --- |
| ROS/MAP | Q1mme | What is the year? |  | F1 |
| ROS/MAP | Q2mme | What is the season of the year? |  |  |
| ROS/MAP | Q3mme | What is the date? |  | F1 |
| ROS/MAP | Q4mme | What is the day of the week? |  | F1 |
| ROS/MAP | Q5mme | What is the month? |  | F1 |
| ROS/MAP | Q6mme | What state are we in? |  | F2 |
| ROS/MAP | Q8mme | What city are we in? |  | F2 |
| ROS/MAP | Q7mme | What county are we in? |  | F2 |
| ROS/MAP | Q9mme | What room are we in? |  | F2 |
| ROS/MAP | Q10amme | What is the address of this place? |  | F2 |
| ROS/MAP | Q10bmme | Street Name |  | F2 |
| ROS/MAP | atb1 | Apple, table, penny (immediate) | 3 items collapsed |  |
| ROS/MAP | story | Logical memory |  | F3 |
| ROS/MAP | WordT1 | Word list learning Trial 1 | 10 items collapsed | F4 |
| ROS/MAP | WordT2 | Word list learning Trial 2 | “ | F4 |
| ROS/MAP | WordT3 | Word list learning Trial 3 | “ | F4 |
| ROS/MAP | WordRec | Which one of these words is from that list? (Word list recognition) | 10 items collapsed | F6 |
| ROS/MAP | Recall | Word list recall | 10 items collapsed | F6 |
| ROS/MAP | ebmt | East Boston immediate recall | 12 items collapsed | F5 |
| ROS/MAP | atb2 | apple, table, penny (delayed) | 3 items collapsed |  |
| ROS/MAP | ebdr | East Boston delayed recall | 12 items collapsed | F5 |
| ROS/MAP | Delay | Tell me the story again |  | F3 |

### Supplemental Table 4. Co-calibration of memory across ACT, ADNI, ROS/MAP

| **Study** | **Variable** | **Secondary structure** | **Comments** |
| --- | --- | --- | --- |
| ACT, ADNI, ROS/MAP | mmyear | F2 | Q1mme in ROS/MAP; yr in ACT |
| ACT, ADNI, ROS/MAP | mmseason |  | Q2mme in ROS/MAP; casi_ssn in ACT |
| ACT, ADNI, ROS/MAP | mmdate | F2 | Q3mme in ROS/MAP; casi_dat in ACT |
| ACT, ADNI, ROS/MAP | mmday | F2 | Q4mme in ROS/MAP; day in ACT |
| ACT, ADNI, ROS/MAP | mmmonth | F2 | Q5mme in ROS/MAP; mo in ACT |
| ACT, ADNI, ROS/MAP | mmctst | F6 | Collapsed (Q6mme Q8mme) in ROSMAP and (mmcity mmstate) in ADNI to create a single variable; spa in ACT |
| ACT, ADNI | limmtotal | F3 | limmtotal in ADNI; w_lm_ima in ACT |
| ACT, ADNI | ldeltotal | F3 | limmtotal in ADNI; w_lm_dea in ACT |
| ROS/MAP | Q7mme | F6 |  |
| ROS/MAP | Q9mme | F6 |  |
| ROS/MAP | Q10amme | F6 |  |
| ROS/MAP | Q10bmme | F6 |  |
| ROS/MAP | atb1 |  |  |
| ROS/MAP | story | F9 |  |
| ROS/MAP | WordT1 | F7 |  |
| ROS/MAP | WordT2 | F7 |  |
| ROS/MAP | WordT3 | F7 |  |
| ROS/MAP | ebmt | F8 |  |
| ROS/MAP | WordRec | F10 |  |
| ROS/MAP | atb2 |  |  |
| ROS/MAP | Recall | F10 |  |
| ROS/MAP | ebdr | F8 |  |
| ROS/MAP | Delay | F9 |  |
| ACT | mat_mem |  |  |
| ACT | w_in_c1 | F11 |  |
| ACT | w_in_c2 | F11 |  |
| ACT | w_in_c3 | F11 |  |
| ACT | w_rcl_c | F11 |  |
| ACT | w_rcg_t | F11 |  |
| ACT | cp_re_ci | F12 |  |
| ACT | cp_re_di | F12 |  |
| ACT | cp_re_re | F12 |  |
| ACT | cp_re_cu | F12 |  |
| ACT | w_lm_imb | F14 |  |
| ACT | w_lm_deb | F14 |  |
| ACT | w_vp_ine | F15 |  |
| ACT | w_vp_inh | F16 |  |
| ACT | w_vp_ree | F15 |  |
| ACT | w_vp_reh | F16 |  |
| ACT-CASI | rgs1 |  |  |
| ACT-CASI | rc1a | F13 |  |
| ACT-CASI | rc1b | F13 |  |
| ACT-CASI | rc1c | F13 |  |
| ACT-CASI | spb | F6 |  |
| ACT-CASI | rc2a | F13 |  |
| ACT-CASI | rc2b | F13 |  |
| ACT-CASI | rc2c | F13 |  |
| ACT-CASI | rcobj |  |  |
| ADNI | avtot1 | F1 | Each RAVLT item was split into two items to account for two versions of RAVLT used in ADNI at specific waves where both versions of the same item were loaded into the same secondary structure |
| ADNI | avtot2 | F1 |  |
| ADNI | avtot3 | F1 |  |
| ADNI | avtot4 | F1 |  |
| ADNI | avtot5 | F1 |  |
| ADNI | avtot6 | F5 |  |
| ADNI | avtotb | F1 |  |
| ADNI | avdel30min | F5 |  |
| ADNI | avdeltot | F4 |  |
| ADNI | q1score | F1 |  |
| ADNI | q4score | F4 |  |
| ADNI | q7score | F2 |  |
| ADNI | q8score |  |  |
| ADNI | mmhospit |  |  |
| ADNI | mmfloor |  |  |
| ADNI | mmarea |  |  |
| ADNI | bft1 |  | Immediate—ball, flag, tree collapsed |
| ADNI | bft2 |  | Delayed—ball, flag, tree collapsed |
| ADNI | imm1sum | F1 |  |
| ADNI | Imm2sum | F1 |  |
| ADNI | delsum |  |  |

## Executive functioning

### ACT: Final model was a data driven bifactor model with CFI = 0.948, TLI = 0.929, and RMSEA = 0.064. The following items were included in the CFA analysis (Supplemental Table 5):

**Supplemental Table 5. Items and secondary structure for executive functioning for the ACT study**

| **Study** | **Variable** | **Description** | **Comments** | **Secondary Structure** |
| --- | --- | --- | --- | --- |
| ACT | mat_attn | Mattis Dementia Rating Scale, Attention score |  |  |
| ACT | mat_conc | Mattis Dementia Rating Scale, Concentration score |  |  |
| ACT | mat_ip | Mattis Dementia Rating Scale, initiation / perseveration score |  |  |
| ACT | tr_a_tm | Trails A |  | F1 |
| ACT | tr_b_tm | Trails B |  | F1 |
| ACT | clockdr | Clock |  |  |
| ACT-CASI | dbsum | repeat numbers backward 1–3 | Repeat numbers backward—3 trials collapsed | F2 |
| ACT-CASI | subtra | Subtraction 1–3 | Subtraction—3 trials collapsed | F2 |
| ACT-CASI | sim | similarities |  |  |
| ACT-CASI | jgmt | judgement |  |  |

### ADNI: Final model was a theory driven methods-effects bifactor model with CFI = 0.951, TLI = 0.946, and RMSEA = 0.041. The following items were included in the CFA analysis (Supplemental Table 6):

**Supplemental Table 6. Items and secondary structure for executive functioning for the ADNI study**

| **Study** | **Variable** | **Description** | **Comments** | **Secondary Structure** |
| --- | --- | --- | --- | --- |
| ADNI | clockcirc | Approximately circular face |  |  |
| ADNI | clocksym | Symmetry of number placement |  | F2 |
| ADNI | clocknum | Correctness of numbers |  | F2 |
| ADNI | clockhand | Presence of the two hands |  |  |
| ADNI | clocktime | Presence of the two hands, set to ten after eleven |  |  |
| ADNI | dspanbac | Backward Total Correct |  | F4 |
| ADNI | traascor | Part A Time to Complete |  | F3 |
| ADNI | trabscor | Part B Time to complete |  | F3 |
| ADNI | digitscor | Digit Symbol Total Correct |  | F1 |
| ADNI | dspanfor | Digit Span Forward Total Correct |  | F4 |
| ADNI | q13score | Number cancellation task |  | F1 |
| ADNI | absmeas | Abstraction: watch-ruler |  |  |
| ADNI | abstran | Abstraction: train-bicycle |  |  |
| ADNI | trails | MoCA Trails |  |  |
| ADNI | digback | Digits Backward | 5 trials collapsed |  |
| ADNI | serial | Serial 7 total |  |  |
| ADNI | digfor | Digits Forward |  |  |
| ADNI | letters | List of Letters/Tapping: # Errors |  |  |

### ROS/MAP: Final model was a theory driven methods-effects model with CFI = 0.975, TLI = 0.960, and RMSEA = 0.064. The following items were included in the CFA analysis (Supplemental Table 7):

**Supplemental Table 7. Items and secondary structure for memory for the ROS and MAP studies**

| **Study** | **Variable** | **Description** | **Comments** | **Secondary structure** |
| --- | --- | --- | --- | --- |
| ROS/MAP | AA | Which piece would complete the pattern… | 4 A patterns merged |  |
| ROS/MAP | BB | Which piece would complete the pattern… | 8 B patterns merged |  |
| ROS/MAP | Q12bmme | Spell WORLD backwards |  |  |
| ROS/MAP | DigBak | digits backward | combined 12 items |  |
| ROS/MAP | cts_sdmt | symbol digits modality (oral) |  | F1 |
| ROS/MAP | cts_nccrtd | Number comparison |  | F1 |
| ROS/MAP | DigFor | digits forward | combined 12 items |  |

# Supplemental Table 8. Co-calibration of executive functioning across ACT, ADNI, ROS/MAP

| **Study** | **Variable** | **Description** | **Secondary structure** |
| --- | --- | --- | --- |
| ACT, ADNI | traascor | Trails A | F3 |
| ACT, ADNI | trabscor | Trails B | F3 |
| ADNI, ROS/MAP | dspanfor | Digit Span Forward: Total Correct | F4 |
| ADNI, ROS/MAP | dspanbac | Digit Span Backward: Total Correct | F4 |
| ROS/MAP | AA | Which piece would complete the pattern… |  |
| ROS/MAP | BB | Which piece would complete the pattern… |  |
| ROS/MAP | Q12bmme | Spell WORLD backwards |  |
| ROS/MAP | cts_sdmt | symbol digits modality (oral) | F6 |
| ROS/MAP | cts_nccrtd | Number comparison | F6 |
| ACT | mat_attn | Mattis Dementia Rating Scale |  |
| ACT | mat_conc | Mattis Dementia Rating Scale |  |
| ACT | mat_ip | Mattis Dementia Rating Scale |  |
| ACT | clockdr | Clock |  |
| ACT-CASI | dbsum | repeat numbers backward | F5 |
| ACT-CASI | subtra | subtraction | F5 |
| ACT-CASI | sim | similar types |  |
| ACT-CASI | jgmt | judgement |  |
| ADNI | clockcirc | Approximately circular face |  |
| ADNI | clocksym | Symmetry of number placement | F2 |
| ADNI | clocknum | Correctness of numbers | F2 |
| ADNI | clockhand | Presence of the two hands |  |
| ADNI | clocktime | Presence of the two hands, set to 10 after 11 |  |
| ADNI | digitscor | Digit Symbol Total Correct | F1 |
| ADNI | q13score | Number cancellation task | F1 |
| ADNI | absmeas | Abstraction: watch–ruler |  |
| ADNI | abstran | Abstraction: train–bicycle |  |
| ADNI | trails | Trails |  |
| ADNI | digback | Digits Backward |  |
| ADNI | serial | Serial 7 |  |
| ADNI | digfor | Digits Forward |  |
| ADNI | letters | List of Letters/Tapping: # Errors |  |

## Language

### ACT: Final model was a data driven bifactor model with CFI = 0.956, TLI = 0.943, and RMSEA = 0.055. The following items were included in the CFA analysis (Supplemental Table 9):

**Supplemental Table 9. Items and secondary structure for language for the ACT study**

| **Study** | **Variable** | **Description** | **Secondary Structure** |
| --- | --- | --- | --- |
| ACT | bnt_adpr* | Boston Naming Test ‒ 10-item version | F1 |
| ACT | bnt_cer * | Boston Naming Test ‒ 15-item version | F1 |
| ACT | v_flu_t | Verbal Fluency |  |
| ACT-CASI | animal | animals with 4 legs |  |
| ACT-CASI | rpta | repeat phrase 1 | F2 |
| ACT-CASI | rptb | repeat phrase 2 | F2 |
| ACT-CASI | cas_read | read and follow a command |  |
| ACT-CASI | cas_writ | write a sentence |  |
| ACT-CASI | cmd | obey oral commands |  |
| ACT-CASI | body | identify parts of body |  |
| ACT-CASI | obja | identify objects—1 | F3 |
| ACT-CASI | objb | identify objects—2 | F3 |

### * ACT administers all 15 items from the CERAD version of the Boston Naming Test (bnt_cer) and another 8 distinct items from a long version of the Boston Naming Test (bnt_adpr).

### ADNI: Final model was a theory driven methods-effects bifactor model with CFI = 0.951, TLI = 0.946, and RMSEA = 0.041. The following items were included in the CFA analysis (Supplemental Table 10):

**Supplemental Table 10. Items and secondary structure for language for the ADNI study**

| **Study** | **Variable** | **Description** | **Secondary structure** |
| --- | --- | --- | --- |
| ADNI | catanimsc | Category Fluency (Animals) —Total Correct | F1 |
| ADNI | catvegesc | Category Fluency (VegSupplemental Tables) —Total Correct |  |
| ADNI | bnttotal | Total Number Correct (1+3) | F1 |
| ADNI | q2score | ADAS Commands |  |
| ADNI | q5score | ADAS Naming | F1 |
| ADNI | q6score | Ideational Praxis—score |  |
| ADNI | mmwatch | Show wrist watch, ask: What is this? |  |
| ADNI | mmpencil | Show pencil, ask: What is this? |  |
| ADNI | mmrepeat | Say: Repeat after me: no ifs, ands, or buts. |  |
| ADNI | mmhand | Takes paper in right hand |  |
| ADNI | mmfold | Folds paper in half |  |
| ADNI | mmonflr | Puts paper on floor |  |
| ADNI | mmread | Present the piece of paper which reads—CLOSE YOUR EYES—and say: Read this and |  |
| ADNI | mmwrite | Give the participant a blank piece of paper and say: Write a sentence. |  |
| ADNI | camel | Camel |  |
| ADNI | lion | Lion |  |
| ADNI | rhino | Rhinoceros |  |
| ADNI | repeat1 | Repeat Sentence. |  |
| ADNI | repeat2 | Repeat Sentence. |  |
| ADNI | ffluency | Letter Fluency—F: Total number of correct words |  |

### ROS/MAP: Final model was a data driven model with CFI = 0.932, TLI = 0.924, and RMSEA = 0.036. The following items were included in the CFA analysis (Supplemental Table 11):

**Supplemental Table 11. Items and secondary structure for language for the ROS and MAP studies**

| **Study** | **Variable** | **Description** | **Secondary Structure** |
| --- | --- | --- | --- |
| ROS/MAP | Q12amme | Spell WORLD forwards |  |
| ROS/MAP | Q14mme | [SHOW WRIST WATCH] What is this called? |  |
| ROS/MAP | Q15mme | [SHOW PENCIL] What is this called? |  |
| ROS/MAP | Q16mme | Repeat a phrase |  |
| ROS/MAP | Q17mme | Read the words on this card, then do what it says |  |
| ROS/MAP | paper | Takes piece of paper |  |
| ROS/MAP | folds | Folds paper in half |  |
| ROS/MAP | places | Places paper in lap |  |
| ROS/MAP | Q19mme | Write any complete sentence |  |
| ROS/MAP | dnaming | What is the name of this object? |  |
| ROS/MAP | clothing | all of the things that belong in that category |  |
| ROS/MAP | animals | all of the things that belong in that category | F1 |
| ROS/MAP | fruits | all of the things that belong in that category | F1 |
| ROS/MAP | sink1 | Will a board sink in water? |  |
| ROS/MAP | sink2 | Will a stone sink in water? |  |
| ROS/MAP | hammer1 | Is a hammer good for cutting wood? |  |
| ROS/MAP | hammer2 | Can you use a hammer to pound nails? |  |
| ROS/MAP | flour1 | Do two pounds of flour weigh more than one? |  |
| ROS/MAP | flour2 | Is one pound of flour heavier than two? |  |
| ROS/MAP | boots1 | Will water go through a good pair of rubber boots? |  |
| ROS/MAP | boots2 | Will a good pair of rubber boots keep water out? |  |

### Supplemental Table 12. Co-calibration of language across ACT, ADNI, ROS/MAP

| **Study** | **Variable** | **Description** | **Secondary structure** |
| --- | --- | --- | --- |
| ACT, ADNI, ROS/MAP | read | Read the words on this card, then do it |  |
| ACT, ADNI, ROS/MAP | cmd | Paper, fold, place on floor combined |  |
| ACT, ADNI, ROS/MAP | catanim | Category Fluency (Animals)—Total Correct | F3 |
| ACT, ROS/MAP | bnt_name | Boston Naming: Name of this object? | F2 |
| ADNI, ROS/MAP | watch | [SHOW WRIST WATCH] What is this called? |  |
| ADNI, ROS/MAP | pencil | [SHOW PENCIL] What is this called? |  |
| ADNI, ROS/MAP | repeat | I would like you to repeat a phrase after me |  |
| ADNI, ROS/MAP | write | Write any complete sentence on this piece of |  |
| ROS/MAP | Q12amme | Spell WORLD forwards |  |
| ROS/MAP | clothing | all of the things that belong in that category |  |
| ROS/MAP | fruits | all of the things that belong in that category | F3 |
| ROS/MAP | sink1 | Will a board sink in water? |  |
| ROS/MAP | sink2 | Will a stone sink in water? |  |
| ROS/MAP | hammer1 | Is a hammer good for cutting wood? |  |
| ROS/MAP | hammer2 | Can you use a hammer to pound nails? |  |
| ROS/MAP | flour1 | Do two pounds of flour weigh more than one? |  |
| ROS/MAP | flour2 | Is one pound of flour heavier than two? |  |
| ROS/MAP | boots1 | Will water go through a good pair of rubber boots? |  |
| ROS/MAP | boots2 | Will a good pair of rubber boots keep water out? |  |
| ACT | bnt_adpr | Boston Naming Test | F2 |
| ACT-CASI | animal | animals with 4 legs | F3 |
| ACT-CASI | rpta | repeat something |  |
| ACT-CASI | rptb | repeat something |  |
| ACT-CASI | cas_writ | write something |  |
| ACT-CASI | body | identify part of body |  |
| ACT-CASI | obja | identify object—1 | F1 |
| ACT-CASI | objb | identify object—2 | F1 |
| ADNI | catvegesc | Category Fluency (VegSupplemental Tables) —Total Correct | F3 |
| ADNI | bnttotal | Total Number Correct (1+3) | F3 |
| ADNI | q2score | ADAS Commands |  |
| ADNI | q5score | ADAS Naming | F3 |
| ADNI | q6score | Ideational Praxis—score |  |
| ADNI | camel | Camel |  |
| ADNI | lion | Lion |  |
| ADNI | rhino | Rhinoceros |  |
| ADNI | repeat1 | Repeat Sentence. |  |
| ADNI | repeat2 | Repeat Sentence. |  |
| ADNI | ffluency | Letter Fluency—F: Total # of correct words |  |

## Visuospatial FUNCTIONING

### ACT: Final model was a data driven bifactor model with CFI = 0.993, TLI = 0.987, and RMSEA = 0.031. The following items were included in the CFA analysis (Supplemental Table 13):

**Supplemental Table 13. Items and secondary structure for visuospatial functioning for the ACT study**

| **Study** | **Variable** | **Description** | **Secondary Structure** |
| --- | --- | --- | --- |
| ACT | mat_cons | Mattis Dementia Rating Scale—constructional praxis score |  |
| ACT | cp_in_ci | Constructional Praxis—circle | F1 |
| ACT | cp_in_di | Constructional Praxis—diamond | F1 |
| ACT | cp_in_re | Constructional Praxis—rectangles |  |
| ACT | cp_in_cu | Constructional Praxis—cube |  |
| ACT-CASI | draw | Copy interlocking pentagons |  |

### ADNI: Final model was a single factor model with CFI = 0.958, TLI = 0.937, and RMSEA = 0.060. The following items were included in the CFA analysis (Supplemental Table 14):

**Supplemental Table 14. Items and secondary structure for visuospatial functioning for the ADNI study**

| **Study** | **Variable** | **Description** | **Secondary Structure** |
| --- | --- | --- | --- |
| ADNI | copycirc | Clock copy: Approximately circular face |  |
| ADNI | copysym | Symmetry of number placement |  |
| ADNI | copynum | Correctness of numbers |  |
| ADNI | copyhand | Presence of the two hands |  |
| ADNI | copytime | Presence of the two hands, set to ten after eleven |  |
| ADNI | q3score | Constructional Praxis—score |  |
| ADNI | mmdraw | Present the participant with the Cnstrn Stimulus page. Say: Copy this design |  |

### ROS/MAP: Final model was a single factor model with CFI = 0.948, TLI = 0.940, and RMSEA = 0.040. The following items were included in the CFA analysis (Supplemental Table 15):

**Supplemental Table 15. Items and secondary structure for visuospatial functioning for the ROS and MAP studies**

| **Study** | **Variable** | **Description** | **Secondary Structure** |
| --- | --- | --- | --- |
| ROS/MAP | Q20mme | Please copy the drawing on this piece of paper |  |
| ROS/MAP | Line1…15 | Which two lines point in the same direction…? |  |

### Co-calibration of visuospatial ability across ACT, ADNI, ROS/MAP

Single factor models were selected for ADNI and ROS/MAP, while a bifactor model with a single residual correlation was chosen for the ACT study. The pair of items with a residual correlation from ACT was unique to ACT and not present in either of the other studies. Our final model was thus a bifactor model that included only a single residual correlation for the pair of items from the ACT study; all other items, including all of the items administered in ROS/MAP and ADNI, only had loadings on the general factor.

**Supplemental Table 16: Co-calibration of visuospatial ability across ACT, ADNI, and ROS/MAP.**

| **Study** | **Variable** | **Description** | **Secondary Structure** |
| --- | --- | --- | --- |
| ACT, ADNI, ROS/MAP | Q20mme | Please copy the drawing on this piece of paper |  |
| ROS/MAP | Line1–15 | Which two lines point in the same direction… |  |
| ACT | mat_cons | Mattis Dementia Rating Scale |  |
| ACT | cp_in_ci | Constructional Praxis—circle | F1 |
| ACT | cp_in_di | Constructional Praxis—diamond | F1 |
| ACT | cp_in_re | Constructional Praxis—rectangles |  |
| ACT | cp_in_cu | Constructional Praxis—cube |  |
| ADNI | copycirc | Clock copy: Approx circular face |  |
| ADNI | copysym | Symmetry of number placement |  |
| ADNI | copynum | Correctness of numbers |  |
| ADNI | copyhand | Presence of the two hands |  |
| ADNI | copytime | Presence of the two hands, set to ten after eleven |  |
| ADNI | q3score | Constructional Praxis—score |  |

# Supplemental Text 3: Choice of the threshold of 0.80 points

There is no readily agreed upon threshold for what would represent a substantial difference. We used data from ACT, since it was the largest community-based cohort study for which we had data. We considered a range of thresholds from 0.40 to 1.25 SD. At each threshold, we noted in how many and in which domains each individual had relative impairments. We categorized people as having no domain with a substantial relative impairment vs. having each single domain with a substantial relative impairment vs. having more than one domain with a substantial relative impairment.

Based on results from those analyses we selected a threshold of 0.80 to consider further. We selected that threshold based on the inflection in the curve describing the proportion of people with more than one domain with a substantial relative impairment. We reasoned that at insufficiently strenuous thresholds, multiple domains could be impaired just by chance alone, such that there would be a mixture of people with substantial relative deficits in multiple domains together with people with low scores just by chance. Below the 0.80 inflection point, the proportion of people identified with substantial relative impairments in multiple domains was much less influenced by the threshold than it was above the 0.80 inflection point.

The proportion of ACT participants with incident Alzheimer’s dementia who met criteria for each cognitively defined subtype at each threshold level is shown in Supplemental Figure 1.

| **Supplemental Figure 1.** Cognitively-defined subgroup membership at each threshold level* |
| --- |
| **** |
| * Data represent the proportion of people with incident Alzheimer’s dementia from ACT who met criteria for each subtype at thresholds that ranged from -0.40 to -1.25. At the -0.40 threshold, about 22% of the cohort had a memory score that was at least 0.40 points lower than their average score across all four domains, and had no other domain with a substantial relative impairment at that level (dark blue line). Also at the -0.40 threshold, about 16% of the cohort had a substantial relative impairment in visuospatial functioning and no other domain (purple line), about 13% had a substantial relative impairment in language (green line), and about 11% had a substantial relative impairment in executive functioning (red line); in all, 22% (memory) + 16% (visuospatial) + 13% (language) + 11% (executive functioning) = 62% of the cohort had a substantial relative impairment in a single domain at the minus 0.40 threshold. The light blue line shows that at that same threshold, about 23% of the cohort had two or more domains with a substantial relative impairment, meaning that in all 85% of the cohort had 1 or more domains with a substantial relative impairment at that threshold and the remaining 15% had no domains with a substantial relative impairment. Moving to the right, the proportion of individuals identified with domains with a substantial relative impairment decreases. There appears to be an inflection point in the light blue curve that represents the proportion of the cohort with substantial relative impairments in more than one domain around a threshold of minus 0.80; we selected that threshold for further analyses. |

**Supplemental Text 4. Sensitivity of *APOE* findings to choice of threshold**

We performed additional analyses to determine whether the *APOE* finding of significant differences across subsets was due to choice of the threshold of 0.80. We used combined data from all the studies. We categorized people at each threshold between -0.40 to -1.25. We performed two analyses. First, we considered the proportion in each subgroup with a χ^2^ test with 5 degrees of freedom. We plot the –log_10_ of p values for each threshold in Supplemental Figure 2:

| **Supplemental Figure 2.** Sensitivity analyses of *APOE* ε4 proportions across subgroups at thresholds ranging from -0.40 to -1.25. |
| --- |
|  |

This figure shows p values ranging from 10^-17^ to 10^-32^. The value at 0.80 is similar to those across a wide range of thresholds.

**Supplemental Text 5. Addition of University of Pittsburgh study to the pipeline**

As detailed above, items where the PITT item was the same as an item with parameters from the ACT/ADNI/ROS-MAP analyses, we used those previously co-calibrated parameters. For items administered only to PITT participants, we freely estimated item parameters from the data set.

### Supplemental Table 17. Memory specification for the PITT dataset

| **Study** | **Variable** | **Description** | **Secondary Structure** |
| --- | --- | --- | --- |
| ROS/MAP, PITT | wrec | Word recognition trial 1 | F2 |
| ROS/MAP, PITT | wrec2 | Word recognition trial 2 | F2 |
| ROS/MAP, PITT | wrec3 | Word recognition trial 3 | F2 |
| ROS/MAP, PITT | wrecde | Word recognition—delayed |  |
| PITT | targets | Word recognition—target correct |  |
| PITT | foils | Word recognition—foils correct |  |
| PITT | reyim | Rey figure—immediate recall | F3 |
| PITT | reyde | Rey figure—delayed | F3 |
| ACT, ADNI, PITT | logimem | Logical Memory A1 | F1 |
| ACT, ADNI, PITT | memunits | Logical Memory A2 | F1 |
| ACT, PITT | mattism | Mattis DRS—memory |  |

### Supplemental Table 18. Executive function specification for the PITT dataset

| **Study** | **Variable** | **Description** | **Secondary Structure** |
| --- | --- | --- | --- |
| ROS/MAP, ADNI, PITT | spansb | Digit span—backwards | F1 |
| ACT, ADNI, PITT | trailas | Trail A—time | F2 |
| ACT, ADNI, PITT | trailbs | Trail B—time | F2 |
| PITT | mbar | Abstract reasoning |  |
| ACT, PITT | mattisip | Mattis DRS—initiation/perseveration |  |
| ACT, PITT | mconcep | Mattis DRS—conceptualization |  |
| ROS/MAP, ADNI, PITT | spansf | Digit span—forward | F1 |
| ACT, PITT | mattisa | Mattis DRS—attention |  |
| PITT | stpcw | Stroop—Color,Word |  |

### Supplemental Table 19. Language specification for the PITT dataset

| **Study** | **Variable** | **Description** | **Secondary Structure** |
| --- | --- | --- | --- |
| ACT, ADNI, ROS/MAP, PITT | fluen | Fluency test—animals | F1 |
| PITT | fluenb | Fluency test—birds | F1 |
| PITT | fluend | Fluency test—dogs | F1 |
| ADNI, PITT | fluenf | Fluency test—letter F | F3 |
| PITT | fluena | Fluency test—letter A | F3 |
| PITT | fluens | Fluency test—letter S | F3 |
| PITT | stpw | Stroop—Word | F2 |
| PITT | stpc | Stroop—Color | F2 |
| ADNI, PITT | veg | Category fluency—vegSupplemental Tables | F1 |
| ADNI, PITT | boston | Boston Naming Test total | F1 |

### Supplemental Table 20. Visuospatial functioning specification for the PITT dataset.

| **Study** | **Variable** | **description** | **Secondary Structure** |
| --- | --- | --- | --- |
| ACT, ADNI, ROS/MAP, PITT | pentagon | draw intersecting pentagons |  |
| PITT | reyco | Rey figure—copy |  |
| PITT | blkdsn | Block design |  |
| ACT, PITT | mconst | Mattis DRS—construction |  |

**Supplemental Text 6. Genetic analyses**

**Text 6A. Cohorts**

### The following description of the IGAP data sets has been duplicated from the supplemental materials of the IGAP gene-wide analysis paper^5^ referenced below.

**The ACT/eMERGE Studies (ACT)**The ACT cohort is an urban and suburban elderly population from a stable HMO that includes 2,581 cognitively intact subjects age ≥ 65 who were enrolled between 1994 and 1998^6, 7^. An additional 811 subjects were enrolled in 2000-2002 using the same methods except oversampling clinics with more minorities. More recently, a Continuous Enrollment strategy was initiated in which new subjects are contacted, screened and enrolled to keep 2000 active at-risk person-years accruing in each calendar year. This resulted in an enrollment of 4,146 participants as of May 2009. All clinical data are reviewed at a consensus conference. Dementia onset is assigned half way between the prior biennial and the exam that diagnosed dementia. Enrollment for the eMERGE Study began in 2007. A waiver of consent was obtained from the IRB to enroll deceased ACT participants. For this study we analyzed genome-wide genetic data from 1,407 cognitively normal elderly controls and 457 people with late-onset Alzheimer’s disease.

**The ADNI Study (ADNI 1/GO/2)**ADNI is a longitudinal, multi-site observational study including people with Alzheimer’s disease, people with mild cognitive impairment (MCI), and elderly individuals with normal cognition assessing clinical and cognitive measures, MRI and PET scans (FDG and 11C PIB) and blood and CNS biomarkers. For this study, ADNI contributed data on 607 Alzheimer’s disease cases and 325 healthy controls with Alzheimer’s disease -free status confirmed as of most recent follow-up. Alzheimer’s disease subjects were between the ages of 65–90, had an MMSE score of 20–26 inclusive, met NINCDS/ADRDA criteria for probable Alzheimer’s disease ^8^, and had an MRI consistent with the diagnosis of Alzheimer’s disease. Control subjects had MMSE scores between 28 and 30 and a Clinical Dementia Rating of 0 without symptoms of depression, MCI or other dementia and no current use of psychoactive medications. According to the ADNI protocol, subjects were ascertained at regular intervals over 3 years, but for the purpose of our analysis we only used the final ascertainment status to classify case-control status. Additional details of the study design are available elsewhere^9-11^.

Data used in the preparation of this article were obtained from ADNI database (<http://adni.loni.ucla.edu>). The ADNI was launched in 2003 by the National Institute on Aging (NIA), the National Institute of Biomedical Imaging and Bioengineering (NIBIB), the Food and Drug Administration (FDA), private pharmaceutical companies and non-profit organizations, as a $60 million, 5-year public-private partnership. The primary goal of ADNI has been to test whether serial magnetic resonance imaging (MRI), positron emission tomography (PET), other biological markers, and clinical and neuropsychological assessment can be combined to measure the progression of mild cognitive impairment (MCI) and early Alzheimer’s disease Alzheimer’s disease. Determination of sensitive and specific markers of very early Alzheimer’s disease progression is intended to aid researchers and clinicians to develop new treatments and monitor their effectiveness, as well as lessen the time and cost of clinical trials. The Principal Investigator of this initiative is Michael W. Weiner, MD, VA Medical Center and University of California—San Francisco. ADNI is the result of efforts of many co-investigators from a broad range of academic institutions and private corporations, and subjects have been recruited from over 50 sites across the U.S. and Canada. The initial goal of ADNI was to recruit 800 subjects but ADNI has been followed by ADNI-GO and ADNI-2. To date these three protocols have recruited over 1500 adults, ages 55 to 90, to participate in the research, consisting of cognitively normal older individuals, people with early or late MCI, and people with early Alzheimer’s disease. The follow up duration of each group is specified in the protocols for ADNI-1, ADNI-2 and ADNI-GO. Subjects originally recruited for ADNI-1 and ADNI-GO had the option to be followed in ADNI-2. For up-to-date information, see [www.adni-info.org](http://www.adni-info.org).

For this study we analyzed genome-wide genetic data from 328 cognitively normal elderly controls and 589 people with late-onset Alzheimer’s disease.

**The ROS/MAP Studies**ROS/MAP are two community-based cohort studies. The ROS has been on-going since 1993, with a rolling admission. Through July of 2010, 1,139 older nuns, priests, and brothers from across the United States initially free of dementia who agreed to annual clinical evaluation and brain donation at the time of death completed their baseline evaluation. The MAP has been on-going since 1997, also with a rolling admission. Through July of 2010, 1,356 older persons from across northeastern Illinois initially free of dementia who agreed to annual clinical evaluation and organ donation at the time of death completed their baseline evaluation. Details of the clinical and neuropathologic evaluations have been previously reported^12-15^.

For this study we analyzed genome-wide genetic data from 825 cognitively normal elderly controls and 673 people with late-onset Alzheimer’s disease.

**University of Pittsburgh (PITT)**The University of Pittsburgh data set contains 1,271 Caucasian Alzheimer’s disease cases (of which 277 were autopsy-confirmed) recruited by the University of Pittsburgh Alzheimer’s Disease Research Center, and 841 Caucasian, cognitively normal elderly controls ages 60 and older (2 were autopsy-confirmed). All Alzheimer’s disease cases met NINCDS/ADRDA criteria for probable or definite Alzheimer’s disease. Additional details of the cohort used for GWAS have been previously published^16^. We limited analyses from the Pittsburgh site to those with a Clinical Dementia Rating (CDR) of 0.5 or 1.0, since stability of cognitively-defined Alzheimer’s disease subgroups in more advanced degrees of severity has not been established.

For this study we analyzed genome-wide genetic data from 825 cognitively normal elderly controls and 712 people with late-onset Alzheimer’s disease.

**Text 6B. Imputation and SNP selection for GWAS analyses.**

Each of the raw (observed SNPs) genetic data sets from ACT/ADNI/ROS-MAP/UPITT were quality controlled and imputed using IMPUTE2 with haplotypes derived from samples of European ancestry in the 1000 Genome Project (2012 build) by the Alzheimer’s Disease Genetics Consortium. Detailed quality control procedures can be obtained from Lambert *et al^17^*. In each imputed data set, SNPs with *R*^2^ or info score quality estimates of less than 0.5 as indicated by IMPUTE2 were excluded from analyses. Similarly, SNPs with a MAF of <3% were also excluded. After these procedures, a maximum of 6,423,139 SNPs were retained in at least one data set across the different Alzheimer’s disease subtype analyses.

Alzheimer’s disease cases with Clinical Dementia Rating (CDR) of 0.5 or 1 were selected for each analyses. In each case-control data set for each subtype, the association of Alzheimer’s disease subtype with SNPs was analyzed by a logistic regression model including covariates for age, sex and principal components to account for possible population stratification. Relatedness analyses and principal components were performed using observed genotype data KING-Robust^18^ from the four studies. We used PLINK v1.9^19^ for GWAS analyses.

After the exclusion of SNPs showing logistic regression coefficient |*β*| > 5 or *p*-value equal to 0 or 1, the maximum number of SNPs in any data set for any of the subtype analyses was 6,398,204.

These SNPs were included in the meta-analysis.

**Text 6C. Meta-analysis.**

We conducted a meta-analysis of genome-wide association studies (GWAS) in individuals of European ancestry for each Alzheimer’s disease subtype except for the group with isolated substantial relative executive functioning impairment. That group was the smallest with cases ranging from 3 to 30 in each of the data sets. We used genotyped and imputed data (~6.4 million SNPs) to perform meta-analysis on four GWAS data sets (ACT/ADNI/ROS-MAP/PITT) with the exception of the group with multiple domains with substantial relative impairments. That group had only 12 individuals with genetic data from the ACT study so we excluded ACT from meta-analyses for that group. We undertook fixed-effects inverse variance-weighted meta-analysis using METAL^20^. SNPs that failed heterogeneity test (*p*-value ≤ 0.05) were excluded from the results.

The genomic control inflation factors (**λ**) for the meta-analysis of each Alzheimer’s disease subtype were 1.01 for the group with no domain with a substantial relative impairment, 1.01 for those with isolated substantial relative memory impairment, 1.0 for those with isolated substantial language impairment, 0.97 for those with isolated substantial visuospatial impairment, and 0.99 for those with multiple domains with substantial relative impairments. **λ** for All Alzheimer’s disease vs. controls analysis across the four studies was 1.01. Quantile-quantile plots for each analysis are shown in Supplemental Figure 3 (section 6D).

Manhattan plots for each meta-analysis of Alzheimer’s disease subtypes are shown in Supplemental Figure 4 (section 6E). Plots for each Alzheimer’s disease subtype are broken down into two plots; a) a full Manhattan plot and b) a truncated Manhattan plot with SNPs with *p*-value > 1×10^-10^. GWAS summary statistics of top hits for each Alzheimer’s disease subtype by study and overall meta-analysis are shown in Supplemental Text 7-11. Top hits for each Alzheimer’s disease subtypes with corresponding results for those in other Alzheimer’s disease subtypes are listed in tables in Supplemental Text 7-11.

Regional association plots^21^ for top SNP hits for each Alzheimer’s disease subtype are shown in Supplemental Figure 5 (Section 6F).

**6D. Q-Q plots from meta-analysis of GWAS results.**

| **Supplemental Figure 3. Quantile-Quantile plots** | |
| --- | --- |
| a) Controls vs. no substantial relative impairment | b) Controls vs. substantial relative memory impairment |
| 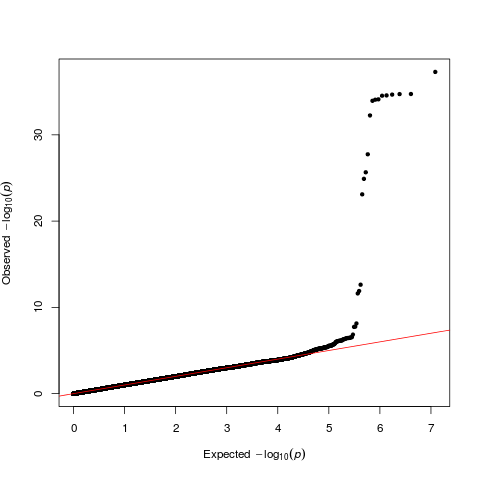 | 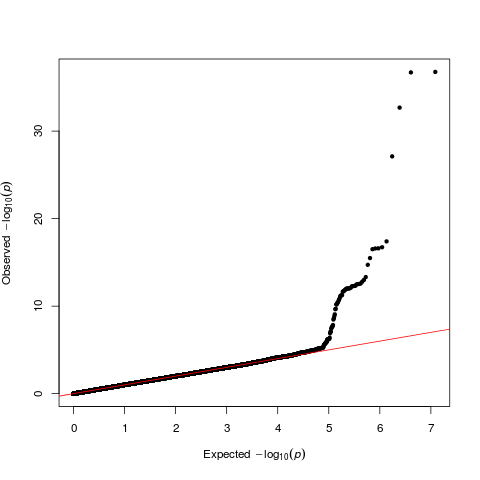 |
| c) Controls vs. substantial relative language impairment | d) Controls vs. substantial relative visuospatial impairment |
| 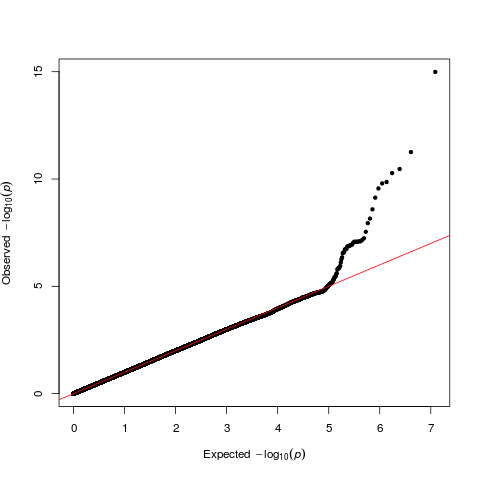 | 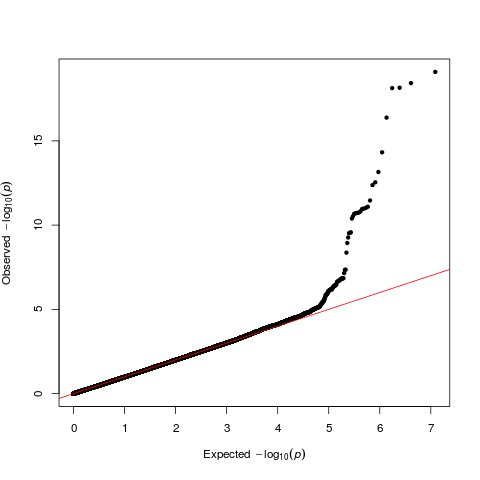 |
| e) Controls vs. multiple domains with substantial relative impairment | f) Controls vs. All Alzheimer’s disease across four studies |
| **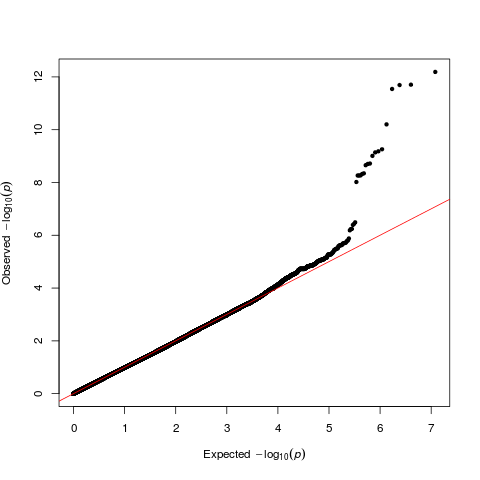** | **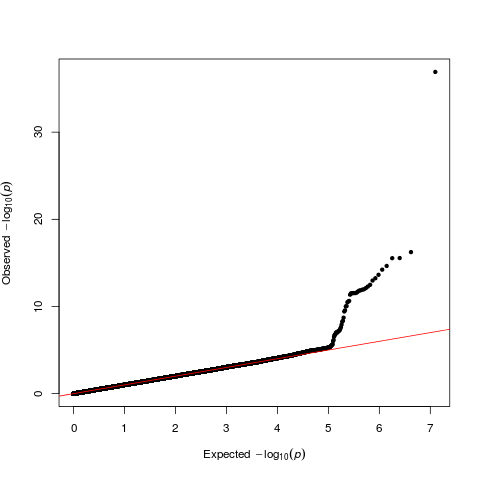** |

**6E. Manhattan plots from meta-analyses of GWAS results**

For each analysis, there are two plots: i) Full Manhattan plot; ii) Manhattan plot with *p*-values truncated to > 1×10^-10^.

| **Supplemental Figure 4. Manhattan plots from meta-analysis of GWAS results** |
| --- |
| Supplemental Figure 4a. Controls vs. no substantial relative impairment |
| **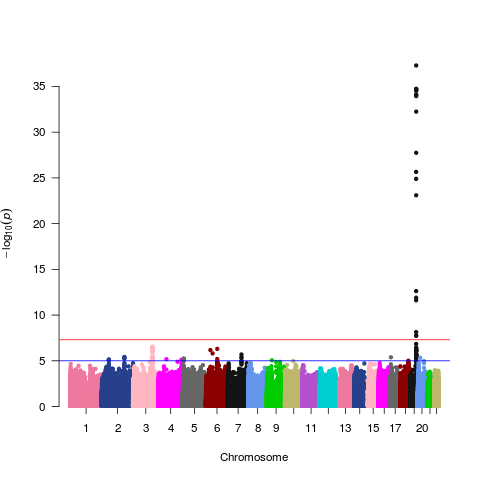** |
| **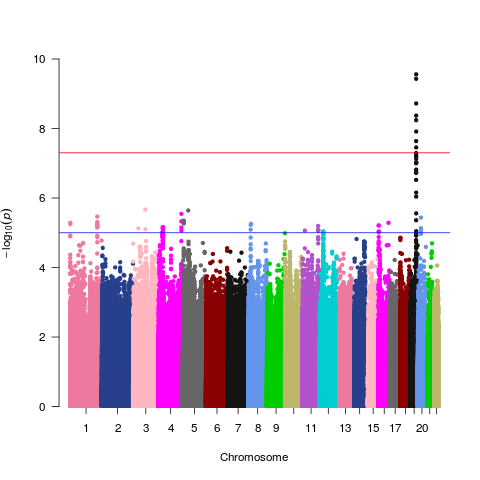** |

| Supplemental Figure 4b. Controls vs. substantial relative memory impairment |
| --- |
| **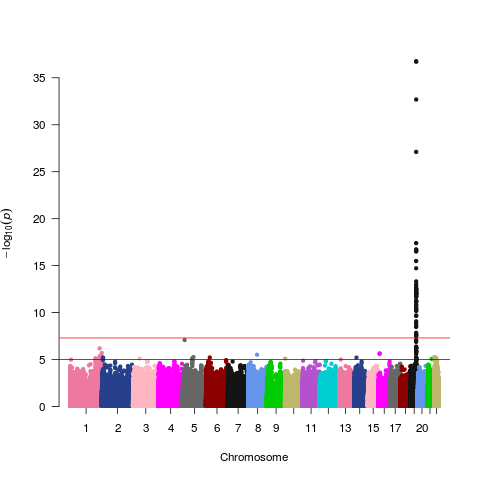** |
| **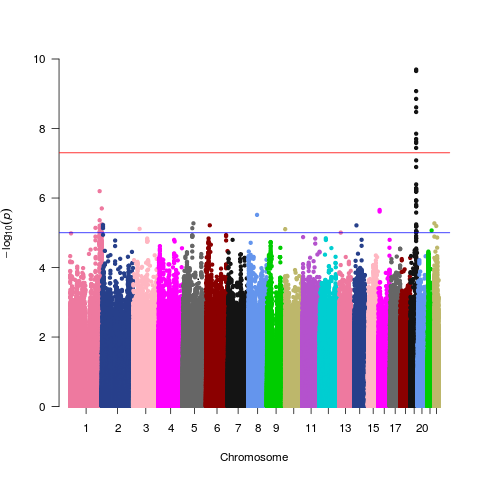** |

| Supplemental Figure 4c. Controls vs. substantial relative language impairment |
| --- |
| **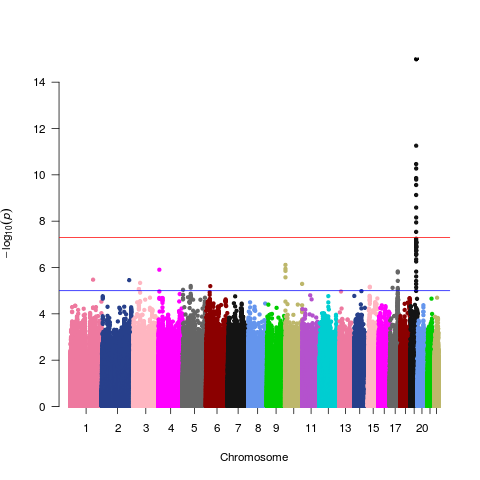** |
| **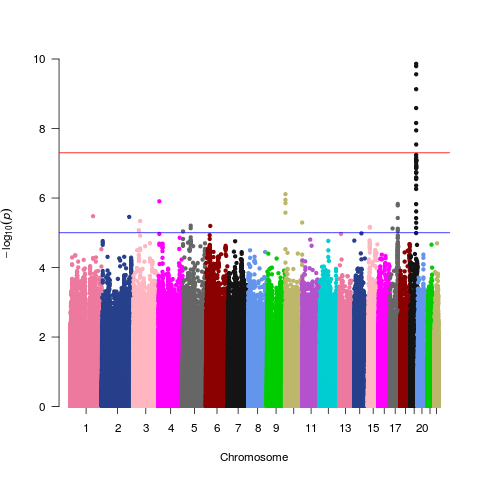** |

| Supplemental Figure 4d. Controls vs. substantial relative visuospatial impairment |
| --- |
| **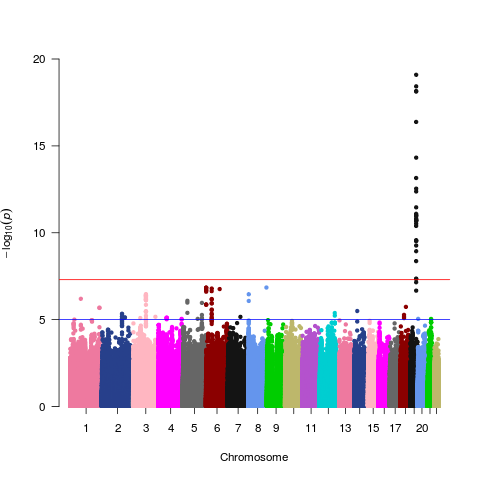** |
| **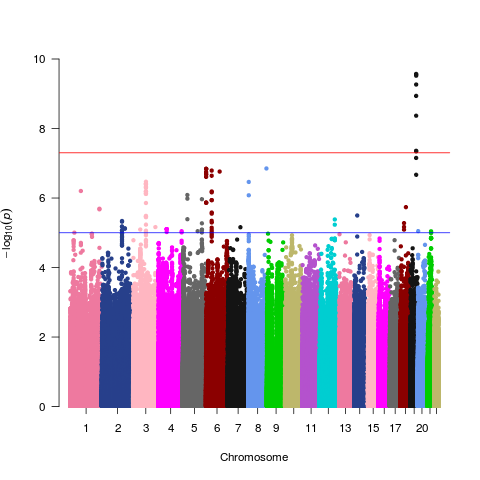** |

| eFogure 4e. Controls vs. multiple domains with substantial relative impairment |
| --- |
| **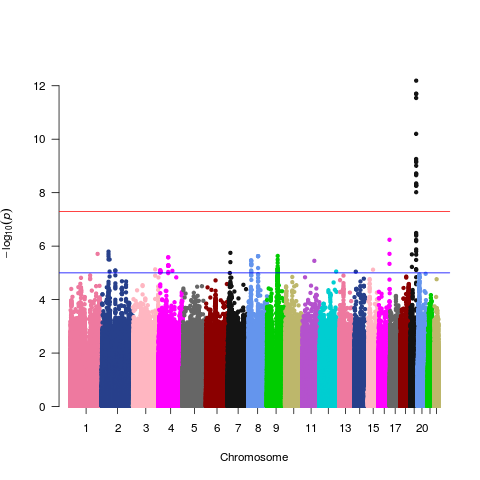** |
| **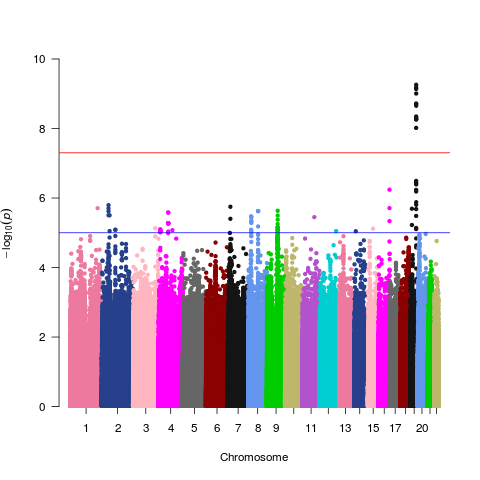** |

| Supplemental Figure 4f. Controls vs. All Alzheimer’s disease across four studies |
| --- |
| **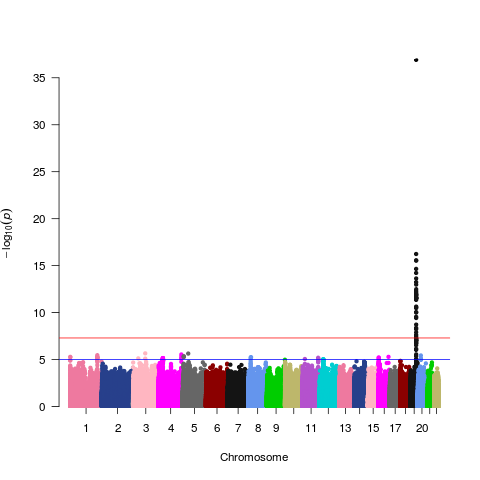** |
| **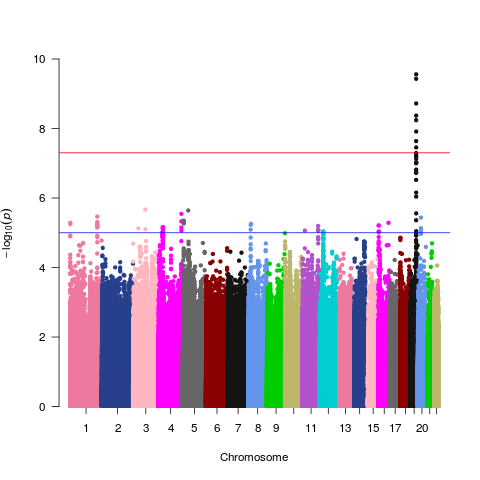** |

**6F. Regional association plots of top hits from meta-analyses for each Alzheimer’s disease subtype**

**Supplemental Figure 5. Regional association plots from meta-analysis for each Alzheimer’s disease subtype**

| **Supplemental Figure 5a. Controls vs. no substantial relative impairment** |
| --- |
| **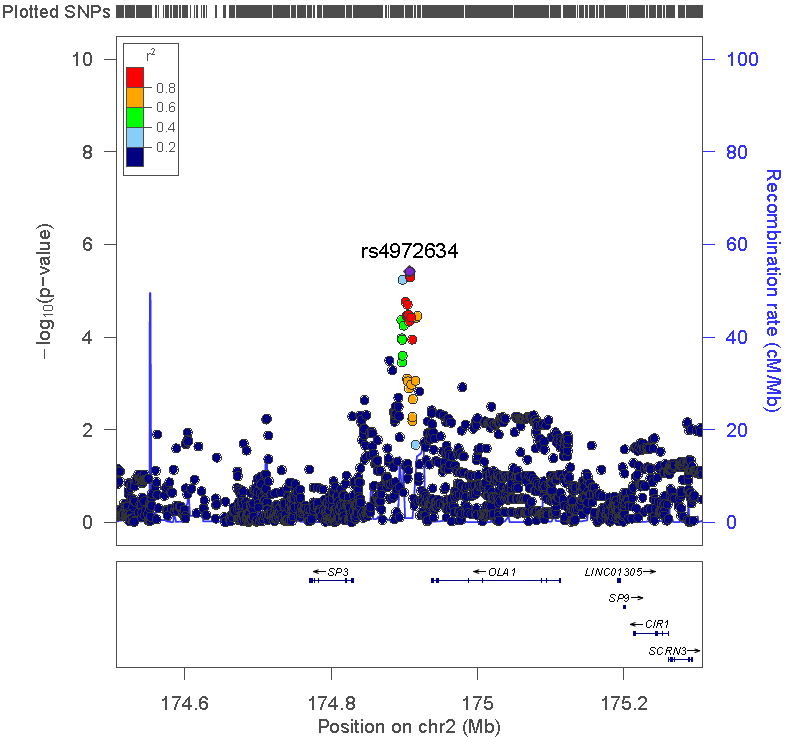** |
| **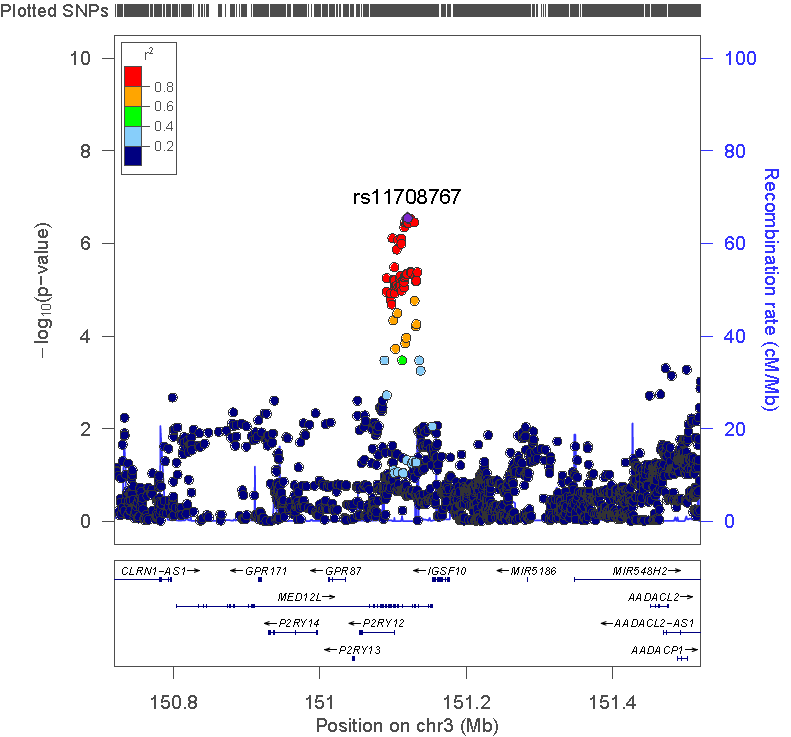** |
| **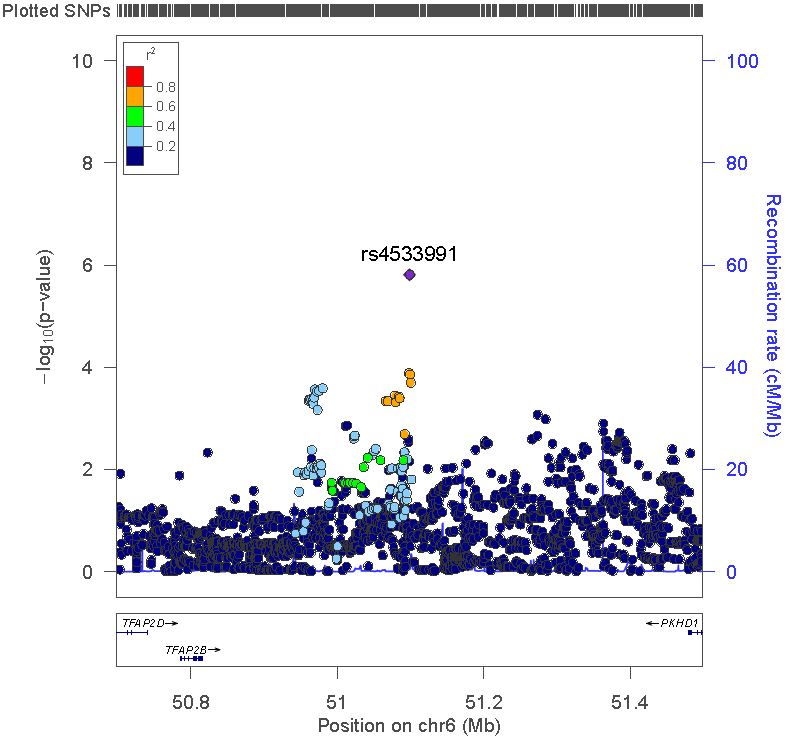** |

| **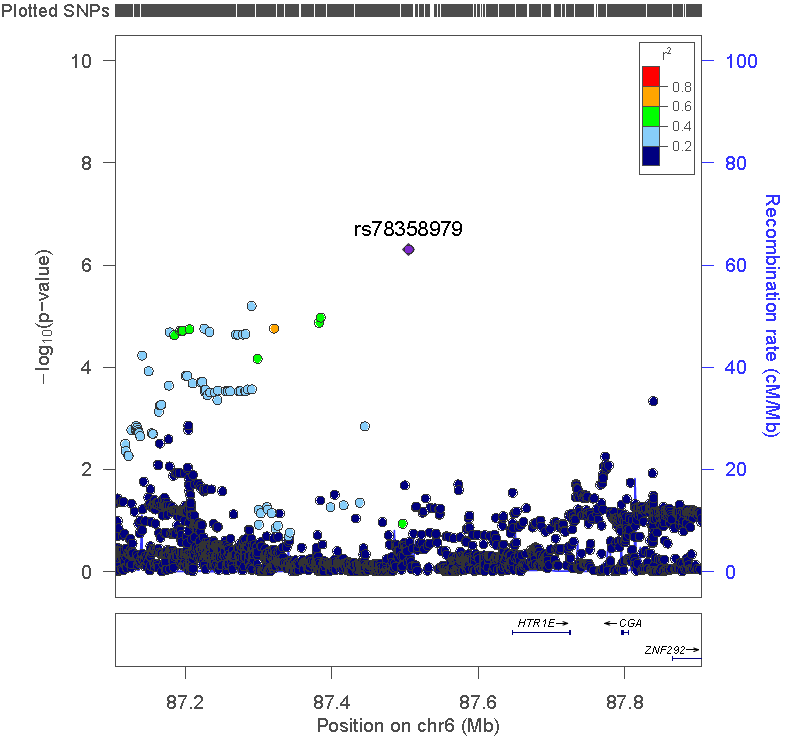** |
| --- |
| **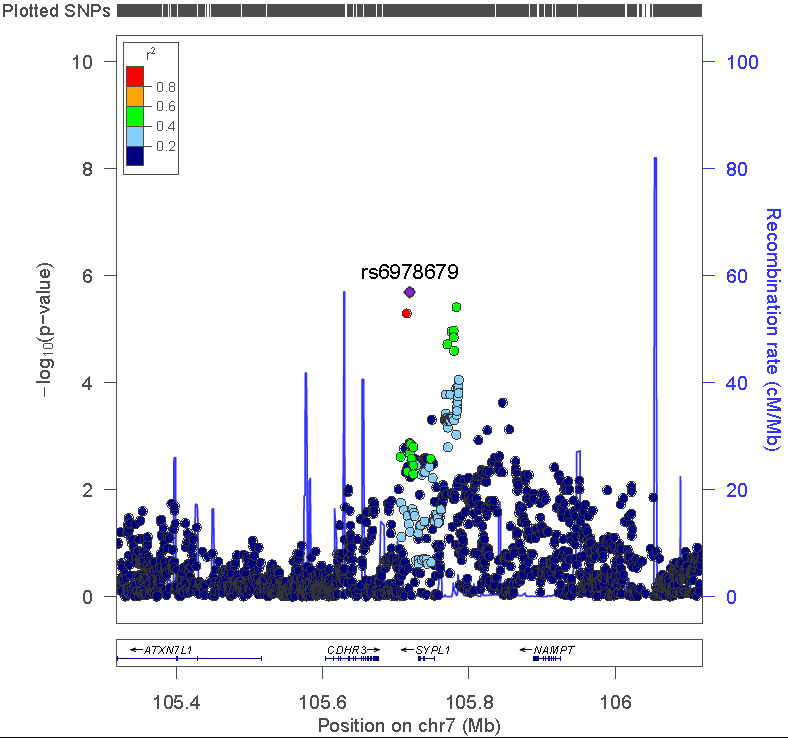** |

| **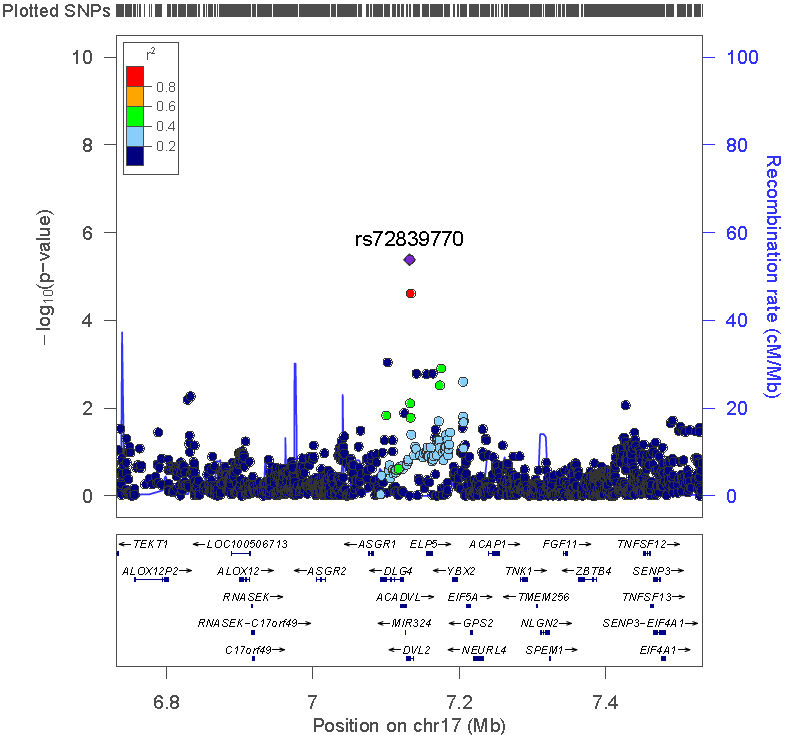** |
| --- |
| **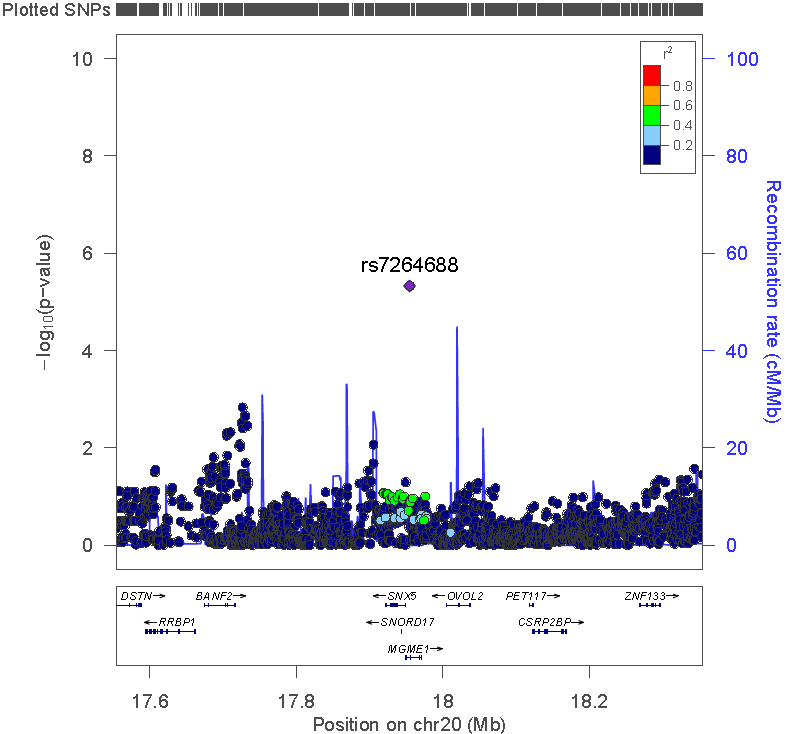** |

| Supplemental Figure 5b. Controls vs. substantial relative memory impairment |
| --- |
| **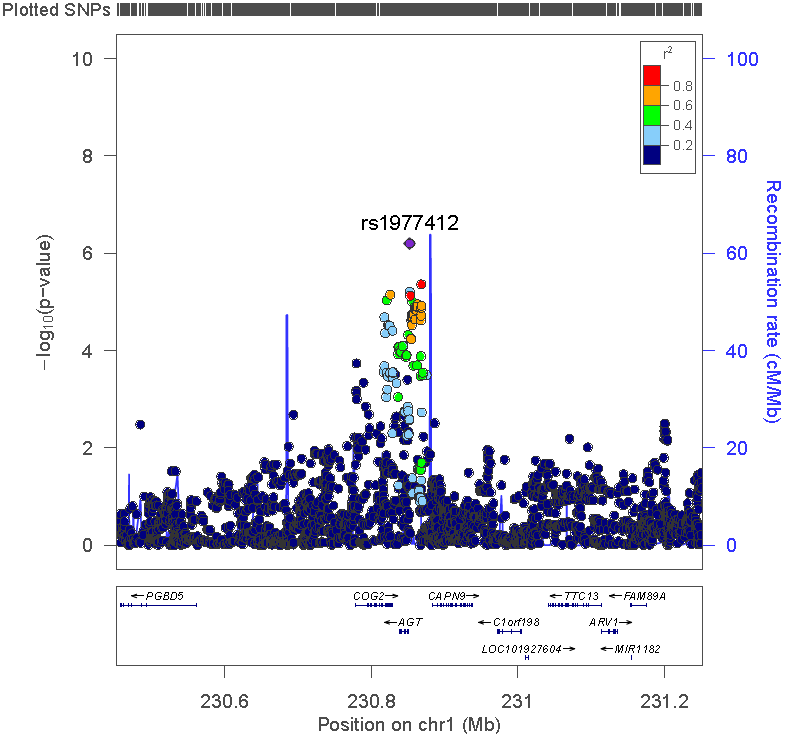** |
| **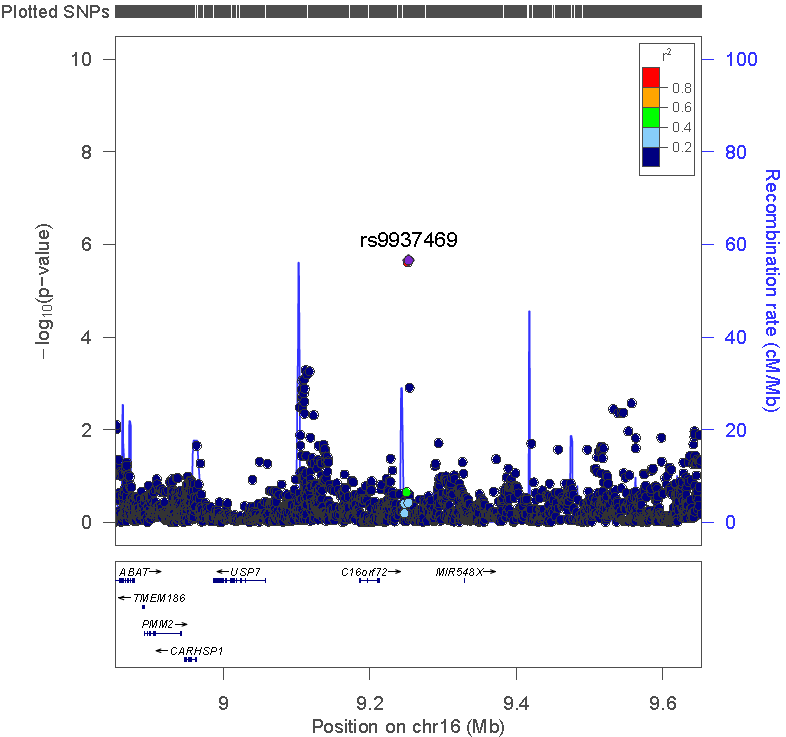** |

| Supplemental Figure 5c. Controls vs. substantial relative language impairment |
| --- |
| **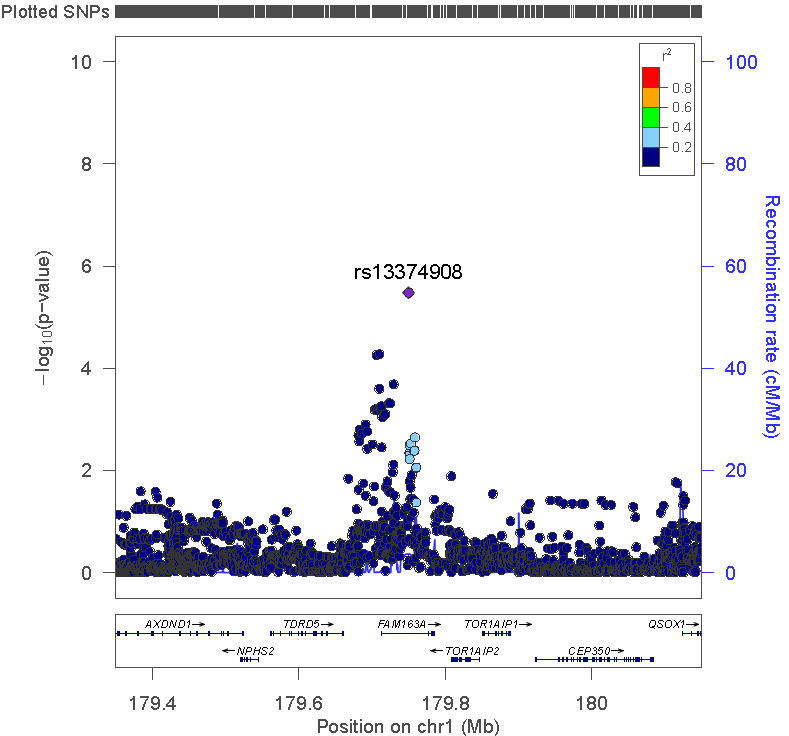** |
| **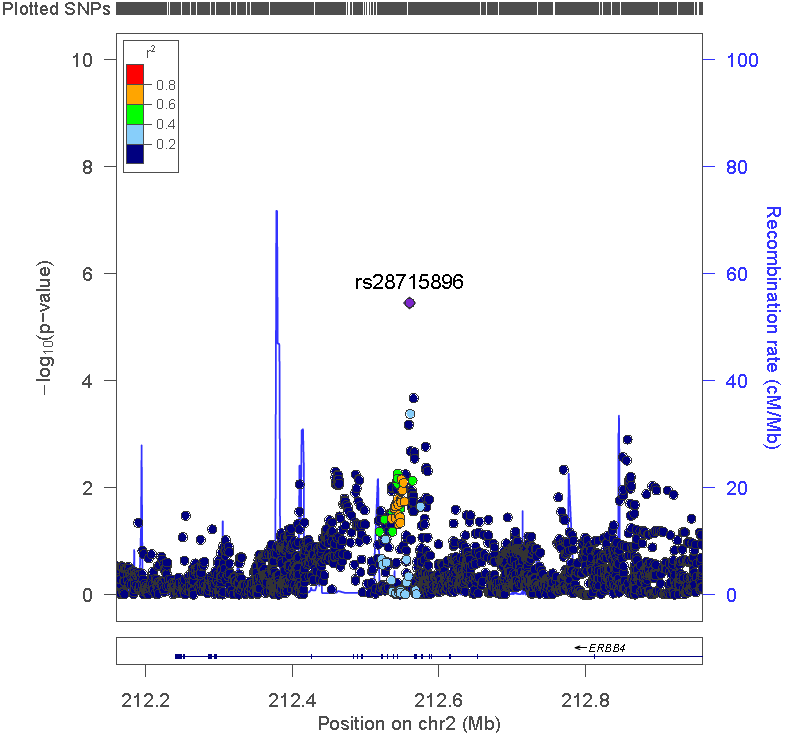** |
| **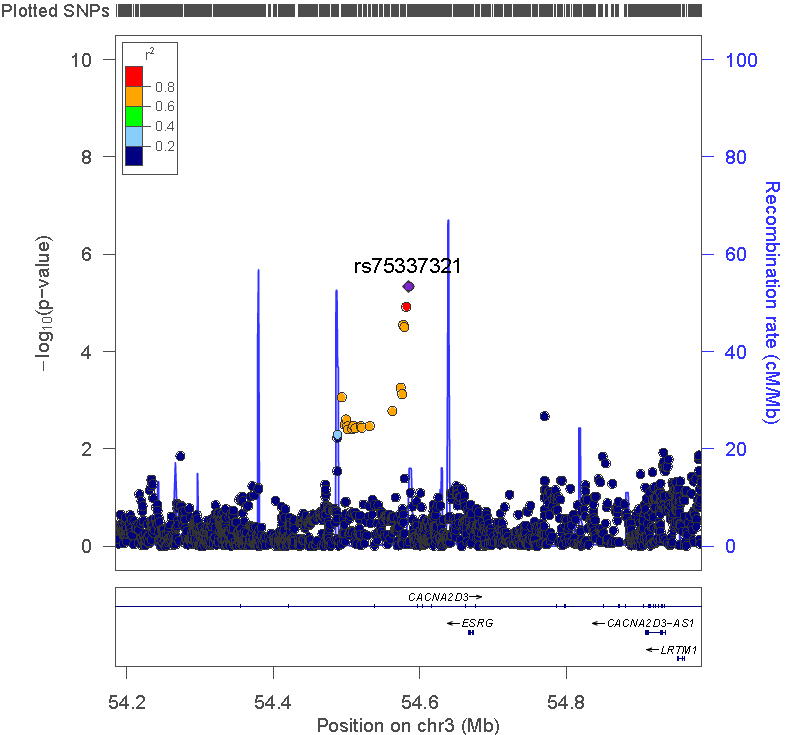** |
| **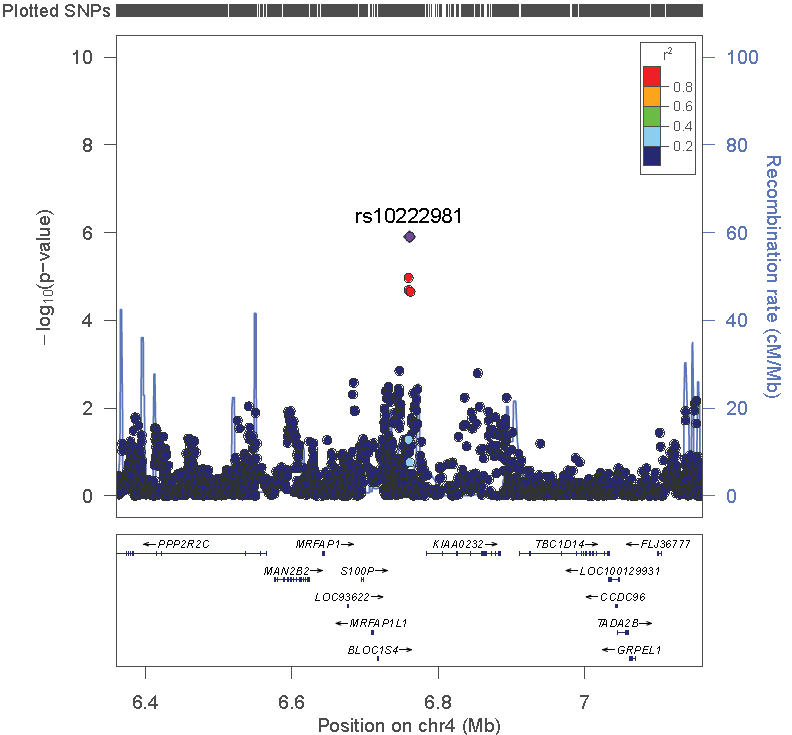** |
| **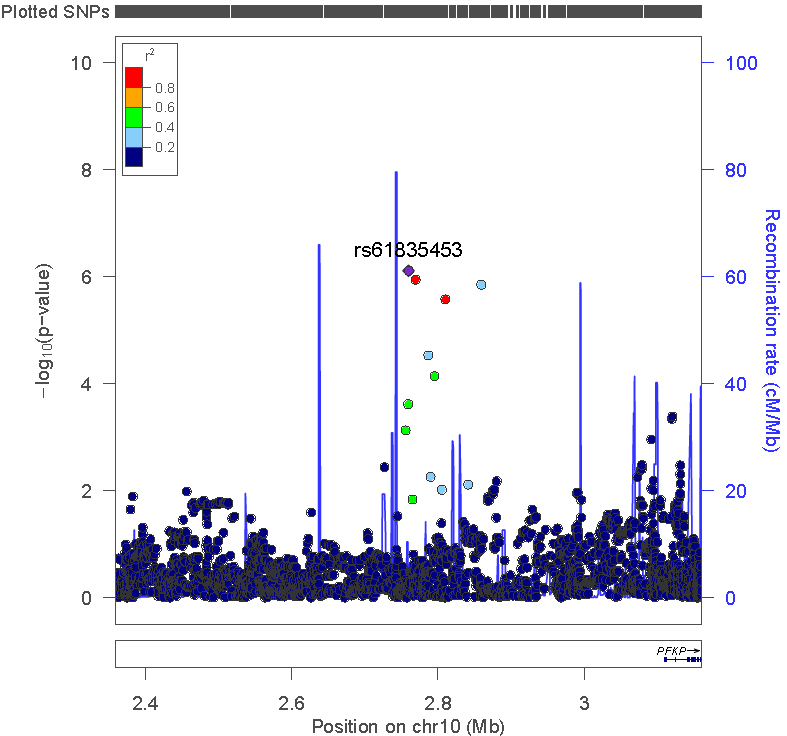** |
| **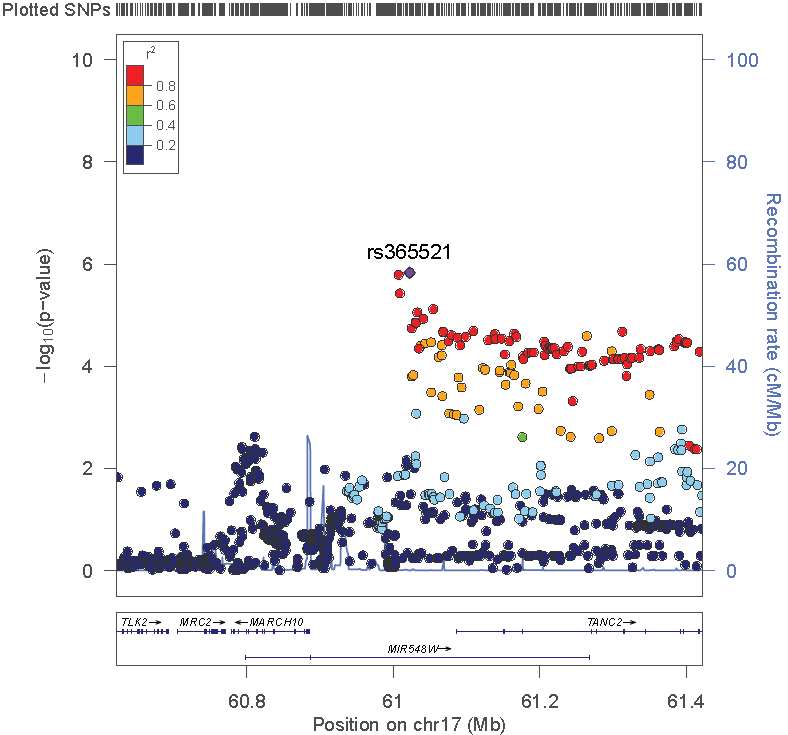** |

| Supplemental Figure 5d. Controls vs. substantial relative visuospatial impairment |
| --- |
| **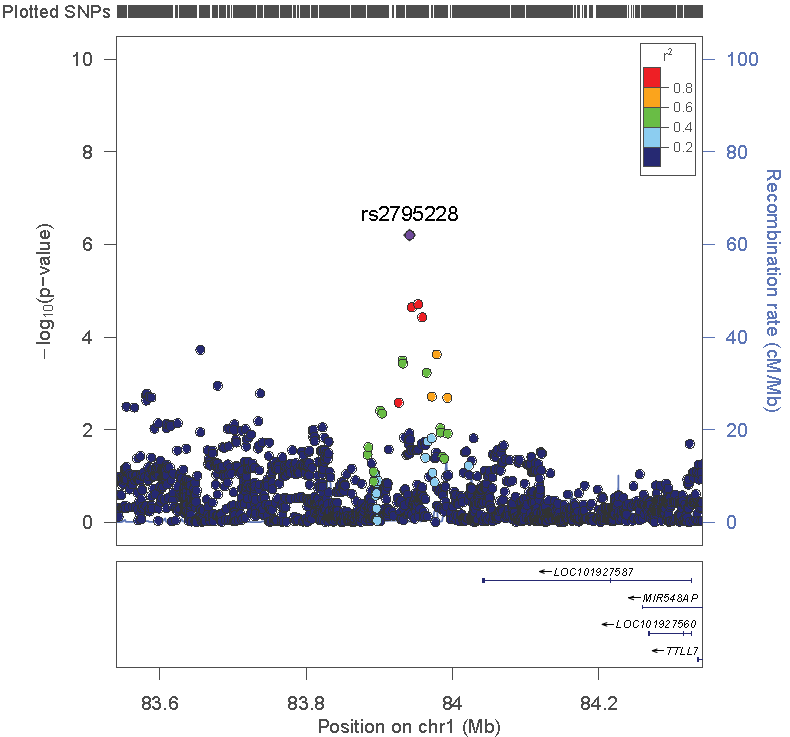** |
| **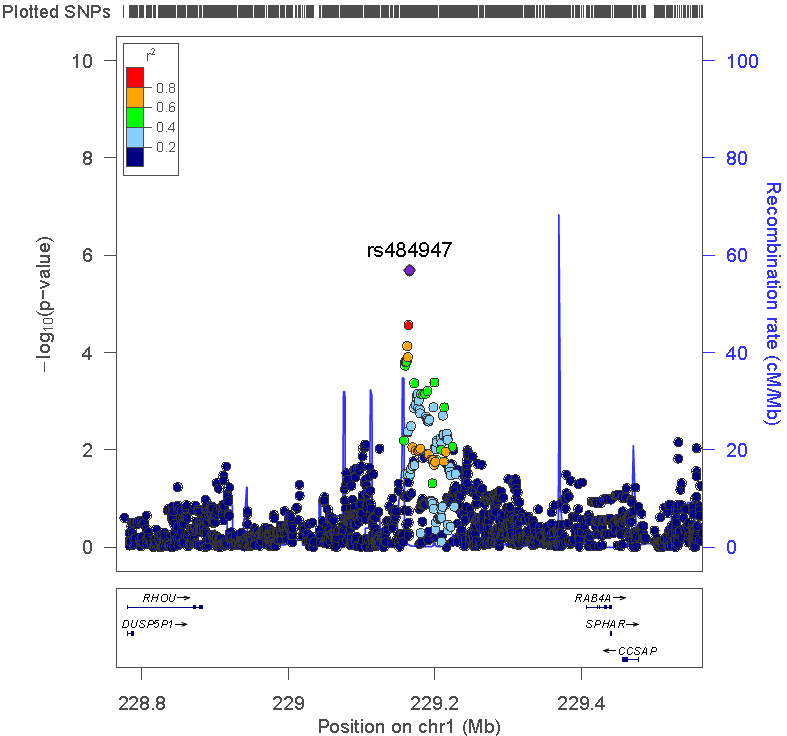** |
| **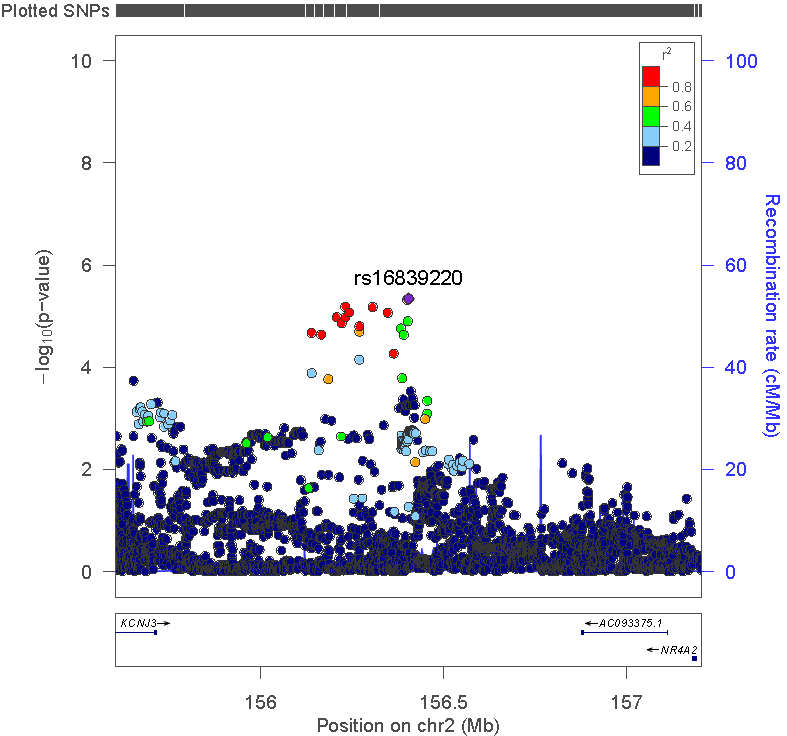** |
| **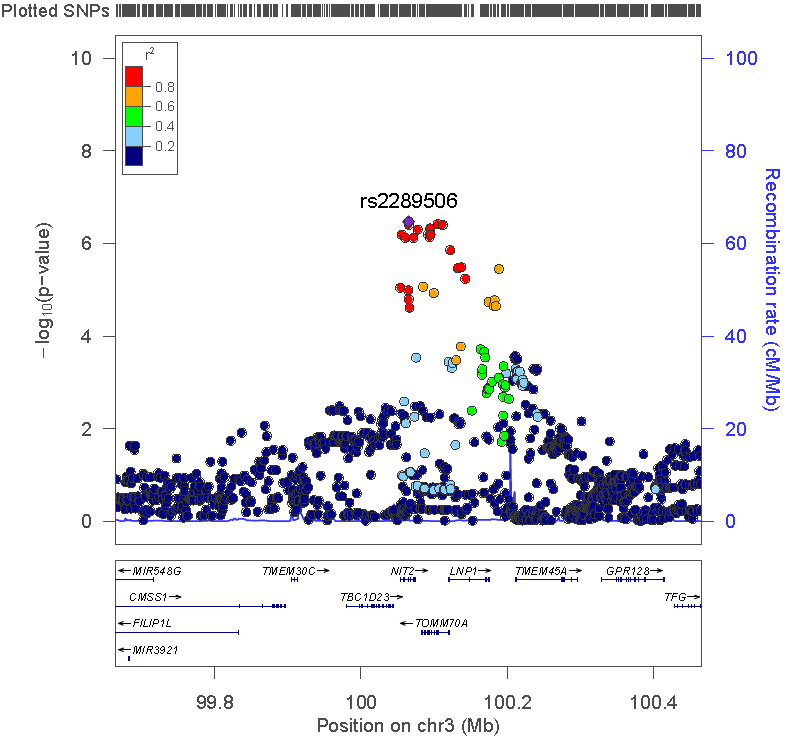** |
| **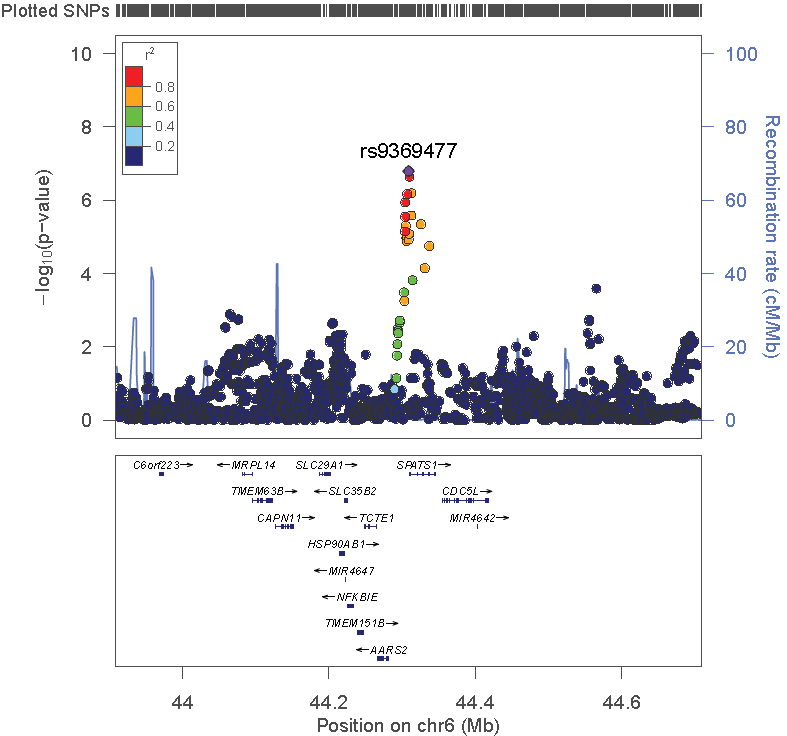** |
| **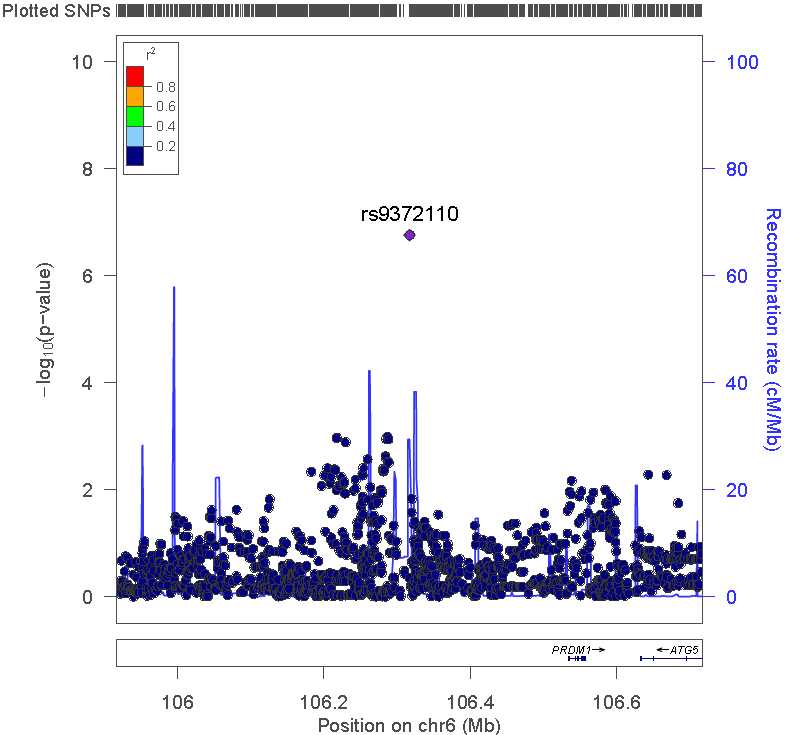** |
| **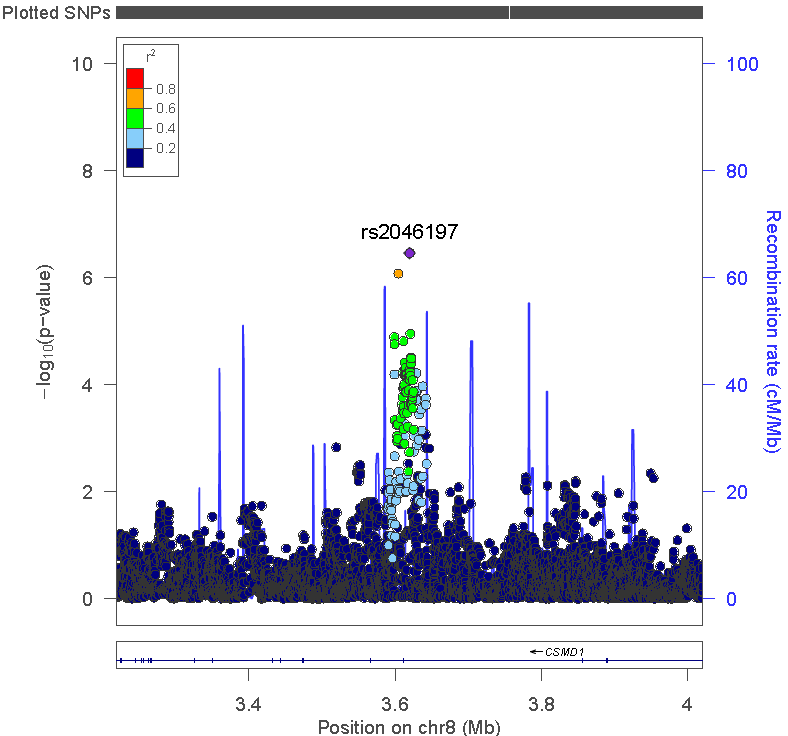** |
| **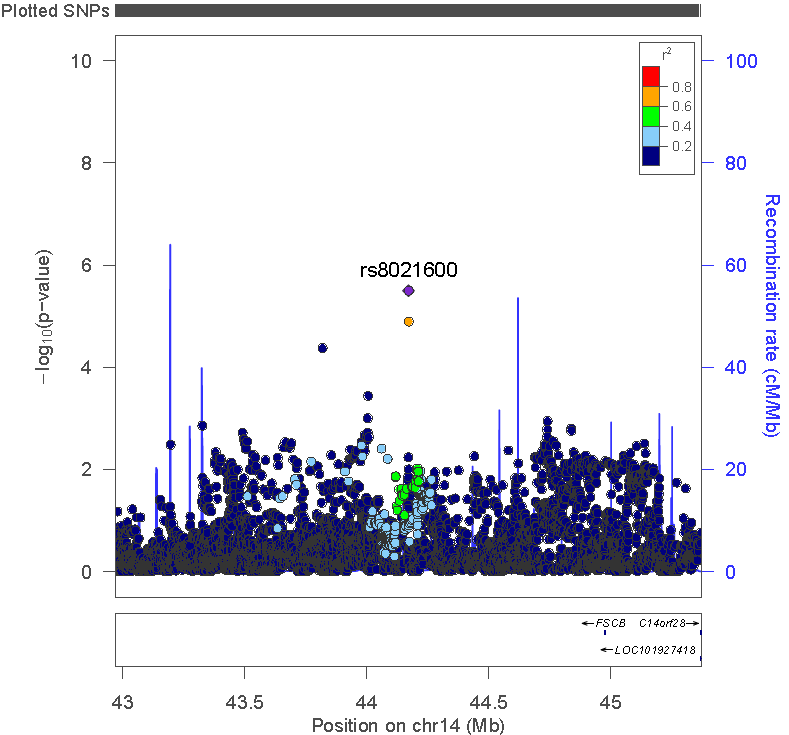** |
| **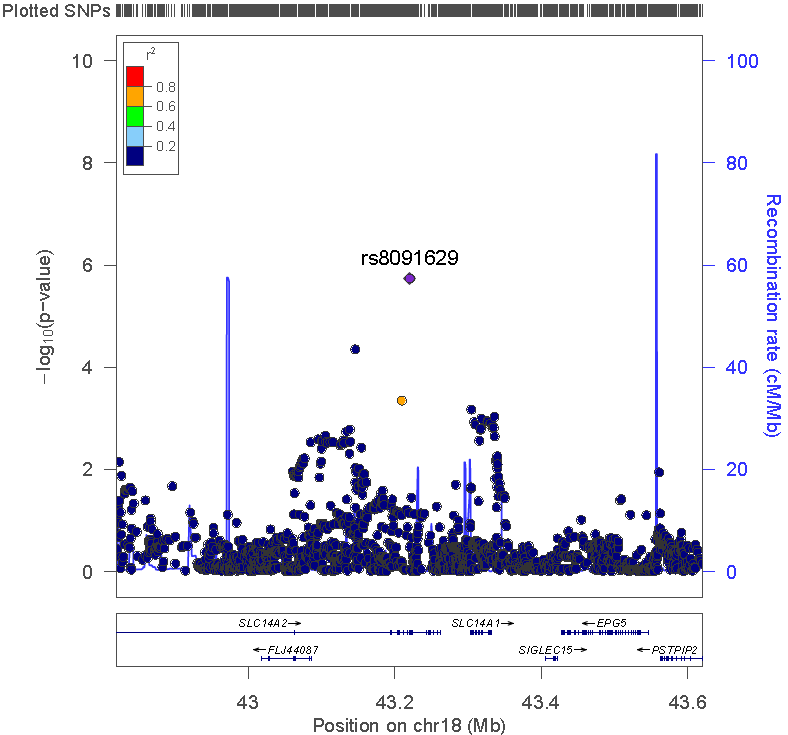** |
| Supplemental Figure 5e) Controls vs. multiple domains with substantial relative impairment |
| **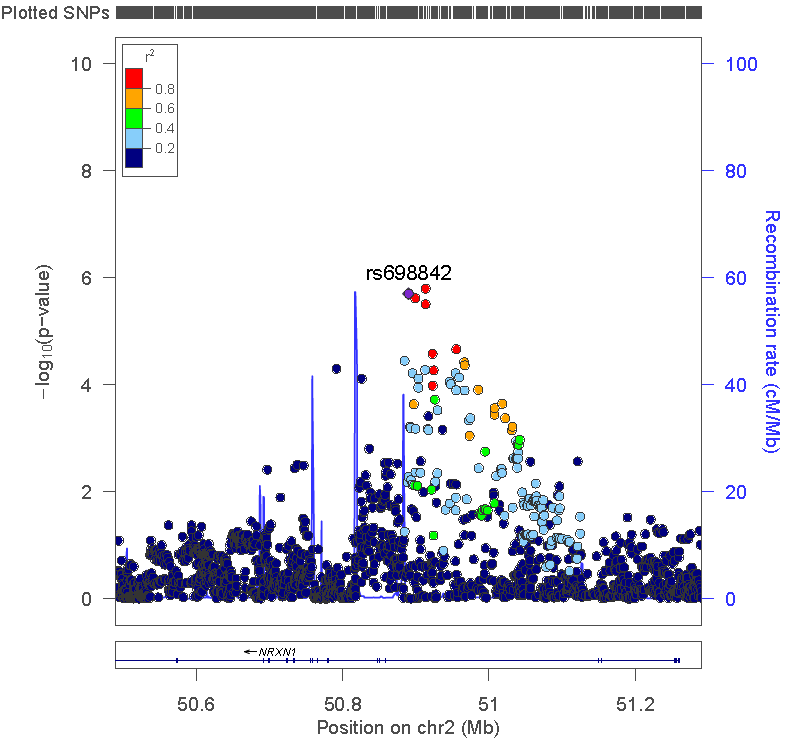** |

| **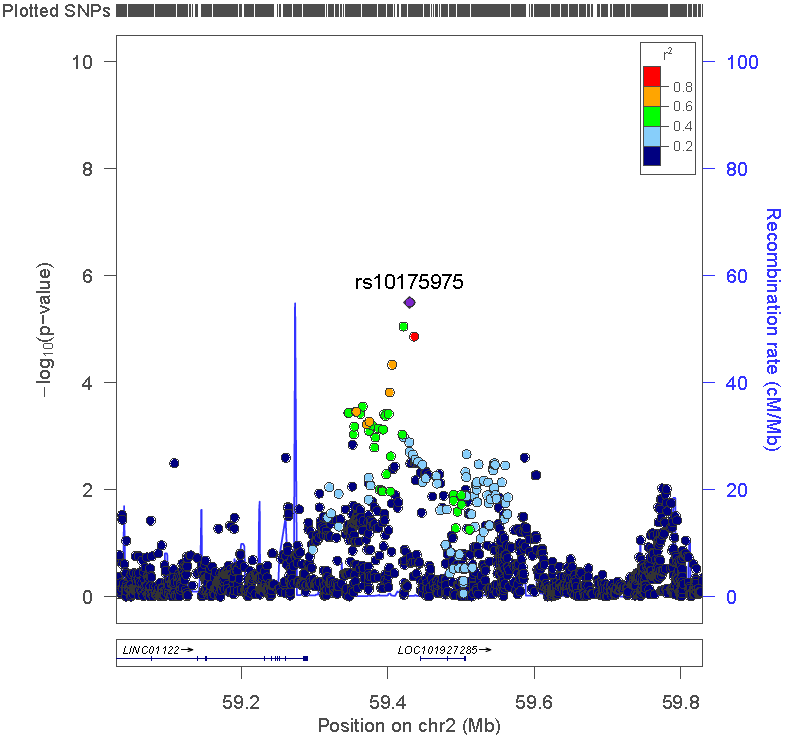** |
| --- |
| **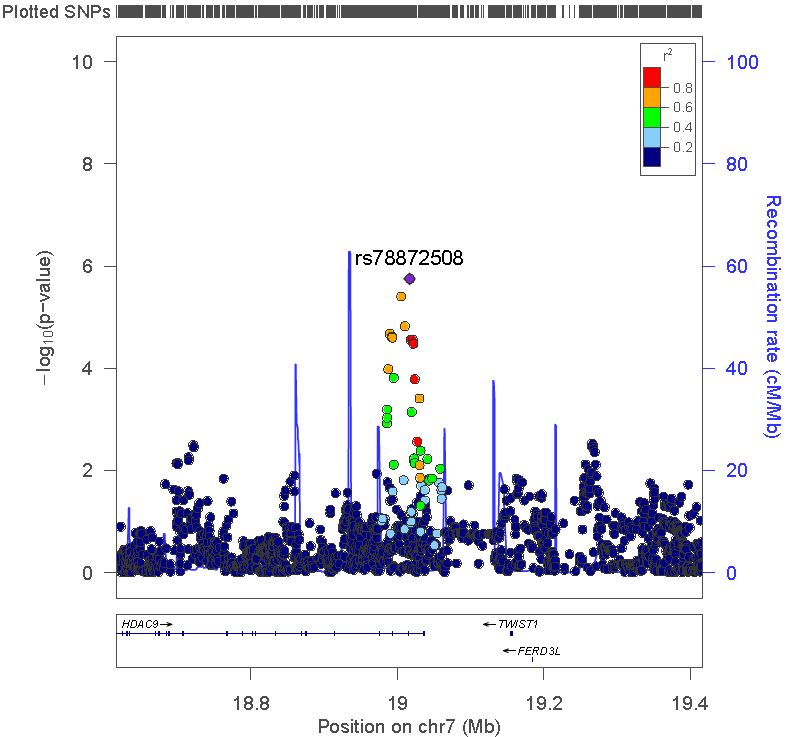** |
| **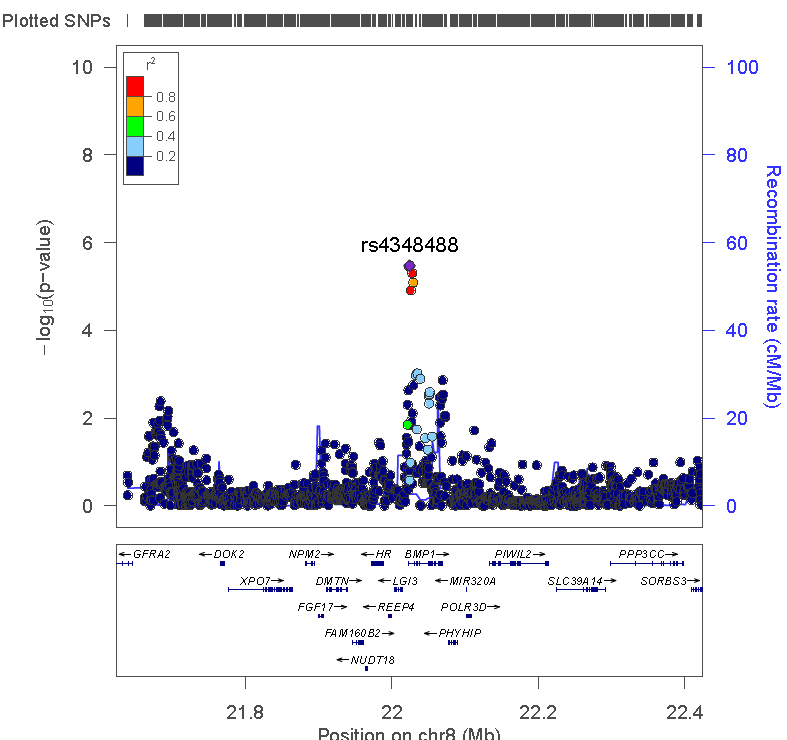** |
| **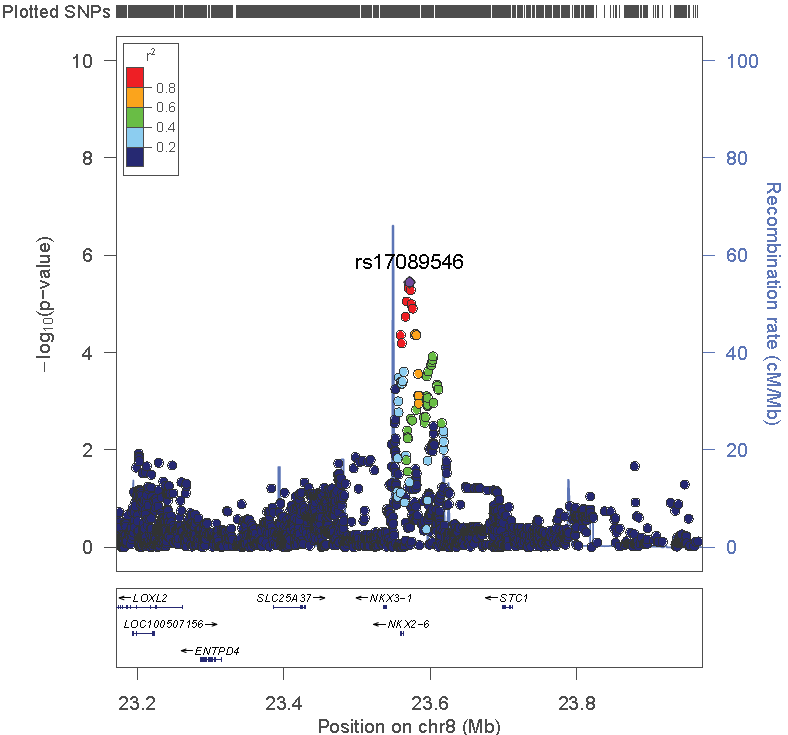** |
| **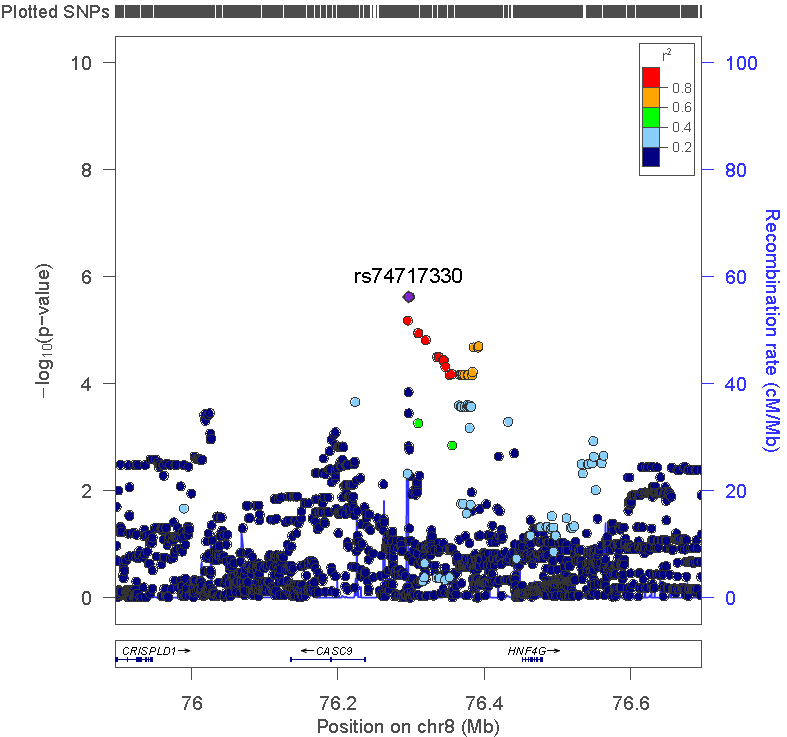** |
| **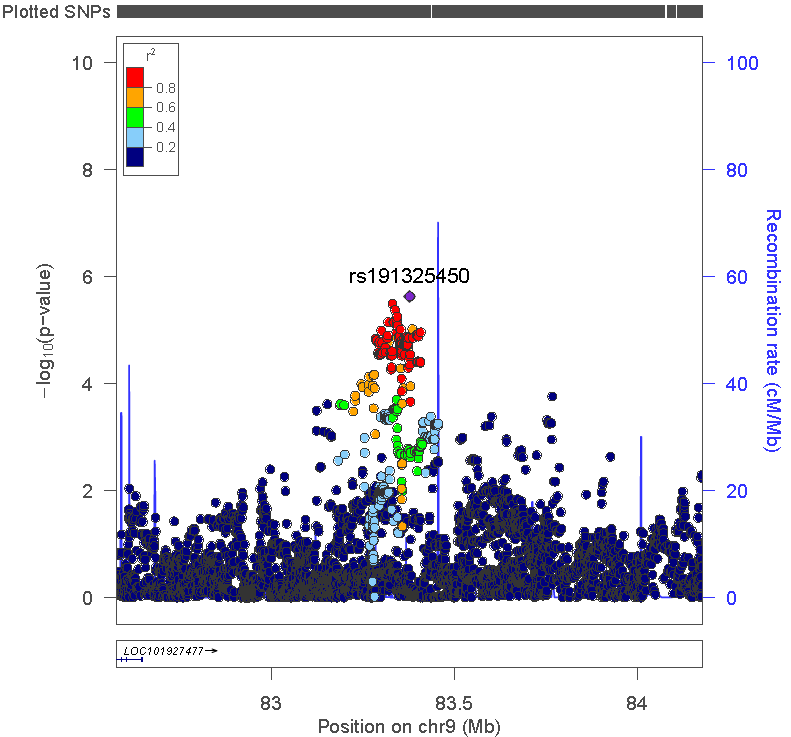** |
| **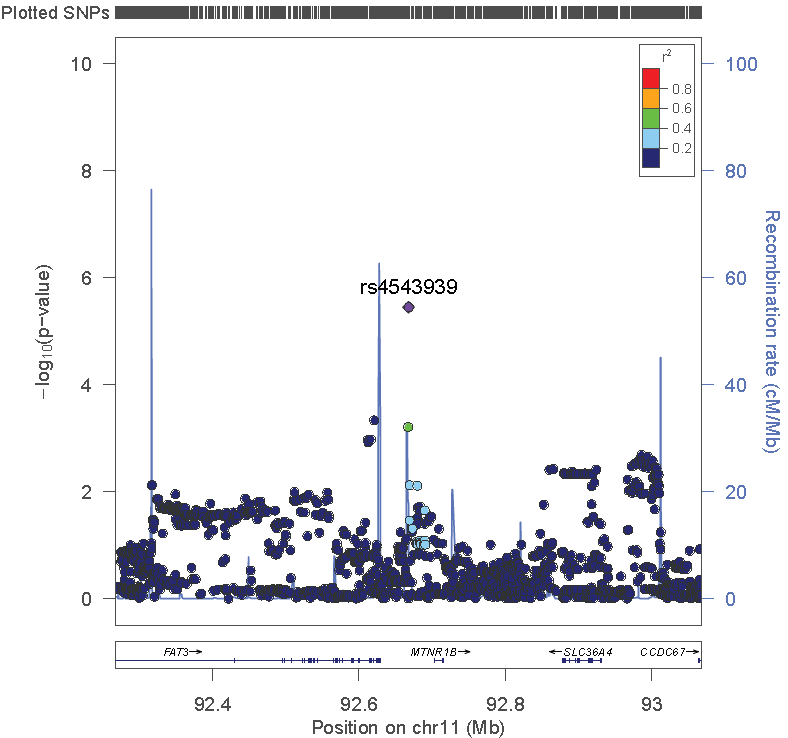** |
| **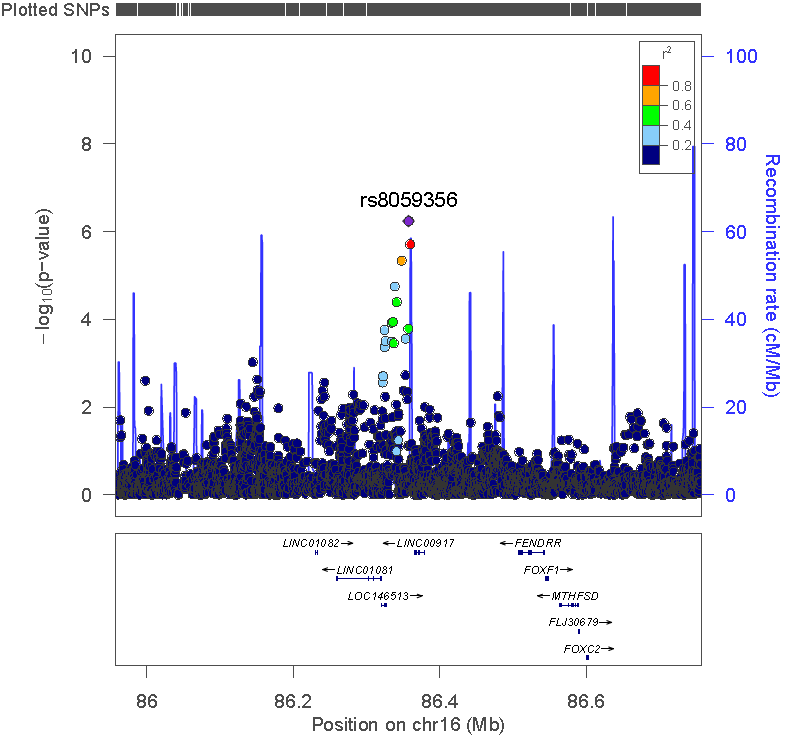** |

Supplemental Text 7. Genetic results: Memory SNPs

Supplemental Table 21a: Meta-analysis results for memory for memory SNPs

| SNP Characteristics | | | | | Meta analysis results | | | | |
| --- | --- | --- | --- | --- | --- | --- | --- | --- | --- |
| Chromosome | SNP | Base pair | Allele 1 | Allele 2 | Called allele frequency | OR | SE | P value | Heterogeneity p value |
| 1 | rs1977412 | 230852269 | T | C | 0.86 | 0.64 | 0.09 | 6.32E-07 | 0.29 |
| 16 | rs9937469 | 9252656 | T | C | 0.06 | 2.14 | 0.16 | 2.20E-06 | 0.10 |

Supplemental Table 21b: Study-specific results for memory SNPs

|  | ACT | | | ADNI | | | ROS-MAP | | | PITT | | |
| --- | --- | --- | --- | --- | --- | --- | --- | --- | --- | --- | --- | --- |
| SNP | OR | SE | P value | OR | SE | P value | OR | SE | P value | OR | SE | P value |
| rs1977412 | 0.48 | 0.22 | 7.40E-04 | 0.73 | 0.19 | 0.08 | 0.81 | 0.21 | 0.31 | 0.61 | 0.14 | 3.00E-04 |
| rs9937469 | 3.74 | 0.32 | 4.00E-04 | * | * | * | 2.16 | 0.33 | 0.02 | 1.62 | 0.22 | 0.03 |

* Indicates the analysis was missing for that study (ADNI did not have data for rs9937469).

Supplemental Table 21c: Meta-analysis for other subgroups for memory SNPs

|  | Isolated relative visuospatial impairment | | Isolated relative language impairment | | Multiple domains with relative impairments | | No domain with relative impairment | |
| --- | --- | --- | --- | --- | --- | --- | --- | --- |
| SNP | OR | P value | OR | P value | OR | P value | OR | P value |
| rs1977412 | 0.88 | 0.34 | 0.92 | 0.49 | 1.05 | 0.81* | 0.85 | 0.04 |
| rs9937469 | 1.62 | 0.05 | 1.45 | 0.12 | 1.23 | 0.56 | 1.02 | 0.91 |

* Indicates that the heterogeneity p value was <0.05 for that analysis

Supplemental Text 8. Genetic results: visuospatial SNPs

Supplemental Table 22a: Meta-analysis results for visuospatial for visuospatial SNPs

| SNP Characteristics | | | | | Meta analysis results | | | | |
| --- | --- | --- | --- | --- | --- | --- | --- | --- | --- |
| Chromosome | SNP | Base pair | Allele 1 | Allele 2 | Called allele frequency | OR | SE | P value | Heterogeneity p value |
| 1 | rs2795228 | 83941294 | A | T | 0.82 | 0.59 | 0.11 | 6.28E-07 | 0.17 |
| 1 | rs484947 | 229165488 | A | C | 0.62 | 0.64 | 0.10 | 2.03E-06 | 0.85 |
| 2 | rs16839220 | 156403868 | C | G | 0.20 | 0.53 | 0.14 | 4.57E-06 | 0.85 |
| 3 | rs2289506 | 100064902 | T | C | 0.34 | 1.61 | 0.09 | 3.39E-07 | 0.62 |
| 6 | rs9369477 | 44308629 | T | C | 0.92 | 0.49 | 0.14 | 1.62E-07 | 0.22 |
| 6 | rs9372110 | 106317196 | A | G | 0.06 | 2.16 | 0.15 | 1.73E-07 | 0.16 |
| 8 | rs2046197 | 3619752 | C | G | 0.59 | 1.66 | 0.10 | 3.44E-07 | 0.37 |
| 14 | rs8021600 | 44170626 | C | G | 0.92 | 0.51 | 0.14 | 3.18E-06 | 0.23 |
| 18 | rs8091629 | 43220331 | A | G | 0.90 | 0.54 | 0.13 | 1.83E-06 | 0.24 |

Supplemental Table 22b: Study-specific results for visuospatial SNPs

|  | ACT | | | ADNI | | | ROS-MAP | | | PITT | | |
| --- | --- | --- | --- | --- | --- | --- | --- | --- | --- | --- | --- | --- |
| SNP | OR | SE | P value | OR | SE | P value | OR | SE | P value | OR | SE | P value |
| rs2795228 | 0.50 | 0.22 | 0.001 | 0.90 | 0.24 | 0.69 | 0.60 | 0.21 | 0.02 | 0.48 | 0.20 | 2.60E-04 |
| rs484947 | 0.73 | 0.20 | 0.12 | 0.57 | 0.20 | 0.005 | 0.65 | 0.18 | 0.02 | 0.61 | 0.18 | 0.007 |
| rs16839220 | 0.44 | 0.31 | 0.01 | 0.55 | 0.28 | 0.03 | 0.62 | 0.25 | 0.05 | 0.49 | 0.27 | 0.01 |
| rs2289506 | 1.46 | 0.20 | 0.05 | 1.82 | 0.20 | 0.002 | 1.38 | 0.18 | 0.08 | 1.80 | 0.17 | 6.26E-04 |
| rs9369477 | 0.64 | 0.30 | 0.14 | 0.67 | 0.28 | 0.15 | 0.46 | 0.26 | 0.002 | 0.34 | 0.25 | 1.80E-05 |
| rs9372110 | 2.29 | 0.29 | 0.004 | 1.11 | 0.38 | 0.80 | 3.13 | 0.27 | 2.70E-05 | 1.99 | 0.27 | 0.01 |
| rs2046197 | 1.28 | 0.20 | 0.23 | 2.14 | 0.21 | 2.60E-04 | 1.72 | 0.20 | 0.007 | 1.65 | 0.19 | 0.008 |
| rs8021600 | 0.35 | 0.29 | 3.30E-04 | 0.84 | 0.31 | 0.57 | 0.49 | 0.26 | 0.005 | 0.51 | 0.30 | 0.03 |
| rs8091629 | 0.53 | 0.27 | 0.02 | 0.56 | 0.26 | 0.03 | 0.38 | 0.25 | 7.00E-05 | 0.79 | 0.27 | 0.36 |

Supplemental Table 22c: Meta-analysis for other subgroups for visuospatial SNPs

|  | Isolated relative memory impairment | | Isolated relative language impairment | | Multiple domains with relative impairments | | No domain with relative impairment | |
| --- | --- | --- | --- | --- | --- | --- | --- | --- |
| SNP | OR | P value | OR | P value | OR | P value | OR | P value |
| rs2795228 | 0.91 | 0.28 | 0.81 | 0.06 | 0.96 | 0.79 | 0.94 | 0.38 |
| rs484947 | 0.94 | 0.38* | 0.80 | 0.02 | 0.80 | 0.10 | 0.93 | 0.21 |
| rs16839220 | 1.01 | 0.89 | 0.93 | 0.51 | 0.70 | 0.04 | 0.92 | 0.22 |
| rs2289506 | 1.16 | 0.04 | 1.17 | 0.09 | 1.20 | 0.18 | 1.06 | 0.32 |
| rs9369477 | 0.96 | 0.75 | 0.90 | 0.52 | 0.90 | 0.66 | 1.01 | 0.89 |
| rs9372110 | 0.95 | 0.74 | 1.19 | 0.35 | 1.48 | 0.08 | 1.11 | 0.34 |
| rs2046197 | 1.01 | 0.86* | 1.18 | 0.08 | 0.94 | 0.65 | 1.05 | 0.36 |
| rs8021600 | 0.84 | 0.19 | 0.92 | 0.66 | 1.01 | 0.98 | 0.91 | 0.37 |
| rs8091629 | 0.84 | 0.13 | 0.79 | 0.13 | 0.74 | 0.14 | 0.81 | 0.02 |

* Indicates that the heterogeneity p value was <0.05 for that analysis

Supplemental Text 9. Genetic results: Language SNPs

Supplemental Table 23a: Meta-analysis results for language for language SNPs

| SNP Characteristics | | | | | Meta analysis results | | | | |
| --- | --- | --- | --- | --- | --- | --- | --- | --- | --- |
| Chromosome | SNP | Base pair | Allele 1 | Allele 2 | Called allele frequency | OR | SE | P value | Heterogeneity p value |
| 1 | rs13374908 | 179749774 | A | G | 0.24 | 1.59 | 0.10 | 2.33E-06 | 0.18 |
| 2 | rs28715896 | 212560101 | C | G | 0.57 | 0.63 | 0.10 | 3.50E-06 | 0.44 |
| 3 | rs75337321 | 54584587 | T | C | 0.06 | 2.21 | 0.17 | 4.59E-06 | 0.09 |
| 4 | rs10222981 | 6761053 | T | G | 0.08 | 2.06 | 0.15 | 1.24E-06 | 0.31 |
| 10 | rs6183545453 | 2759796 | T | C | 0.94 | 0.46 | 0.16 | 7.71E-07 | 0.16 |
| 17 | rs365521 | 61022295 | A | G | 0.47 | 0.63 | 0.10 | 1.48E-06 | 0.84 |

Supplemental Table 23b: Study-specific results for language SNPs

|  | ACT | | | ADNI | | | ROS-MAP | | | PITT | | |
| --- | --- | --- | --- | --- | --- | --- | --- | --- | --- | --- | --- | --- |
| SNP | OR | SE | P value | OR | SE | P value | OR | SE | P value | OR | SE | P value |
| rs13374908 | 0.89 | 0.29 | 0.69 | 1.49 | 0.26 | 0.12 | 1.82 | 0.15 | 4.28E-05 | 1.67 | 0.19 | 0.007 |
| rs28715896 | 0.64 | 0.26 | 0.10 | 0.64 | 0.23 | 0.05 | 0.71 | 0.15 | 0.02 | 0.45 | 0.23 | 5.10E-04 |
| rs75337321 | 0.93 | 0.56 | 0.90 | 1.26 | 0.39 | 0.56 | 2.72 | 0.26 | 1.30E-04 | 3.25 | 0.33 | 4.22E-04 |
| rs10222981 | 2.53 | 0.31 | 0.003 | 2.97 | 0.36 | 0.003 | 1.36 | 0.28 | 0.27 | 2.10 | 0.26 | 0.005 |
| rs6183545453 | 0.26 | 0.32 | 2.76E-05 | 0.49 | 0.45 | 0.113 | 0.66 | 0.25 | 0.10 | 0.42 | 0.33 | 0.009 |
| rs365521 | 0.52 | 0.27 | 0.01 | 0.58 | 0.25 | 0.03 | 0.67 | 0.14 | 0.004 | 0.65 | 0.19 | 0.02 |

Supplemental Table 23c: Meta-analysis for other subgroups for language SNPs

|  | Isolated relative memory impairment | | Isolated relative visuospatial impairment | | Multiple domains with relative impairments | | No domain with relative impairment | |
| --- | --- | --- | --- | --- | --- | --- | --- | --- |
| SNP | OR | P value | OR | P value | OR | P value | OR | P value |
| rs13374908 | 1.15 | 0.07 | 1.03 | 0.76 | 1.08 | 0.63 | 1.16 | 0.03 |
| rs28715896 | 0.85 | 0.03 | 0.83 | 0.08 | 1.07 | 0.62 | 0.93 | 0.21 |
| rs75337321 | 1.03 | 0.85 | 1.09 | 0.68 | 1.13 | 0.69 | 0.99 | 0.95 |
| rs10222981 | 1.00 | 0.98 | 1.22 | 0.26 | 0.92 | 0.76 | 1.06 | 0.60 |
| rs6183545453 | 0.83 | 0.20 | 0.84 | 0.36 | 0.83 | 0.49 | 0.92 | 0.51 |
| rs365521 | 1.00 | 0.98 | 0.89 | 0.22 | 0.99 | 0.96* | 0.88 | 0.03 |

* Indicates the heterogeneity p value was <0.05 for that analysis

Supplemental Text 10. Genetic results: Multiple domains SNPs

Supplemental Table 24a: Meta-analysis results for the multiple domains group for multiple domains SNPs

| SNP Characteristics | | | | | Meta analysis results | | | | |
| --- | --- | --- | --- | --- | --- | --- | --- | --- | --- |
| Chromosome | SNP | Base pair | Allele 1 | Allele 2 | Called allele frequency | OR | SE | P value | Heterogeneity p value |
| 2 | rs698842 | 50890096 | A | T | 0.22 | 1.96 | 0.14 | 1.98E-06 | 0.11 |
| 2 | rs10175975 | 59429807 | T | C | 0.19 | 1.99 | 0.15 | 3.14E-06 | 0.31 |
| 7 | rs78872508 | 19016476 | T | C | 0.87 | 0.45 | 0.17 | 1.78E-06 | 0.98 |
| 8 | rs4348488 | 22024162 | C | G | 0.21 | 1.97 | 0.15 | 3.36E-06 | 0.96 |
| 8 | rs17089546 | 23571807 | A | G | 0.25 | 1.86 | 0.13 | 3.29E-06 | 0.76 |
| 8 | rs74717330 | 76296308 | A | C | 0.05 | 3.09 | 0.24 | 2.37E-06 | 0.97 |
| 9 | rs191325450 | 83377984 | A | G | 0.91 | 0.43 | 0.18 | 2.32E-06 | 0.93 |
| 11 | rs4543939 | 92667658 | A | T | 0.43 | 2.43 | 0.19 | 3.54E-06 | 0.33 |
| 16 | rs8059356 | 86357245 | A | G | 0.21 | 2.23 | 0.16 | 5.75E-07 | 0.76 |

Supplemental Table 24b: Study-specific results for multiple domain SNPs

|  | ACT | | | ADNI | | | ROS-MAP | | | PITT | | |
| --- | --- | --- | --- | --- | --- | --- | --- | --- | --- | --- | --- | --- |
| SNP | OR | SE | P value | OR | SE | P value | OR | SE | P value | OR | SE | P value |
| rs698842 | * | * | * | 1.15 | 0.36 | 0.70 | 1.80 | 0.20 | 0.003 | 2.75 | 0.24 | 1.91E-05 |
| rs10175975 | * | * | * | 2.94 | 0.33 | 0.001 | 2.03 | 0.22 | 0.001 | 1.55 | 0.25 | 0.08 |
| rs78872508 | * | * | * | 0.47 | 0.39 | 0.06 | 0.45 | 0.24 | 0.001 | 0.43 | 0.29 | 0.004 |
| rs4348488 | * | * | * | 1.90 | 0.34 | 0.06 | 1.92 | 0.22 | 0.003 | 2.08 | 0.24 | 0.002 |
| rs17089546 | * | * | * | 1.99 | 0.29 | 0.02 | 1.65 | 0.21 | 0.02 | 2.05 | 0.22 | 0.001 |
| rs74717330 | * | * | * | * | * | * | 3.06 | 0.32 | 5.35E-04 | 3.13 | 0.35 | 0.001 |
| rs191325450 | * | * | * | 0.38 | 0.39 | 0.01 | 0.43 | 0.26 | 0.001 | 0.45 | 0.33 | 0.02 |
| rs4543939 | * | * | * | * | * | * | 2.83 | 0.24 | 2.15E-05 | 1.92 | 0.31 | 0.04 |
| rs8059356 | * | * | * | * | * | * | 2.34 | 0.22 | 1.14E-04 | 2.12 | 0.23 | 0.001 |

* Indicates that data were not available for that SNP in that study. There were too few people from ACT in this group to include in meta-analyses. ADNI was missing data for three SNPs.

Supplemental Table 24c: Meta-analysis for other subgroups for multiple domain SNPs

|  | Isolated relative memory impairment | | Isolated relative visuospatial impairment | | Isolated relative language impairment | | No domain with relative impairment | |
| --- | --- | --- | --- | --- | --- | --- | --- | --- |
| SNP | OR | P value | OR | P value | OR | P value | OR | P value |
| rs698842 | 1.13 | 0.13 | 1.11 | 0.34 | 1.37 | ??? | 1.01 | 0.93 |
| rs10175975 | 1.01 | 0.94 | 1.07 | 0.59 | 1.11 | 0.36 | 0.99 | 0.84 |
| rs78872508 | 0.80 | 0.02 | 0.84 | 0.22 | 0.81 | 0.11 | 0.88 | 0.14 |
| rs4348488 | 1.11 | 0.22 | 1.05 | 0.68 | 1.20 | 0.10 | 1.06 | 0.43 |
| rs17089546 | 0.93 | 0.38 | 1.03 | 0.78 | 1.26 | 0.02 | 1.04 | 0.50 |
| rs74717330 | 1.14 | 0.48* | 1.24 | 0.33 | 0.94 | 0.79 | 1.10 | 0.48 |
| rs191325450 | 0.77 | 0.02 | 0.85 | 0.30 | 0.92 | 0.61 | 0.99 | 0.89 |
| rs4543939 | 0.96 | 0.66 | 0.97 | 0.85 | 0.96 | 0.76 | 1.01 | 0.87 |
| rs8059356 | 1.17 | 0.12 | 1.17 | 0.26 | 0.77 | 0.07 | 1.19 | 0.05 |

* Indicates the heterogeneity p value was <0.05 for that analysis.

Supplemental Text 11. Genetic results: No domain with a substantial relative impairment SNPs

Supplemental Table 25a: Meta-analysis results for the no domain group for no domain with a substantial relative impairment SNPs

| SNP Characteristics | | | | | Meta analysis results | | | | |
| --- | --- | --- | --- | --- | --- | --- | --- | --- | --- |
| Chromosome | SNP | Base pair | Allele 1 | Allele 2 | Called allele frequency | OR | SE | P value | Heterogeneity p value |
| 2 | rs4972634 | 174907205 | T | C | 0.59 | 1.31 | 0.06 | 3.84E-06 | 0.61 |
| 3 | rs11708767 | 151119726 | A | G | 0.43 | 1.33 | 0.06 | 2.82E-07 | 0.76 |
| 6 | rs4533991 | 51098648 | T | G | 0.46 | 1.32 | 0.06 | 1.55E-06 | 0.91 |
| 6 | rs78358979 | 87504903 | A | T | 0.06 | 1.76 | 0.11 | 4.95E-07 | 0.15 |
| 7 | rs6978679 | 105718743 | A | G | 0.73 | 1.36 | 0.06 | 2.02E-06 | 0.44 |
| 17 | rs72839770 | 7132192 | T | C | 0.36 | 1.31 | 0.06 | 4.12E-06 | 0.61 |
| 20 | rs7264688 | 17954706 | T | G | 0.67 | 1.47 | 0.08 | 4.70E-06 | 0.85 |

Supplemental Table 25b: Study-specific results for no domain with a substantial relative impairment SNPs

|  | ACT | | | ADNI | | | ROS-MAP | | | PITT | | |
| --- | --- | --- | --- | --- | --- | --- | --- | --- | --- | --- | --- | --- |
| SNP | OR | SE | P value | OR | SE | P value | OR | SE | P value | OR | SE | P value |
| rs4972634 | 1.28 | 0.11 | 0.02 | 1.27 | 0.13 | 0.06 | 1.21 | 0.12 | 0.12 | 1.49 | 0.12 | 6.29E-04 |
| rs11708767 | 1.43 | 0.11 | 0.001 | 1.39 | 0.13 | 0.009 | 1.31 | 0.11 | 0.01 | 1.22 | 0.11 | 0.06 |
| rs4533991 | 1.39 | 0.11 | 0.004 | 1.31 | 0.12 | 0.03 | 1.36 | 0.12 | 0.008 | 1.25 | 0.11 | 0.06 |
| rs78358979 | 1.82 | 0.18 | 0.001 | 2.69 | 0.24 | 3.35E-05 | 1.32 | 0.25 | 0.25 | 1.39 | 0.26 | 0.21 |
| rs6978679 | 1.51 | 0.13 | 0.001 | 1.52 | 0.14 | 0.003 | 1.23 | 0.13 | 0.10 | 1.23 | 0.12 | 0.08 |
| rs72839770 | 1.34 | 0.11 | 0.006 | 1.16 | 0.12 | 0.22 | 1.28 | 0.14 | 0.08 | 1.49 | 0.11 | 0.001 |
| rs7264688 | 1.63 | 0.19 | 0.01 | 1.48 | 0.13 | 0.003 | 1.55 | 0.20 | 0.03 | 1.30 | 0.18 | 0.16 |

Supplemental Table 25c: Meta-analysis for other subgroups for no domain with a substantial relative impairment SNPs

|  | Isolated relative memory impairment | | Isolated relative visuospatial impairment | | Isolated relative language impairment | | Multiple domains with relative impairments | |
| --- | --- | --- | --- | --- | --- | --- | --- | --- |
| SNP | OR | P value | OR | P value | OR | P value | OR | P value |
| rs4972634 | 1.09 | 0.22 | 1.06 | 0.54 | 0.95 | 0.56 | 0.98 | 0.86 |
| rs11708767 | 1.11 | 0.12 | 1.04 | 0.70 | 1.08 | 0.37 | 0.96 | 0.73 |
| rs4533991 | 1.20 | 0.01 | 1.15 | 0.14 | 0.99 | 0.89 | 0.93 | 0.57 |
| rs78358979 | 1.09 | 0.60 | 1.10 | 0.64 | 0.95 | 0.82 | 1.94 | 0.01 |
| rs6978679 | 1.05 | 0.51 | 1.03 | 0.77 | 1.06 | 0.58 | 0.94 | 0.67 |
| rs72839770 | 0.94 | 0.40 | 1.12 | 0.25 | 1.03 | 0.76 | 0.97 | 0.82 |
| rs7264688 | 1.19 | 0.07 | 1.06 | 0.69 | 1.26 | 0.08 | 1.58 | 0.02 |

**Supplemental Text 12. Generation of genetic risk scores and their ability to predict Alzheimer’s disease case/control status**

We used information on SNPs to construct gene scores for each of the five Alzheimer’s disease subgroups and Alzheimer’s disease gene scores based on the Lambert *et al.* IGAP GWAS of late onset Alzheimer’s disease paper^17^.

For the subgroup gene scores we used SNPs which had a minor allele frequency > 3%, a *p*-value < 10^-5^ for the meta-analysis of one of the subgroups, and a heterogeneity *p*-value > 0.05. We checked for linkage disequilibrium (LD) between these SNPs and found that these SNPs were not in LD.

In total, we made eight different gene scores in our combined four-cohort data set (n=5,878):

1) Two SNPs were used to derive a gene score for the isolated relative memory impairment subgroup (grs_mem).

2) Nine SNPs were used to derive a gene score for the isolated relative visuospatial impairment subgroup (grs_vsp).

3) Six SNPs were used to derive a gene score for the isolated relative language impairment subgroup (grs_lan).

4) Nine SNPs were used to derive a gene score for the multiple domains with substantial relative impairments subgroup (grs_mix).

5) Seven SNPs were used to derive a gene score for the subgroup with no domain with a substantial relative impairment (grs_none).

6) Nineteen SNPs were used to derive an Alzheimer’s disease risk score based on effect sizes reported in the IGAP Lambert et al. paper (grs_igap_lambert).

7) The same 19 IGAP SNPs were used to derive an Alzheimer’s disease risk score (grs_igap_cc) based on beta coefficients from an analysis of the Alzheimer’s disease case-control phenotype from the four cohorts we analyzed (ACT/ADNI/ROS-MAP/PITT).

8) We performed a GWAS of the Alzheimer’s disease case-control phenotype from our data and identified 16 SNPs with a *p*-value < 10^-5^ from the four cohorts we analyzed (ACT/ADNI/ROS-MAP/PITT). We used these SNPs to derive an EPAD-specific Alzheimer’s disease risk score (grs_epad_cc).

Details about SNPs, risk alleles, and effect sizes used to derive each of the gene scores are given at the end of the appendix.

The SNPs and the associated effect estimates were combined in a gene score assuming an additive genetic model where $\beta_{k}$ is the corresponding SNP weight describing the effect of an additional risk allele on the phenotype.

$$\mathrm{GRS}_{i}=\sum_{k=1}^{\# loci} \beta_{k} \cdot{allele count}_{i, k}$$

PLINK^22^ was used to derive the gene scores.

We used the gene scores to predict Alzheimer’s disease in logistic regression models.

We ran the following regressions in our combined four cohort data set with 3,447 cognitively normal elderly controls and 2,431 people with Alzheimer’s disease using Stata:

A) Logistic regression for Alzheimer’s disease case-control status adjusting for age, sex, and the five Alzheimer’s disease subtype genetic risk scores (grs_mem, grs_vsp, grs_lan, grs_mix, and grs_none)

B) Logistic regression for Alzheimer’s disease case-control status adjusting for age, sex, the five AD subtype genetic risk scores, and grs_igap_lambert

C) Logistic regression for Alzheimer’s disease case-control status adjusting for age, sex, and grs_igap_lambert

D) Logistic regression for Alzheimer’s disease case-control status adjusting for age, sex, and grs_igap_cc

E) Logistic regression for Alzheimer’s disease case-control status adjusting for age, sex, and grs_epad_cc

**Analysis I.** Under a nested design setting, we test Model B vs. Model C where Model B is the full model. We obtained χ^2^_df=5_ = 135.8, p=1.4 × 10^-27^, which means that the variables that was removed to produce the reduced model resulted in a model that has a significantly poorer fit, and therefore the variable should be included in the model. This implies that the five subtype GRSs have additional power to predict Alzheimer’s disease.

**Analysis II.** We, also, test the difference in area under receiver operator characteristic (ROC) curve for the following set of non-nested models:

i) Model A vs. Model C

ii) Model A vs. Model D

iii) Model A vs. Model E

Supplemental Table 26. Area under the receiver operator characteristic curve for predicting late-onset Alzheimer’s disease case-control status with different groups of SNPs

|  | Model A | Model C | Model D | Model E |
| --- | --- | --- | --- | --- |
| Area under ROC curve | 0.621  SE = 0.01 | 0.598  SE = 0.01 | 0.582  SE = 0.01 | 0.584  SE = 0.01 |

* SE = Standard error

We then test equality of ROC areas between Model A and Models C, D, and E.

Supplemental Table 27. Tests of equality of receiver operator characteristic curves compared with that produced from IGAP SNPs (compared with Model A)

| Model C | Model D | Model E |
| --- | --- | --- |
| χ^2^_df=1_ = 11.2,  p=0.0008 | χ^2^_df=1_ = 41.3,  p=1.3 × 10^-10^ | χ^2^_df=1_ = 34.8,  p=3.6 × 10^-9^ |

Model A, with the five subtype GRSs, was significantly better at predicting Alzheimer’s disease than the alternative models that used scores based only on Alzheimer’s disease case/control status.

**Supplemental Table 28. SNPs used to calculate gene scores**

| **SNPs** | **Chromosome** | **Risk Allele** | **Risk Allele Frequency** | **Beta** |
| --- | --- | --- | --- | --- |
| 1) Isolated relative memory impairment subtype | | | | |
| rs1977412 | 1 | T | 0.86 | -0.44 |
| rs9937469* | 16 | T | 0.06 | 0.76 |
| 2) Isolated relative visuospatial ability impairment subtype | | | | |
| rs2795228 | 1 | A | 0.82 | -0.54 |
| rs484947 | 1 | A | 0.62 | -0.45 |
| rs16839220 | 2 | C | 0.2 | -0.63 |
| rs2289506 | 3 | T | 0.34 | 0.48 |
| rs9369477 | 6 | T | 0.92 | -0.71 |
| rs9372110 | 6 | A | 0.06 | 0.77 |
| rs2046197 | 8 | C | 0.59 | 0.51 |
| rs8021600 | 14 | C | 0.92 | -0.67 |
| rs8091629 | 18 | A | 0.9 | -0.63 |
| 3) Isolated relative language impairment subtype | | | | |
| rs13374908 | 1 | A | 0.24 | 0.46 |
| rs28715896 | 2 | C | 0.57 | -0.46 |
| rs75337321 | 3 | T | 0.06 | 0.79 |
| rs10222981 | 4 | T | 0.08 | 0.72 |
| rs61835453 | 10 | T | 0.94 | -0.78 |
| rs365521 | 17 | A | 0.47 | -0.46 |
| 4) Multiple domains with substantial relative impairment | | | | |
| rs698842 | 2 | A | 0.22 | 0.67 |
| rs10175975 | 2 | T | 0.19 | 0.69 |
| rs78872508 | 7 | T | 0.87 | -0.8 |
| rs4348488 | 8 | C | 0.21 | 0.68 |
| rs17089546 | 8 | A | 0.25 | 0.62 |
| rs74717330* | 8 | A | 0.05 | 1.13 |
| rs191325450 | 9 | A | 0.91 | -0.86 |
| rs4543939* | 11 | A | 0.43 | 0.89 |
| rs8059356* | 16 | A | 0.21 | 0.8 |
| 5) No domain with a substantial relative impairment | | | | |
| rs4972634 | 2 | T | 0.59 | 0.27 |
| rs11708767 | 3 | A | 0.43 | 0.29 |
| rs4533991 | 6 | T | 0.46 | 0.28 |
| rs78358979 | 6 | A | 0.06 | 0.57 |
| rs6978679 | 7 | A | 0.73 | 0.3 |
| rs72839770 | 17 | T | 0.36 | 0.27 |
| rs7264688 | 20 | T | 0.67 | 0.39 |
| 6) IGAP Alzheimer’s disease gene score based on Lambert *et al.* paper | | | | |
| rs6656401 | 1 | A | 0.20 | 0.17 |
| rs6733839 | 2 | T | 0.41 | 0.20 |
| rs10948363 | 6 | G | 0.27 | 0.10 |
| rs11771145 | 7 | A | 0.34 | -0.11 |
| rs9331896 | 8 | C | 0.38 | -0.15 |
| rs983392 | 11 | G | 0.40 | -0.11 |
| rs10792832 | 11 | A | 0.36 | -0.14 |
| rs4147929 | 19 | A | 0.19 | 0.14 |
| rs9271192* | 6 | C | 0.28 | 0.10 |
| rs28834970 | 8 | C | 0.37 | 0.10 |
| rs11218343* | 11 | C | 0.04 | -0.26 |
| rs10498633 | 14 | T | 0.22 | -0.09 |
| rs35349669 | 2 | T | 0.49 | 0.08 |
| rs190982 | 5 | G | 0.41 | -0.07 |
| rs2718058 | 7 | G | 0.37 | -0.07 |
| rs1476679 | 7 | C | 0.29 | -0.09 |
| rs10838725 | 11 | C | 0.32 | 0.08 |
| rs17125944 | 14 | C | 0.09 | 0.13 |
| rs7274581 | 20 | C | 0.08 | -0.13 |
| 7) IGAP Alzheimer’s disease gene score based on case-control meta-analysis of the four EPAD cohorts. | | | | |
| rs6656401^†^ | 1 | A | 0.19 | 0.19 |
| rs6733839 | 2 | T | 0.39 | 0.17 |
| rs10948363 | 6 | G | 0.37 | 0.11 |
| rs11771145 | 7 | A | 0.34 | 0.004 |
| rs9331896 | 8 | C | 0.38 | -0.06 |
| rs983392 | 11 | G | 0.40 | -0.10 |
| rs10792832 | 11 | A | 0.36 | -0.13 |
| rs4147929 | 19 | A | 0.17 | 0.18 |
| rs9271192* | 6 | C | 0.27 | 0.05 |
| rs28834970 | 8 | C | 0.36 | 0.11 |
| rs11218343* | 11 | C | 0.04 | -0.24 |
| rs10498633 | 14 | T | 0.22 | -0.04 |
| rs35349669 | 2 | T | 0.48 | 0.06 |
| rs190982 | 5 | G | 0.48 | -0.15 |
| rs2718058 | 7 | G | 0.36 | -0.05 |
| rs1476679 | 7 | C | 0.38 | -0.10 |
| rs10838725 | 11 | C | 0.31 | 0.05 |
| rs17125944 | 14 | C | 0.09 | 0.11 |
| rs7274581 | 20 | C | 0.09 | -0.14 |
| 8) EPAD-specific Alzheimer’s disease risk score with SNPs p < 10^-5^ in case-control meta-analysis of the four EPAD cohorts. | | | | |
| rs72634809 | 1 | T | 0.26 | 0.78 |
| rs6540721 | 1 | A | 0.16 | -0.28 |
| rs11922676 | 3 | C | 0.92 | -0.67 |
| rs4857132 | 3 | A | 0.77 | -0.27 |
| rs13434494 | 4 | T | 0.86 | -0.27 |
| rs6827227 | 4 | T | 0.46 | -0.19 |
| rs537483 | 5 | A | 0.51 | 0.19 |
| rs78077027 | 5 | T | 0.03 | 1.02 |
| rs6998234 | 8 | T | 0.05 | 0.42 |
| rs28641873 | 8 | A | 0.08 | 0.48 |
| rs7929347 | 11 | A | 0.61 | -0.19 |
| rs6589973 | 11 | A | 0.30 | 0.20 |
| rs3751239 | 12 | C | 0.80 | 0.23 |
| rs26833 | 16 | A | 0.40 | -0.20 |
| rs9939163 | 16 | T | 0.80 | 0.26 |
| rs844912 | 20 | T | 0.18 | 0.25 |

* Missing in ADNI genetic data set;

^†^ Heterogeneity *p*-value significant in meta-analysis;

Risk allele frequency corresponds to the frequency estimated in the given meta-analysis.

**Supplemental text 8. Information regarding the Sweet et al. analyses**

Sweet and colleagues^23^ present data in terms of the sum of beta coefficients for two samples; the relevant comparison between our Figure 2 and their Table 4 would be their data divided by 2 to derive the average beta coefficient in each study. These ranged from +0.15 to ‑0.18, corresponding to ORs of 0.84 to 1.16, similar in magnitude to those previously reported for Alzheimer’s disease from IGAP^24^.

**Supplemental Table 29. Demographic and cognitive characteristics by subgroup. N (%) or mean (SD).**

|  | No Domain  (n=1584) | Memory  (n=1107) | Executive  (n=104) | Language  (n=510) | Visuospatial  (n=497) | Multiple  (n=248) |
| --- | --- | --- | --- | --- | --- | --- |
| Female | 977 (62%) | 679 (61%) | 53 (51%) | 309 (61%) | 314 (63%) | 150 (60%) |
| Age at diagnosis | 81 (8) | 79 (8) | 81 (9) | 82 (8) | 79 (9) | 81 (8) |
| Education | 14 (3) | 14 (3) | 14 (4) | 15 (3) | 14 (3) | 14 (3) |
| White | 1467 (93%) | 1019 (92%) | 97 (93%) | 471 (92%) | 434 (87%) | 231 (93%) |
| Memory | -0.1 (0.9) | -1.0 (0.7) | 0.6 (1.0) | 0.0 (1.0) | 0.3 (1.1) | -0.8 (1.4) |
| Visuospatial | 0.1 (1.0) | 1.0 (1.0) | 0.6 (1.2) | 0.6 (1.1) | -1.4 (1.0) | 0.6 (1.6) |
| Executive function | 0.2 (0.9) | 0.8 (0.8) | -0.9 (0.9) | 0.2 (1.0) | 0.3 (1.1) | 0.5 (1.3) |
| Language | 0.0 (0.9) | 0.4 (0.9) | 0.2 (1.0) | -1.4 (1.0) | 0.2 (1.1) | -0.7 (1.5) |

Supplemental Table 30. IGAP SNPs with OR>1.30 or <0.77 in one subgroup and for which results from all four datasets were in the same direction

| **IGAP^a^** | | |  | **Results for notable subgroup^b^** | | | | | | | **Meta-analysis results for other subgroups**^e^ | | | | |
| --- | --- | --- | --- | --- | --- | --- | --- | --- | --- | --- | --- | --- | --- | --- | --- |
|  |  |  |  | **Meta-analysis results^c^** | | | **Single study results^d^** | | | |  | | |  |  |
| **SNP** | **Gene** | **OR** | **Notable Subgroup** | **OR** | **p vs. null** | **p vs. IGAP** | **ACT** | **ADNI** | **ROS-MAP** | **PITT** | **No domain** | **Memory** | **Language** | **Visuo-spatial** | **Multiple domains** |
| rs28834970 | *PTK2B* | 1.10 | Multiple domains | 1.51 | 0.0018 | 0.017 | 1.65 | 1.65 | 1.45 | 1.52 | 1.09 | 1.08 | 1.15 | 0.92 | ** |
| rs190982 | *MEF2C* | 0.93 | Visuospatial | 0.73 | 0.0021 | 0.019 | 0.75 | 0.99 | 0.60 | 0.64 | 0.85 | 0.85 | 0.86 | ** | 1.25 |
| rs11218343 | *SORL1* | 0.77 | Visuospatial | 0.61 | 0.12 | 0.47 | 0.56 | n/a | 0.84 | 0.41 | 0.89 | 0.73 | 1.02 | ** | 0.83 |
| rs7274581 | *CASS4* | 0.88 | Memory | 0.71 | 0.0058 | 0.09 | 0.83 | 0.55 | 0.84 | 0.72 | 0.86 | ** | 0.90 | 0.98 | 0.88 |
| rs17125944 | *FERMT2* | 1.14 | Memory | 1.37 | 0.0027 | 0.09 | 1.28 | 1.48 | 1.31 | 1.38 | 1.11 | ** | 0.88 | 1.03 | 0.96 |
| rs6656401 | *CR1* | 1.18 | Visuospatial | 1.42 | 0.0014 | 0.09 | 1.52 | 1.83 | 1.31 | 1.18 | 1.13 | 1.21^f^ | 1.33 | ** | 1.11 |

^a^ The odds ratios presented here are those reported in Lambert et al.^24^

^b^ These columns present data for the subgroup with an OR >1.30 or <0.77.

^c^ These columns present results from the meta-analysis results combining all studies, though for the subgroup with multiple domains with substantial relative impairments, ACT was excluded due to having only 12 people in that subgroup. The “Fold” column is the ratio of the meta-analytic odds ratio to that reported in Lambert et al. The “p vs. null” column shows the p value for the comparison of the subgroup to cognitively normal elderly controls, and the “p vs. IGAP” column shows the p value for the comparison of the subgroup to the values reported in Lambert et al., using the same method as in^25^. We evaluated 19 SNPs; the Bonferroni-corrected critical value is 0.05/19 = 0.0026.

^d^ These columns show results for the notable subgroup from each study separately. The rs11218343 SNP was not reported for ADNI.

^e^ These columns show results for other subgroups. The double asterisk (**) indicates that subgroup was the notable subgroup with results presented to the left.

^f^ Meta-analysis odds ratios did not show statistical evidence of heterogeneity across studies except for the group with isolated substantial relative memory impairment, where rs665401 associated with *CR1* had a p value of 0.007.

**Supplemental Table 31. Data for all IGAP SNPs for all of the studies and all the subgroups**

| **Grp** | **SNP** | **Gene** | **IGAP OR** | **Meta** | **p** | **Heter. p** | **Meta vs IGAP**  **p-value** | **ACT** | **p** | **ADNI** | **p** | **Rush** | **p** | **Pitt** | **p** |
| --- | --- | --- | --- | --- | --- | --- | --- | --- | --- | --- | --- | --- | --- | --- | --- |
| none | rs6733839 | *BIN1* | 1.22 | 1.17 | 0.01 | 0.76 | 0.52 | 1.22 | 0.09 | 1.18 | 0.15 | 1.03 | 0.81 | 1.23 | 0.10 |
| none | rs6656401 | *CR1* | 1.18 | 1.13 | 0.10 | 0.40 | 0.53 | 1.06 | 0.72 | 1.03 | 0.84 | 1.37 | 0.02 | 1.03 | 0.81 |
| none | rs4147929 | *ABCA7* | 1.15 | 1.21 | 0.01 | 0.54 | 0.50 | 1.23 | 0.12 | 1.30 | 0.09 | 1.37 | 0.06 | 1.02 | 0.88 |
| none | rs17125944 | *FERMT2* | 1.14 | 1.11 | 0.29 | 0.93 | 0.75 | 1.09 | 0.62 | 1.22 | 0.34 | 1.13 | 0.52 | 1.01 | 0.94 |
| none | rs9271192 | *HLA-DRB5-HLA-DRB1* | 1.11 | 1.02 | 0.74 | 0.34 | 0.26 | 0.90 | 0.42 |  |  | 1.16 | 0.20 | 1.01 | 0.96 |
| none | rs28834970 | *PTK2B* | 1.10 | 1.09 | 0.15 | 0.08 | 0.86 | 1.32 | 0.01 | 0.88 | 0.30 | 1.00 | 0.97 | 1.16 | 0.20 |
| none | rs10948363 | *CD2AP* | 1.10 | 1.09 | 0.18 | 0.52 | 0.85 | 1.17 | 0.16 | 1.19 | 0.20 | 1.07 | 0.61 | 0.94 | 0.63 |
| none | rs35349669 | *INPP5D* | 1.08 | 1.09 | 0.11 | 0.79 | 0.83 | 1.06 | 0.58 | 1.22 | 0.10 | 1.07 | 0.56 | 1.06 | 0.62 |
| none | rs10838725 | *CELF1* | 1.08 | 1.05 | 0.45 | 0.55 | 0.61 | 1.07 | 0.53 | 0.89 | 0.39 | 1.15 | 0.24 | 1.05 | 0.70 |
| none | rs2718058 | *NME8* | 0.93 | 0.91 | 0.10 | 0.96 | 0.69 | 0.89 | 0.32 | 0.95 | 0.70 | 0.92 | 0.49 | 0.87 | 0.23 |
| none | rs190982 | *MEF2C* | 0.93 | 0.86 | 0.01 | 0.74 | 0.18 | 0.83 | 0.12 | 0.93 | 0.54 | 0.77 | 0.04 | 0.90 | 0.33 |
| none | rs1476679 | *ZCWPW1* | 0.91 | 0.86 | 0.01 | 0.52 | 0.37 | 0.96 | 0.74 | 0.81 | 0.11 | 0.76 | 0.03 | 0.90 | 0.40 |
| none | rs10498633 | *SLC24A4-RIN3* | 0.91 | 0.92 | 0.19 | 0.17 | 0.92 | 0.75 | 0.03 | 0.89 | 0.44 | 0.93 | 0.57 | 1.13 | 0.34 |
| none | rs983392 | *MS4A6A* | 0.90 | 0.89 | 0.03 | 0.90 | 0.78 | 0.94 | 0.57 | 0.86 | 0.24 | 0.89 | 0.31 | 0.84 | 0.12 |
| none | rs11771145 | *EPHA1* | 0.90 | 0.98 | 0.80 | 0.65 | 0.15 | 1.04 | 0.73 | 0.96 | 0.78 | 1.09 | 0.57 | 0.88 | 0.28 |
| none | rs7274581 | *CASS4* | 0.88 | 0.86 | 0.15 | 0.71 | 0.87 | 0.96 | 0.82 | 0.72 | 0.12 | 0.96 | 0.84 | 0.82 | 0.32 |
| none | rs10792832 | *PICALM* | 0.87 | 0.86 | 0.01 | 0.42 | 0.91 | 0.91 | 0.40 | 0.92 | 0.49 | 0.73 | 0.01 | 0.92 | 0.44 |
| none | rs9331896 | *CLU* | 0.86 | 0.91 | 0.14 | 0.16 | 0.36 | 1.15 | 0.33 | 0.88 | 0.30 | 0.76 | 0.02 | 0.95 | 0.67 |
| none | rs11218343 | *SORL1* | 0.77 | 0.89 | 0.47 | 0.51 | 0.38 | 1.00 | 0.99 |  |  | 0.64 | 0.17 | 0.98 | 0.93 |
| mem | rs6733839 | *BIN1* | 1.22 | 1.21 | 0.01 | 0.01 | 0.91 | 1.03 | 0.88 | 0.85 | 0.24 | 1.33 | 0.14 | 1.56 | 0.00 |
| mem | rs6656401 | *CR1* | 1.18 | 1.21 | 0.02 | 0.01 | 0.77 | 1.88 | 0.00 | 1.53 | 0.02 | 1.27 | 0.18 | 0.87 | 0.29 |
| mem | rs4147929 | *ABCA7* | 1.15 | 1.22 | 0.02 | 0.58 | 0.53 | 1.10 | 0.67 | 1.22 | 0.26 | 0.93 | 0.78 | 1.34 | 0.02 |
| mem | rs17125944 | *FERMT2* | 1.14 | 1.37 | 0.00 | 0.98 | 0.09 | 1.28 | 0.37 | 1.48 | 0.09 | 1.31 | 0.26 | 1.38 | 0.04 |
| mem | rs9271192 | *HLA-DRB5-HLA-DRB1* | 1.11 | 1.19 | 0.03 | 0.33 | 0.40 | 0.94 | 0.75 |  |  | 1.13 | 0.44 | 1.30 | 0.01 |
| mem | rs28834970 | *PTK2B* | 1.10 | 1.08 | 0.28 | 0.17 | 0.79 | 1.08 | 0.67 | 0.88 | 0.38 | 0.96 | 0.80 | 1.28 | 0.02 |
| mem | rs10948363 | *CD2AP* | 1.10 | 1.03 | 0.68 | 0.98 | 0.38 | 0.96 | 0.82 | 1.05 | 0.77 | 1.05 | 0.76 | 1.04 | 0.73 |
| mem | rs35349669 | *INPP5D* | 1.08 | 1.08 | 0.26 | 0.41 | 0.98 | 1.16 | 0.39 | 1.26 | 0.08 | 1.08 | 0.62 | 0.95 | 0.65 |
| mem | rs10838725 | *CELF1* | 1.08 | 1.15 | 0.05 | 0.98 | 0.39 | 1.09 | 0.64 | 1.15 | 0.35 | 1.12 | 0.49 | 1.19 | 0.11 |
| mem | rs2718058 | *NME8* | 0.93 | 0.95 | 0.47 | 0.74 | 0.74 | 1.10 | 0.59 | 0.86 | 0.30 | 0.99 | 0.97 | 0.94 | 0.52 |
| mem | rs190982 | *MEF2C* | 0.93 | 0.85 | 0.02 | 0.49 | 0.22 | 0.76 | 0.17 | 0.86 | 0.31 | 0.70 | 0.04 | 0.93 | 0.50 |
| mem | rs1476679 | *ZCWPW1* | 0.91 | 0.84 | 0.02 | 0.07 | 0.28 | 0.60 | 0.02 | 0.68 | 0.02 | 0.85 | 0.33 | 1.01 | 0.92 |
| mem | rs10498633 | *SLC24A4-RIN3* | 0.91 | 0.91 | 0.24 | 0.90 | 0.99 | 0.80 | 0.27 | 0.94 | 0.72 | 0.97 | 0.86 | 0.91 | 0.44 |
| mem | rs983392 | *MS4A6A* | 0.90 | 0.82 | 0.00 | 0.06 | 0.19 | 0.75 | 0.11 | 0.68 | 0.01 | 1.16 | 0.32 | 0.80 | 0.03 |
| mem | rs11771145 | *EPHA1* | 0.90 | 0.94 | 0.37 | 0.29 | 0.57 | 0.69 | 0.05 | 0.91 | 0.52 | 0.96 | 0.83 | 1.04 | 0.71 |
| mem | rs7274581 | *CASS4* | 0.88 | 0.71 | 0.01 | 0.66 | 0.09 | 0.83 | 0.55 | 0.55 | 0.02 | 0.84 | 0.55 | 0.72 | 0.08 |
| mem | rs10792832 | *PICALM* | 0.87 | 0.83 | 0.01 | 0.97 | 0.47 | 0.81 | 0.24 | 0.85 | 0.25 | 0.78 | 0.11 | 0.84 | 0.11 |
| mem | rs9331896 | *CLU* | 0.86 | 0.95 | 0.47 | 0.79 | 0.17 | 0.97 | 0.91 | 0.88 | 0.38 | 0.87 | 0.39 | 1.02 | 0.84 |
| mem | rs11218343 | *SORL1* | 0.77 | 0.73 | 0.13 | 0.28 | 0.79 | 0.31 | 0.11 |  |  | 1.07 | 0.86 | 0.67 | 0.13 |
| lan | rs6733839 | *BIN1* | 1.22 | 1.03 | 0.78 | 0.59 | 0.11 | 0.75 | 0.32 | 0.95 | 0.80 | 1.09 | 0.63 | 1.19 | 0.39 |
| lan | rs6656401 | *CR1* | 1.18 | 1.33 | 0.01 | 0.11 | 0.30 | 1.43 | 0.27 | 1.63 | 0.10 | 1.53 | 0.01 | 0.80 | 0.34 |
| lan | rs4147929 | *ABCA7* | 1.15 | 1.17 | 0.22 | 0.56 | 0.91 | 1.20 | 0.56 | 0.96 | 0.89 | 1.01 | 0.96 | 1.48 | 0.07 |
| lan | rs17125944 | *FERMT2* | 1.14 | 0.88 | 0.45 | 0.36 | 0.13 | 1.04 | 0.94 | 1.22 | 0.59 | 0.90 | 0.65 | 0.46 | 0.07 |
| lan | rs9271192 | *HLA-DRB5-HLA-DRB1* | 1.11 | 0.92 | 0.43 | 0.43 | 0.09 | 0.69 | 0.22 |  |  | 0.89 | 0.43 | 1.10 | 0.65 |
| lan | rs28834970 | *PTK2B* | 1.10 | 1.15 | 0.14 | 0.37 | 0.65 | 1.50 | 0.10 | 0.94 | 0.78 | 1.04 | 0.76 | 1.35 | 0.12 |
| lan | rs10948363 | *CD2AP* | 1.10 | 1.28 | 0.01 | 0.09 | 0.13 | 2.09 | 0.00 | 1.40 | 0.19 | 1.21 | 0.19 | 0.96 | 0.82 |
| lan | rs35349669 | *INPP5D* | 1.08 | 0.97 | 0.76 | 0.11 | 0.26 | 1.09 | 0.70 | 1.52 | 0.07 | 0.90 | 0.45 | 0.76 | 0.15 |
| lan | rs10838725 | *CELF1* | 1.08 | 1.04 | 0.68 | 0.90 | 0.69 | 0.94 | 0.81 | 0.93 | 0.78 | 1.12 | 0.43 | 1.02 | 0.91 |
| lan | rs2718058 | *NME8* | 0.93 | 0.89 | 0.21 | 0.43 | 0.64 | 0.68 | 0.16 | 0.69 | 0.13 | 0.96 | 0.78 | 1.00 | 0.99 |
| lan | rs190982 | *MEF2C* | 0.93 | 0.86 | 0.11 | 0.79 | 0.41 | 0.82 | 0.47 | 0.75 | 0.23 | 0.96 | 0.76 | 0.80 | 0.22 |
| lan | rs1476679 | *ZCWPW1* | 0.91 | 1.15 | 0.15 | 0.18 | 0.02 | 1.75 | 0.02 | 1.34 | 0.22 | 0.98 | 0.88 | 1.06 | 0.77 |
| lan | rs10498633 | *SLC24A4-RIN3* | 0.91 | 1.10 | 0.37 | 0.48 | 0.07 | 1.02 | 0.95 | 0.75 | 0.32 | 1.24 | 0.14 | 1.09 | 0.68 |
| lan | rs983392 | *MS4A6A* | 0.90 | 0.98 | 0.82 | 0.09 | 0.36 | 0.85 | 0.51 | 0.99 | 0.96 | 1.22 | 0.14 | 0.69 | 0.05 |
| lan | rs11771145 | *EPHA1* | 0.90 | 1.04 | 0.66 | 0.30 | 0.14 | 1.42 | 0.13 | 1.21 | 0.40 | 0.98 | 0.91 | 0.83 | 0.35 |
| lan | rs7274581 | *CASS4* | 0.88 | 0.90 | 0.51 | 0.96 | 0.89 | 0.71 | 0.48 | 0.91 | 0.81 | 0.96 | 0.87 | 0.88 | 0.69 |
| lan | rs10792832 | *PICALM* | 0.87 | 1.00 | 0.97 | 0.87 | 0.14 | 0.86 | 0.54 | 1.14 | 0.57 | 0.98 | 0.90 | 1.02 | 0.93 |
| lan | rs9331896 | *CLU* | 0.86 | 0.97 | 0.75 | 0.31 | 0.22 | 1.10 | 0.75 | 0.66 | 0.10 | 1.11 | 0.46 | 0.89 | 0.54 |
| lan | rs11218343 | *SORL1* | 0.77 | 1.02 | 0.92 | 0.14 | 0.24 | 1.66 | 0.32 |  |  | 1.12 | 0.72 | 0.37 | 0.10 |
| vsp | rs6733839 | *BIN1* | 1.22 | 1.21 | 0.06 | 0.32 | 0.96 | 1.28 | 0.25 | 0.97 | 0.85 | 1.18 | 0.48 | 1.59 | 0.02 |
| vsp | rs6656401 | *CR1* | 1.18 | 1.42 | 0.00 | 0.50 | 0.09 | 1.52 | 0.09 | 1.83 | 0.01 | 1.31 | 0.21 | 1.18 | 0.43 |
| vsp | rs4147929 | *ABCA7* | 1.15 | 1.28 | 0.03 | 0.71 | 0.36 | 1.40 | 0.14 | 1.30 | 0.26 | 0.93 | 0.81 | 1.37 | 0.13 |
| vsp | rs17125944 | *FERMT2* | 1.14 | 1.03 | 0.83 | 0.25 | 0.55 | 1.52 | 0.17 | 0.71 | 0.34 | 0.71 | 0.33 | 1.19 | 0.54 |
| vsp | rs9271192 | *HLA-DRB5-HLA-DRB1* | 1.11 | 1.05 | 0.70 | 0.57 | 0.61 | 0.96 | 0.87 |  |  | 1.23 | 0.28 | 0.94 | 0.77 |
| vsp | rs28834970 | *PTK2B* | 1.10 | 0.92 | 0.43 | 0.91 | 0.08 | 0.89 | 0.56 | 1.04 | 0.84 | 0.87 | 0.48 | 0.90 | 0.58 |
| vsp | rs10948363 | *CD2AP* | 1.10 | 1.25 | 0.03 | 0.48 | 0.20 | 1.33 | 0.17 | 1.59 | 0.02 | 1.14 | 0.52 | 1.06 | 0.78 |
| vsp | rs35349669 | *INPP5D* | 1.08 | 1.06 | 0.53 | 0.50 | 0.82 | 1.07 | 0.71 | 0.89 | 0.51 | 1.30 | 0.14 | 1.01 | 0.94 |
| vsp | rs10838725 | *CELF1* | 1.08 | 0.93 | 0.44 | 0.45 | 0.12 | 0.77 | 0.22 | 0.84 | 0.37 | 0.92 | 0.66 | 1.18 | 0.39 |
| vsp | rs2718058 | *NME8* | 0.93 | 1.17 | 0.09 | 0.81 | 0.01 | 1.35 | 0.13 | 1.04 | 0.83 | 1.12 | 0.54 | 1.20 | 0.30 |
| vsp | rs190982 | *MEF2C* | 0.93 | 0.73 | 0.00 | 0.27 | 0.02 | 0.75 | 0.21 | 0.99 | 0.95 | 0.60 | 0.01 | 0.64 | 0.02 |
| vsp | rs1476679 | *ZCWPW1* | 0.91 | 0.93 | 0.45 | 0.04 | 0.87 | 1.01 | 0.95 | 0.58 | 0.01 | 1.31 | 0.15 | 0.84 | 0.38 |
| vsp | rs10498633 | *SLC24A4-RIN3* | 0.91 | 1.06 | 0.58 | 0.33 | 0.16 | 0.77 | 0.25 | 1.25 | 0.28 | 1.26 | 0.26 | 0.97 | 0.89 |
| vsp | rs983392 | *MS4A6A* | 0.90 | 0.96 | 0.66 | 0.84 | 0.48 | 0.91 | 0.62 | 0.87 | 0.45 | 0.97 | 0.88 | 1.08 | 0.64 |
| vsp | rs11771145 | *EPHA1* | 0.90 | 1.23 | 0.04 | 0.45 | 0.00 | 1.25 | 0.25 | 1.30 | 0.16 | 0.83 | 0.49 | 1.36 | 0.08 |
| vsp | rs7274581 | *CASS4* | 0.88 | 0.98 | 0.92 | 0.53 | 0.48 | 0.63 | 0.23 | 1.00 | 1.00 | 1.30 | 0.39 | 0.97 | 0.91 |
| vsp | rs10792832 | *PICALM* | 0.87 | 0.91 | 0.33 | 0.89 | 0.61 | 0.98 | 0.90 | 0.87 | 0.49 | 0.83 | 0.31 | 0.98 | 0.92 |
| vsp | rs9331896 | *CLU* | 0.86 | 0.99 | 0.94 | 0.16 | 0.16 | 1.52 | 0.10 | 0.75 | 0.13 | 1.07 | 0.74 | 0.96 | 0.82 |
| vsp | rs11218343 | *SORL1* | 0.77 | 0.61 | 0.12 | 0.64 | 0.47 | 0.56 | 0.34 |  |  | 0.84 | 0.72 | 0.41 | 0.14 |
| mix | rs6733839 | *BIN1* | 1.22 | 1.35 | 0.04 | 0.34 | 0.50 | 0.66 | 0.44 | 0.95 | 0.85 | 1.63 | 0.04 | 1.40 | 0.15 |
| mix | rs6656401 | *CR1* | 1.18 | 1.11 | 0.54 | 0.68 | 0.69 | 0.86 | 0.81 | 0.87 | 0.75 | 1.27 | 0.30 | 1.01 | 0.98 |
| mix | rs4147929 | *ABCA7* | 1.15 | 1.30 | 0.13 | 0.27 | 0.48 | 0.72 | 0.60 | 0.87 | 0.73 | 1.82 | 0.03 | 1.15 | 0.61 |
| mix | rs17125944 | *FERMT2* | 1.14 | 0.96 | 0.88 | 0.06 | 0.49 | 0.84 | 0.82 | 1.91 | 0.13 | 0.88 | 0.72 | 0.34 | 0.07 |
| mix | rs9271192 | *HLA-DRB5-HLA-DRB1* | 1.11 | 1.22 | 0.20 | 0.04 | 0.55 | 0.69 | 0.46 |  |  | 1.56 | 0.02 | 0.80 | 0.38 |
| mix | rs28834970 | *PTK2B* | 1.10 | 1.51 | 0.00 | 0.94 | 0.02 | 1.65 | 0.24 | 1.65 | 0.09 | 1.45 | 0.05 | 1.52 | 0.07 |
| mix | rs10948363 | *CD2AP* | 1.10 | 1.06 | 0.68 | 0.71 | 0.80 | 0.99 | 0.98 | 1.26 | 0.49 | 1.11 | 0.63 | 0.91 | 0.71 |
| mix | rs35349669 | *INPP5D* | 1.08 | 0.91 | 0.47 | 0.14 | 0.19 | 1.30 | 0.54 | 1.33 | 0.31 | 0.96 | 0.85 | 0.65 | 0.06 |
| mix | rs10838725 | *CELF1* | 1.08 | 1.05 | 0.74 | 0.87 | 0.82 | 1.24 | 0.62 | 1.15 | 0.65 | 1.08 | 0.71 | 0.95 | 0.83 |
| mix | rs2718058 | *NME8* | 0.93 | 0.78 | 0.06 | 0.62 | 0.19 | 0.69 | 0.42 | 0.69 | 0.24 | 0.89 | 0.55 | 0.68 | 0.10 |
| mix | rs190982 | *MEF2C* | 0.93 | 1.25 | 0.10 | 0.84 | 0.03 | 0.99 | 0.98 | 1.22 | 0.51 | 1.38 | 0.13 | 1.15 | 0.50 |
| mix | rs1476679 | *ZCWPW1* | 0.91 | 0.83 | 0.20 | 0.57 | 0.52 | 0.64 | 0.38 | 0.62 | 0.19 | 0.95 | 0.81 | 0.77 | 0.32 |
| mix | rs10498633 | *SLC24A4-RIN3* | 0.91 | 0.80 | 0.18 | 0.18 | 0.44 | 0.55 | 0.29 | 0.42 | 0.06 | 0.75 | 0.22 | 1.09 | 0.75 |
| mix | rs983392 | *MS4A6A* | 0.90 | 0.92 | 0.53 | 0.21 | 0.86 | 0.92 | 0.85 | 0.65 | 0.17 | 1.16 | 0.44 | 0.80 | 0.31 |
| mix | rs11771145 | *EPHA1* | 0.90 | 1.12 | 0.41 | 0.39 | 0.12 | 1.84 | 0.13 | 0.85 | 0.62 | 1.02 | 0.92 | 1.39 | 0.13 |
| mix | rs7274581 | *CASS4* | 0.88 | 0.88 | 0.60 | 0.85 | 0.98 | 2.73 | 0.05 | 0.73 | 0.57 | 0.83 | 0.60 | 1.03 | 0.93 |
| mix | rs10792832 | *PICALM* | 0.87 | 0.83 | 0.17 | 0.23 | 0.73 | 2.26 | 0.06 | 0.85 | 0.62 | 1.01 | 0.95 | 0.59 | 0.03 |
| mix | rs9331896 | *CLU* | 0.86 | 0.87 | 0.32 | 0.35 | 0.93 | 1.89 | 0.27 | 0.56 | 0.08 | 0.98 | 0.91 | 0.92 | 0.72 |
| mix | rs11218343 | *SORL1* | 0.77 | 0.83 | 0.64 | 0.73 | 0.83 | 2.09 | 0.35 |  |  | 0.73 | 0.57 | 0.95 | 0.92 |

**References**

1. Muthén LK, Muthén BO. Mplus: statistical analysis with latent variables. 5.1 edn. Muthén & Muthén: Los Angeles, CA, 1998-2007.

2. Muthén L, Muthén B. Mplus users guide, version 4.1. Muthen and Muthen: Los Angeles, CA, 2006.

3. Hu L-t, Bentler PM. Cutoff criteria for fit indexes in covariance structure analysis: conventional criteria versus new alternatives. *Structural Equation Modeling* 1999; **6**(1)**:** 1-55.

4. Reeve BB, Hays RD, Bjorner JB, Cook KF, Crane PK, Teresi JA *et al.* Psychometric evaluation and calibration of health-related quality of life item banks: plans for the Patient-Reported Outcomes Measurement Information System (PROMIS). *Med Care* 2007; **45**(5 Suppl 1)**:** S22-31.

5. Escott-Price V, Bellenguez C, Wang LS, Choi SH, Harold D, Jones L *et al.* Gene-wide analysis detects two new susceptibility genes for Alzheimer's disease. *PLoS One* 2014; **9**(6)**:** e94661.

6. Kukull WA, Higdon R, Bowen JD, McCormick WC, Teri L, Schellenberg GD *et al.* Dementia and Alzheimer disease incidence: a prospective cohort study. *Arch Neurol* 2002; **59**(11)**:** 1737-1746.

7. Larson EB, Wang L, Bowen JD, McCormick WC, Teri L, Crane P *et al.* Exercise is associated with reduced risk for incident dementia among persons 65 years of age and older. *Ann Intern Med* 2006; **144**(2)**:** 73-81.

8. McKhann G, Drachman D, Folstein M, Katzman R, Price D, Stadlan EM. Clinical diagnosis of Alzheimer's disease: report of the NINCDS-ADRDA Work Group under the auspices of Department of Health and Human Services Task Force on Alzheimer's Disease. *Neurology* 1984; **34**(7)**:** 939-944.

9. Jun G, Naj AC, Beecham GW, Wang LS, Buros J, Gallins PJ *et al.* Meta-analysis confirms CR1, CLU, and PICALM as alzheimer disease risk loci and reveals interactions with APOE genotypes. *Arch Neurol* 2010; **67**(12)**:** 1473-1484.

10. Petersen RC, Aisen PS, Beckett LA, Donohue MC, Gamst AC, Harvey DJ *et al.* Alzheimer's Disease Neuroimaging Initiative (ADNI): clinical characterization. *Neurology* 2010; **74**(3)**:** 201-209.

11. Saykin AJ, Shen L, Foroud TM, Potkin SG, Swaminathan S, Kim S *et al.* Alzheimer's Disease Neuroimaging Initiative biomarkers as quantitative phenotypes: Genetics core aims, progress, and plans. *Alzheimers Dement* 2010; **6**(3)**:** 265-273.

12. Bennett DA, Wilson RS, Schneider JA, Evans DA, Beckett LA, Aggarwal NT *et al.* Natural history of mild cognitive impairment in older persons. *Neurology* 2002; **59**(2)**:** 198-205.

13. Bennett DA, Schneider JA, Bienias JL, Evans DA, Wilson RS. Mild cognitive impairment is related to Alzheimer disease pathology and cerebral infarctions. *Neurology* 2005; **64**(5)**:** 834-841.

14. Bennett DA, Schneider JA, Buchman AS, Mendes de Leon C, Bienias JL, Wilson RS. The Rush Memory and Aging Project: study design and baseline characteristics of the study cohort. *Neuroepidemiology* 2005; **25**(4)**:** 163-175.

15. Schneider JA, Arvanitakis Z, Bang W, Bennett DA. Mixed brain pathologies account for most dementia cases in community-dwelling older persons. *Neurology* 2007; **69**(24)**:** 2197-2204.

16. Kamboh MI, Minster RL, Demirci FY, Ganguli M, Dekosky ST, Lopez OL *et al.* Association of CLU and PICALM variants with Alzheimer's disease. *Neurobiol Aging* 2012; **33**(3)**:** 518-521.

17. Lambert JC, Ibrahim-Verbaas CA, Harold D, Naj AC, Sims R, Bellenguez C *et al.* Meta-analysis of 74,046 individuals identifies 11 new susceptibility loci for Alzheimer's disease. *Nature Genetics* 2013; **45**(12)**:** 1452-U1206.

18. Manichaikul A, Mychaleckyj JC, Rich SS, Daly K, Sale M, Chen WM. Robust relationship inference in genome-wide association studies. *Bioinformatics* 2010; **26**(22)**:** 2867-2873.

19. Chang CC, Chow CC, Tellier LC, Vattikuti S, Purcell SM, Lee JJ. Second-generation PLINK: rising to the challenge of larger and richer datasets. *Gigascience* 2015; **4:** 7.

20. Willer CJ, Li Y, Abecasis GR. METAL: fast and efficient meta-analysis of genomewide association scans. *Bioinformatics* 2010; **26**(17)**:** 2190-2191.

21. Pruim RJ, Welch RP, Sanna S, Teslovich TM, Chines PS, Gliedt TP *et al.* LocusZoom: regional visualization of genome-wide association scan results. *Bioinformatics* 2010; **26**(18)**:** 2336-2337.

22. Purcell S, Neale B, Todd-Brown K, Thomas L, Ferreira MA, Bender D *et al.* PLINK: a tool set for whole-genome association and population-based linkage analyses. *Am J Hum Genet* 2007; **81**(3)**:** 559-575.

23. DeMichele-Sweet MAA, Weamer EA, Klei L, Vrana DT, Hollingshead DJ, Seltman HJ *et al.* Genetic risk for schizophrenia and psychosis in Alzheimer disease. *Mol Psychiatry* 2017.

24. Lambert JC, Ibrahim-Verbaas CA, Harold D, Naj AC, Sims R, Bellenguez C *et al.* Meta-analysis of 74,046 individuals identifies 11 new susceptibility loci for Alzheimer's disease. *Nat Genet* 2013; **45**(12)**:** 1452-1458.

25. Schott JM, Crutch SJ, Carrasquillo MM, Uphill J, Shakespeare TJ, Ryan NS *et al.* Genetic risk factors for the posterior cortical atrophy variant of Alzheimer's disease. *Alzheimers Dement* 2016; **12**(8)**:** 862-871.
